# Phosphorylation determines the glucose metabolism reprogramming and tumor-promoting activity of sine oculis homeobox 1

**DOI:** 10.1038/s41392-024-02034-5

**Published:** 2024-12-02

**Authors:** Yanni Lin, Ling Li, Bin Yuan, Fei Luo, Xiujuan Zhang, Yuanjun Yang, Shaliu Luo, Jing Lin, Tianxing Ye, Youzhi Zhang, Shan Gao, Qinong Ye

**Affiliations:** 1 School of Basic Medical Sciences, Shanxi Medical University, Taiyuan, Shanxi 030000 China; 2 Department of Cell Engineering, Beijing Institute of Biotechnology, Beijing, 100850 China; 3 Department of Pharmacology, School of Basic Medical Sciences, Anhui Medical University, Hefei, Anhui 230032 China; 4 Medical School of Guizhou University, Guiyang, Guizhou 550025 China; 5 Department of Clinical Laboratory, The Fourth Medical Center of PLA General Hospital, Beijing, 100037 China; 6 Beijing Institute of Pharmacology and Toxicology, Beijing, 100850 China; 7 Zhongda Hospital School of Life Sciences and Technology, Advanced Institute for Life and Health, Southeast University, Nanjing, Jiangsu 210096 China

**Keywords:** Cancer metabolism, Biochemistry, Cell biology

## Abstract

Aerobic glycolysis is a hallmark of cancer and is regulated by growth factors, protein kinases and transcription factors. However, it remains poorly understood how these components interact to regulate aerobic glycolysis coordinately. Here, we show that sine oculis homeobox 1 (SIX1) phosphorylation integrates growth factors (e.g. TGFβ, EGF) to control aerobic glycolysis and determines its tumor-promoting activity. SIX1 is phosphorylated at serine 225 (S225) by growth factors-activated protein kinases ERK1/2 and its phosphorylation is responsible for glycolysis stimulated by some growth factors. SIX1 is dephosphorylated by the atypical protein phosphatase eyes absent 4 (EYA4). Phosphorylation blocks non-canonical ubiquitination and degradation of SIX1 through the E3 ubiquitin ligase FZR1. Unexpectedly, the non-canonical phosphorylation mimic SIX1 (S225K), but not the canonical phosphorylation mimic SIX1 (S225D/E), phenocopies the effects of SIX1 phosphorylation on glycolysis and cancer cell growth and metastasis in vitro and in mice. Compared to normal liver tissues, SIX1 phosphorylation at S225 (pS225) is upregulated in human liver cancer tissues. ERK1/2 expression is positively correlated with pS225 and EYA4 expression is negatively associated with pS225 in liver cancer specimens. Moreover, low expression of pS225 had longer disease-free survival and overall survival in patients with liver cancer. Thus, we identify a common mechanism underlying growth factors-mediated glycolysis, and provide a previously unidentified mode for non-classical phosphorylation mimics of a protein. Targeting growth factors/SIX1 signaling pathway may be beneficial to cancer treatment.

## Introduction

Glucose metabolism reprogramming is a hallmark of cancer.^1–3^ The majority of glucose in cancer cells is metabolized to produce lactate despite abundant oxygen, a phenomenon called the “Warburg effect” or “aerobic glycolysis”. Compared with mitochondrial oxidative phosphorylation, aerobic glycolysis has a faster ATP production rate but it is very inefficient, causing elevated glucose uptake and production of lactate and glycolytic intermediates for biosynthetic pathways in cancer cells. Aerobic glycolysis, which contributes to tumor growth and metastasis, is regulated by a complex interplay of growth factors, protein kinases and transcription factors.^4–8^ Growth factors, including epidermal growth factor (EGF) and insulin-like growth factor 1 (IGF1),^9,10^ key players of cancer cell proliferation and metastasis, can activate extracellular signal–regulated protein kinase 1/2 (ERK1/2). ERK1/2 activation phosphorylates and stimulates a number of proteins, including transcription factors c-Myc and hypoxia-inducible factor-1α (HIF-1α), two master regulators of aerobic glycolysis.^11,12^ However, how growth factors modulate aerobic glycolysis through transcription factors is still poorly understood.

Sine oculis homeobox 1 (SIX1) is a member of homeobox transcription factor family playing critical roles in organogenesis and cancer development and progression.^13–16^ Overexpression (OE) of SIX1 has been observed in many human cancers, including breast cancer and liver cancer, and is associated with poor clinical outcomes in cancer patients. SIX1 is a tumor promoter by regulating aerobic glycolysis, cell proliferation, migration, invasion and metastasis.^17–20^ SIX1, another key regulator of aerobic glycolysis, promotes aerobic glycolysis via directly stimulating glycolytic gene transcription.^17^ In addition, SIX1 can undergo ubiquitination and degradation via the ubiquitin–proteasome pathway, which is dependent on the E3 ubiquitin ligase fizzy/cell division cycle 20 related 1 (FZR1, also known as CDH1) although its ubiquitination site is unclear.^21^ SIX1 can also be modified by O-GlcNAcylation and its O-GlcNAcylation inhibits the ubiquitination and degradation of SIX1.^22^ However, post-translational regulation of SIX1 remains largely unknown.

In this study, we show that SIX1 is phosphorylated at serine 225 (S225) by ERK1/2 in response to growth factors and is dephosphorylated by the atypical protein phosphatase eyes absent 4 (EYA4).^23^ Phosphorylation controls aerobic glycolysis and tumor-promoting activity of SIX1.

## Results

### SIX1 is phosphorylated at S225 by ERK1/2

**Fig. 1:**
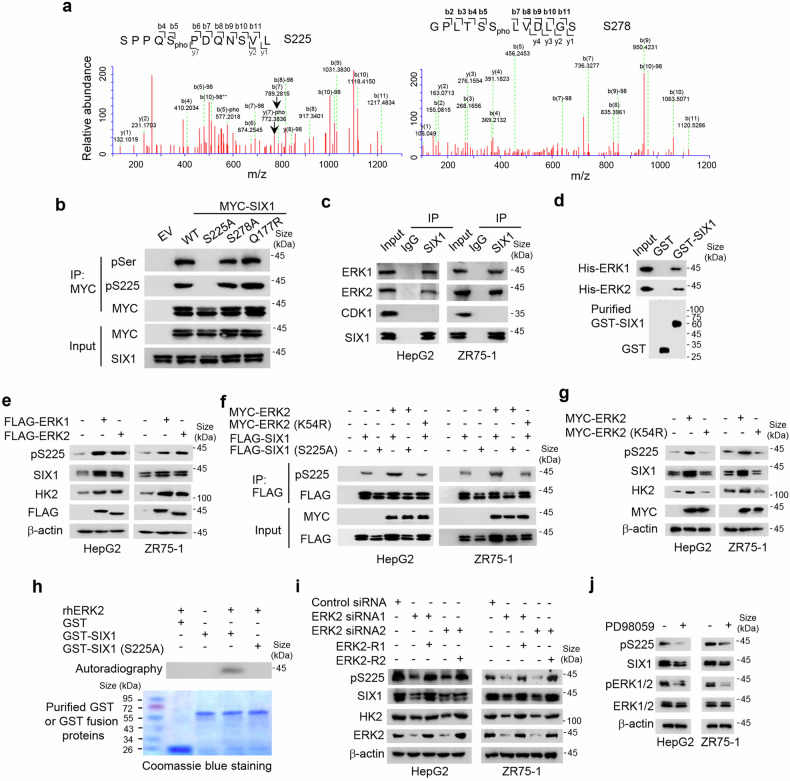
SIX1 is phosphorylated at S225 by ERK. **a** Identification of S225 and S278 of SIX1 as potential phosphorylation sites by NanoLC-MS/MS. pho, phosphorylation. **b** ZR75-1 breast cancer cells were transfected with MYC-tagged SIX1, SIX1 (S225A), SIX1 (S278A) or SIX1 (Q177R). Cell lysates were immunoprecipitated with anti-MYC, followed by immunoblot with the indicated antibodies. pSer, anti-pan-phosphoserine antibody. pS225, anti-SIX1 S225 phosphorylation antibody. IP immunoprecipitation, IB immunoblot, MW molecular weight. **c** Co-immunoprecipitation (Co-IP) analysis of interaction between SIX1 and the indicated proteins in HepG2 and ZR75-1 cells. IgG, normal serum. **d** GST pull-down analysis of interaction between purified GST-SIX1 fusion protein and purified His-tagged ERK1/2. **e** Immunoblot analysis of HepG2 and ZR75-1 cells transfected with FLAG-tagged ERK1/2. β-actin was used as a loading control. **f** HepG2 or ZR75-1 cells were transfected with MYC-tagged ERK2 or ERK2 (K54R) and FLAG-tagged SIX1 or SIX1 (S225A) as indicated. Cell lysates were immunoprecipitated with anti-FLAG, followed by immunoblot with the indicated antibodies. **g** Immunoblot analysis of HepG2 and ZR75-1 cells transfected with MYC-tagged ERK2 or ERK2 (K54R). **h** Purified GST, GST-SIX1 or GST-SIX1 (S225A) was incubated with recombinant human ERK2 (rhERK2) protein in the presence of γ-^32^P-ATP. In vitro kinase assays were performed and analyzed by autoradiography. **i** Immunoblot analysis of HepG2 and ZR75-1 cells transfected with control siRNA, ERK2 siRNA1/2 or ERK2 siRNA1/2 plus siRNA-resistant ERK1/2 (ERK2-R1/2). **j** Immunoblot analysis of HepG2 and ZR75-1 cells treated with 20 μm PD98059

During our experiments, we observed that the human SIX1 protein frequently appeared as a doublet when detected by immunoblot. The upper band of SIX1 disappeared when whole cell lysates from ZR75-1 breast cancer cells, HepG2 liver cancer cells and embryonic kidney HEK293T cells were treated with λ protein phosphatase, suggesting that SIX1 can be phosphorylated (Supplementary Fig. S1a). We purified FLAG-tagged SIX1 from ZR75-1 cells by immunoprecipitation and performed NanoLC-MS/MS analysis on the two bands of SIX1. NanoLC-MS/MS analysis identified S225 and S278 as potential phosphorylation sites of SIX1 (Fig. 1a), one of which was confirmed by constructing SIX1 mutants and performing immunoblot with antibodies against total serine phosphorylation (pSer) or S225 phosphorylation (pS225) (Fig. 1b) or by purifying SIX1 fusion proteins from *E. coli* and mammalian cells and performing immunoblot with antibodies against pS225 (Supplementary Fig. S1b). Substitution of S225, but not S278, with alanine (A) almost eliminated the slower-migrating, upper band of SIX1, and abolished pSer and pS225 signals, and the cancer-related SIX1 (Q177R) mutant had similar pattern to wild-type (WT) SIX1 in terms of pSer and pS225 signals (Fig. 1b).^17^ These data indicates that S225 is the major phosphorylation site of SIX1.

An examination of the amino-acid sequence around the pS225 showed that it contains a consensus P-X-S/T-P motif for phosphorylation by ERK.^24^ As expected, ERK1/2, but not cyclin-dependent kinase 1 (CDK1; in which the consensus phosphorylation motif is S/T-P-X-K/R), physically interacted with SIX1 (Fig. 1c, d, Supplementary Fig. S1c). SIX1 (1–183) encompassing both the highly conserved SIX1 domain (SD) and the DNA-binding homeobox domain (HD) interacted with ERK2 (Supplementary Fig. S1d). ERK1/2 OE increased both pS225 and total SIX1 levels (Fig. 1e, Supplementary Fig. S1e). The increased pS225 was not due to the enhanced SIX1 expression because ERK1/2 OE still increased pS225 after normalization of pS225 with total SIX1 protein (Supplementary Fig. S1f). Moreover, overexpression of WT ERK2, but not the kinase-dead mutant ERK2 (K54R), caused increased pS225 of SIX1, but not SIX1 (S225A) (Fig. 1f,g, Supplementary Fig. S1g, h). As previously reported,^25^ overexpression of ERK2, but not ERK2 (K54R), increased expression of the glycolytic gene *HK2* (Fig. 1g). An in vitro kinase assay showed that recombinant human ERK2 protein directly phosphorylated WT SIX1, but not SIX1 (S225A) (Fig. 1h). In contrast, ERK2 inhibition by ERK2 small interfering RNAs (siRNAs) or the ERK inhibitor PD98059 reduced both pS225 and total SIX1 levels (Fig. 1i, j, Supplementary Fig. S1i, j). ERK2 re-expression in the ERK2 siRNA-transfected cells rescued these effects (Fig. 1i, Supplementary Fig. S1i). Again, the reduced pS225 was not due to the decreased SIX1 expression because ERK1/2 inhibition still reduced pS225 after normalization of pS225 with total SIX1 protein (Supplementary Fig. S1k). Collectively, our data strongly imply that SIX1 is a novel substrate of ERK1/2 and is phosphorylated at S225 by these kinases.

### SIX1 is dephosphorylated by EYA4

**Fig. 2:**
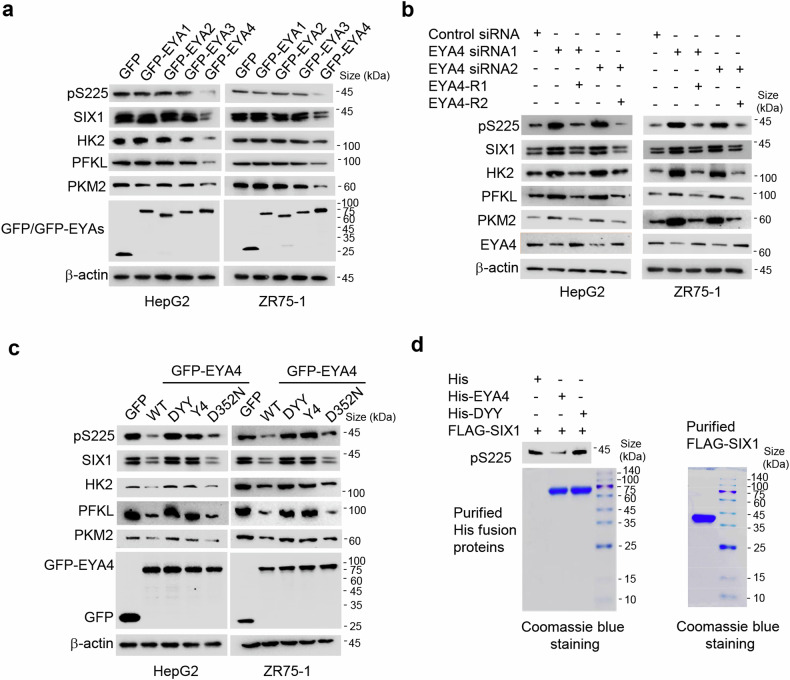
SIX1 is dephosphorylated at S225 by EYA4. **a** Immunoblot analysis of HepG2 and ZR75-1 cells transfected with GFP or GFP-tagged EYA1-4. **b** Immunoblot analysis of HepG2 and ZR75-1 cells transfected with control siRNA, EYA4 siRNA1/2 or EYA4 siRNA1/2 plus siRNA-resistant EYA4 (EYA4-R1/2). **c** Immunoblot analysis of HepG2 and ZR75-1 cells transfected with GFP, GFP-tagged wild-type (WT) EYA4 or GFP-tagged EYA4 mutants (DYY, Y4 and D352N). **d** Immunoblot analysis of purified His-tagged EYA4 or its mutant (DYY) incubated with purified FLAG-SIX1 from HepG2 cells. Coomassie blue staining shows loading levels of the purified proteins

EYA family members were shown to interact with SIX1 and regulate SIX1 transcriptional activity.^14,26^ EYA proteins also have a protein phosphatase function.^23,27^ Thus, we hypothesized that EYA proteins may be involved in SIX1 dephosphorylation. Indeed, overexpression of green fluorescent protein (GFP)-tagged EYA4, but not GFP and GFP-tagged EYA1-3, reduced pS225 in HepG2 and ZR75-1 cells (Fig. 2a). Interestingly, EYA4 OE also inhibited expression of the glycolytic genes *HK2*, *PFKL* and *PKM2* like previously reported SIX1 depletion.^17^ Conversely, EYA4 knockdown (KD) with siRNAs enhanced pS225 and expression of *HK2*, *PFKL* and *PKM2* (Fig. 2b). EYA4 OE decreased SIX1 expression, while EYA4 KD increased SIX1 expression (Fig. 2a, b). The reduced or increased pS225 was not due to the reduced or increased SIX1 expression because EYA4 OE or KD still reduced or increased pS225 after normalization of pS225 with total SIX1 protein (Supplementary Fig. S2a, b). EYA4 KD reduced lactate and ATP levels in HepG2 and ZR75-1 cells (Supplementary Fig. S2c). Re-expression of EYA4 in the EYA4 KD cells rescued such effects. SIX1 (1–124) containing the SD interacted with EYA4 (Supplementary Fig. S2d).

To explore whether intrinsic phosphatase activity of EYA4 is required for SIX1 dephosphorylation, we constructed three EYA4 mutants as previously reported (DYY, Y4 and D352N).^23^ DYY and Y4 have tyrosine phosphatase activity, but they have very weak threonine phosphatase activity, especially for Y4. The D352N mutation results in the loss of tyrosine phosphatase activity, yet its threonine phosphatase activity remains unchanged. Consistent with this, in HepG2 and ZR75-1 cells, DYY and Y4 failed to reduce pS225, but D352N still reduced pS225 (Fig. 2c), suggesting that DYY and Y4 lose their serine-phosphatase activity. DYY and Y4 also failed to decrease *SIX1*, *HK2*, *PFKL* and *PKM2* expression. In vitro experiments showed that purified WT EYA4, but not DYY, directly dephosphorylated purified SIX1 at S225 (Fig. 2d). Altogether, these data suggest that EYA4 is a serine phosphatase for SIX1.

### SIX1 phosphorylation controls its non-canonical ubiquitination and degradation via the ubiquitin-proteasome pathway

**Fig. 3:**
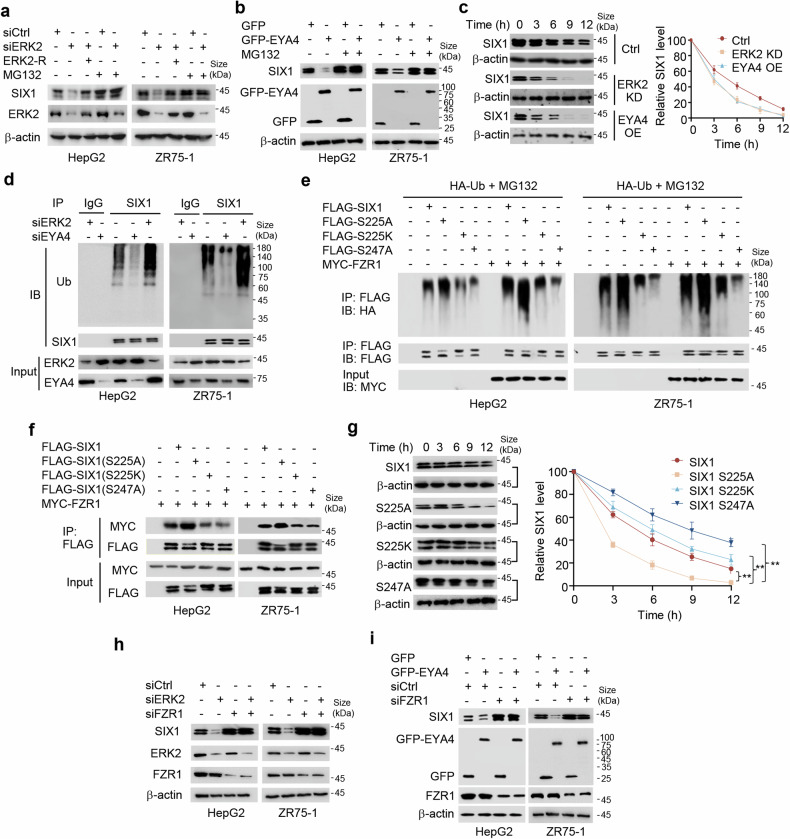
SIX1 phosphorylation regulates its non-canonical ubiquitination and degradation via the ubiquitin-proteasome pathway. **a** Immunoblot analysis of HepG2 and ZR75-1 cells transfected with control siRNA (siCtrl), ERK2 siRNA1 (siERK2) or siERK2 plus ERK2-R1 (ERK2-R) and treated with or without 10 μM MG-132 for 4 h. **b** Immunoblot analysis of HepG2 and ZR75-1 cells transfected with GFP or GFP-EYA4 and treated with or without 10 μM MG-132 for 4 h. **c** Immunoblot analysis of SIX1 expression at the indicated times in HepG2 cells stably transfected with control vector (Ctrl), ERK2 shRNA (ERK2 KD) or GFP-EYA4 (EYA4 OE) and exposure to the protein synthesis inhibitor cycloheximide (20 μg/ml). Graphs show quantification of immunoblot data. Results shown are mean ± SD of 3 independent experiments. **d** Ubiquitination analysis of HepG2 and ZR75-1 cells transfected with siCtrl, siERK2 or siEYA4 and treated with 10 μM MG-132 for 4 h. Ub, ubiquitin. **e** Ubiquitination analysis of HepG2 and ZR75-1 cells transfected with HA-Ub and FLAG-tagged SIX1 or its mutants with or without MYC-tagged FZR1 and treated with 10 μM MG-132 for 4 h. **f** Co-IP analysis of HepG2 and ZR75-1 cells transfected with MYC-tagged FZR1 and FLAG-tagged SIX1 or its mutants. **g** Immunoblot analysis of SIX expression at the indicated times in HepG2 cells stably transfected with FLAG-tagged SIX1, SIX1 (S225A), SIX1 (S225K) and SIX1 (S247A) and exposure to 20 μg/ml cycloheximide. Graphs show quantification of immunoblot data. Results shown are mean ± SD of 3 independent experiments. **P < 0.01. **h** Immunoblot analysis of HepG2 and ZR75-1 cells transfected with siERK2, siFZR1 or siERK2 plus siFZR1 as indicated. **i** Immunoblot analysis of HepG2 and ZR75-1 cells transfected with GFP-EYA4, siFZR1 or GFP-EYA4 plus siFZR1 as indicated

Since ERK and EYA4 regulate both SIX1 phosphorylation and expression, we investigated how ERK and EYA4 modulate SIX1 expression. ERK2 or EYA4 OE and ERK2 or EYA4 KD had no effect on SIX1 mRNA expression (Supplementary Fig. S3a, b), suggesting that ERK2 and EYA4 regulate SIX1 expression at the posttranscriptional level. Indeed, proteasome inhibition by MG132 blocked ERK2 KD or EYA4 OE-mediated reduction of SIX1 expression (Fig. 3a, b), indicating that ERK2 and EYA4 regulate SIX1 expression via the ubiquitin-proteasome pathway. ERK2 re-expression in the ERK2 KD cells reversed ERK2 KD-mediated SIX1 protein reduction. ERK2 KD or EYA4 OE decreased the half-life of SIX1 protein (Fig. 3c). ERK2 KD increased SIX1 ubiquitination, whereas EYA4 KD decreased SIX1 ubiquitination (Fig. 3d).

To define SIX1 ubiquitination sites, we first determined which region may be responsible for SIX1 ubiquitination. SIX1 (1-284), SIX1 (11-284) and SIX1 (61-284), but not SIX1 (1-124) and SIX1 (1-183), were involved in SIX1 ubiquitination (Supplementary Fig. S3c), indicating that the region of 184-284 amino acid (aa) is essential for SIX1 ubiquitination. There are three lysine (K) residues, which are potential canonical ubiquitination sites, within 184-284 aa. However, the mutation of the individual lysine residues to arginine (R) had no effect on SIX1 ubiquitination or even increased SIX1 ubiquitination (Supplementary Fig. S3d). The increased SIX1 ubiquitination might be that this lysine (K199) changes conformation of SIX1 or increased SIX1 stability after potential post-translational modifications except ubiquitination. To further narrow down SIX1 ubiquitination region, we constructed the deletion mutants SIX1 (△184-214), SIX1 (△215-245) and SIX1 (△246-284). SIX1 (△246-284) showed dramatically reduced ubiquitination (Supplementary Fig. S3e), suggesting that 246-284 aa is crucial for SIX1 ubiquitination. Serine (S) and threonine (T) residues are non-canonical ubiquitination sites.^28^ Thus, we mutated all serine and threonine residues to alanine (A) within the amino acid region 246-284. Interestingly, SIX1 (S247A) demonstrated dramatically decreased ubiquitination (Supplementary Fig. S3f), suggesting that S247 is the ubiquitination site of SIX1.

Since it has been reported that protein phosphorylation can regulate ubiquitination,^29^ we investigated whether SIX1 phosphorylation affects its ubiquitination. We transfected WT SIX1, the phosphorylation-deficient SIX1 (S225A) mutant or the phosphorylation-mimetic SIX1 (S225K) mutant into HepG2 or ZR75-1 cells, with SIX1 (S247A) as a control. As expected, SIX1 (S247A) had decreased ubiquitination compared with WT SIX1 (Fig. 3e and Supplementary Fig. S3g). Importantly, the phosphorylation-deficient SIX1 (S225A) mutant showed increased ubiquitination, whereas the phosphorylation-mimetic SIX1 (S225K) mutant showed reduced ubiquitination, suggesting that SIX1 phosphorylation status regulates its ubiquitination. The E3 ubiquitin ligase FZR1 can ubiquitinate SIX1.^21^ We then tested the effect of FZR1 on WT SIX1, SIX1 (S225A), SIX1 (S225K) and SIX1 (S247A). FZR1 increased ubiquitination of WT SIX1 and SIX1 (S225A), but not SIX1 (S225K) and SIX1 (S247A) (Fig. 3e), further validating that SIX1 phosphorylation status modulates its ubiquitination and S247 is the ubiquitination site of SIX1. Consistent with these results, SIX1 (S225K) and SIX1 (S247A) had weaker interaction with FZR1 than WT SIX1 and SIX1 (S225A) (Fig. 3f and Supplementary Fig. S3h), and SIX1 (S225K) and SIX1 (S247A) had longer half-life than WT SIX1 and SIX1 (S225A) (Fig. 3g). KD of FZR1 eliminated the capacity of ERK2 KD or EYA4 OE to reduce SIX1 expression (Fig. 3h, i). All together, these results collectively suggest that ERK2 and EYA4 regulate SIX1 expression through FZR1-mediated SIX1 ubiquitination and degradation.

### SIX1 is phosphorylated in response to growth factors and critical for glycolysis mediated by some growth factors

**Fig. 4:**
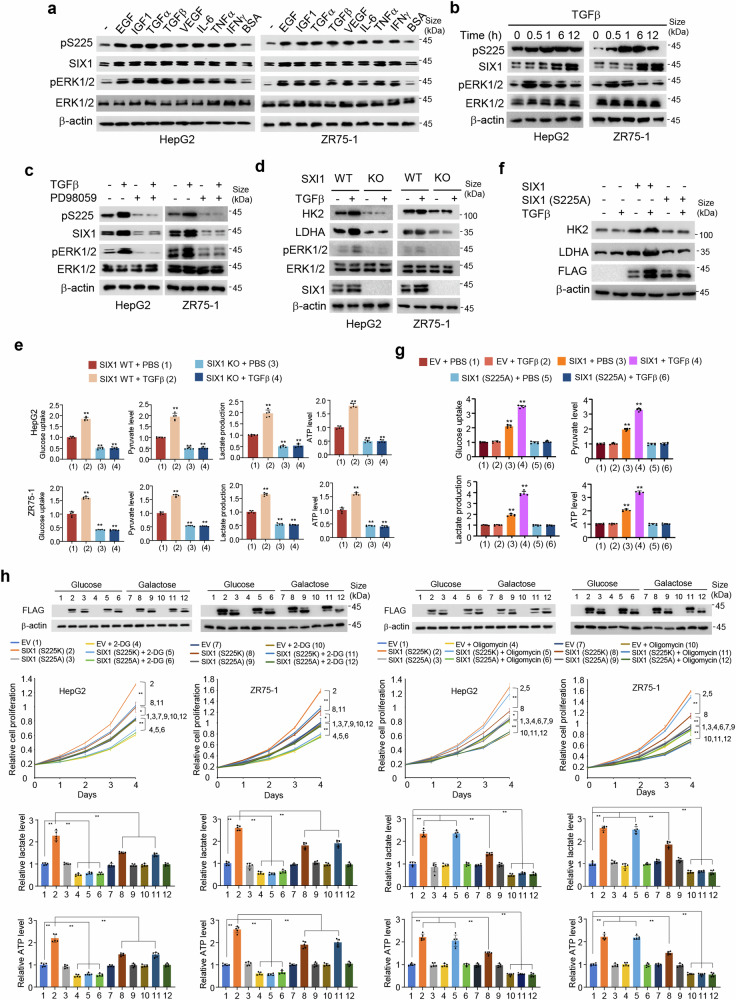
SIX1 is phosphorylated in response to growth factors and important for growth factor-mediated glycolysis. **a** Immunoblot analysis of HepG2 and ZR75-1 cells treated with 50 ng/ml EGF, 100 ng/ml IGF1, 100 ng/ml TGFα, 5 ng/ml TGFβ, 10 ng/ml VEGF, 100 ng/ml IL-6, 20 ng/ml TNFα and 500 U/ml IFNγ for 12 h with BSA as a control. BSA, bovine serum albumin. **b** Immunoblot analysis of HepG2 and ZR75-1 cells treated with 5 ng/ml TGFβ for the indicated times. **c** Immunoblot analysis of HepG2 and ZR75-1 cells pretreated with 20 μm PD98059 and then treated with 5 ng/ml TGFβ for 12 h. **d** Immunoblot analysis of SIX1 WT or KO HepG2 or ZR75-1 cells treated with 5 ng/ml TGFβ for 12 h. **e** Analysis of glucose uptake and production of pyruvate, lactate and ATP in SIX1 WT or KO HepG2 or ZR75-1 cells treated with or without 5 ng/ml TGFβ for 12 h. PBS, phosphate buffered saline. **f** Immunoblot analysis of SIX1 KO HepG2 cells transfected with empty vector, FLAG-SIX1 or FLAG-SIX1 (S225A) and treated with or without 5 ng/ml TGFβ for 12 h. **g** Analysis of glucose uptake and production of pyruvate, lactate and ATP in cells from **f**. **h** The proliferation curve and lactate and ATP production of ZR75-1 and HepG2 cells transfected with EV or FLAG-tagged SIX1 (S225K) or SIX1 (S225A) and treated with 2.5 mM 2-DG or 0.1 mM Oligomycin in glucose (25 mM) or galactose (10 mM)-containing medium as indicated. Representative immunoblot with anti-FLAG indicates the expression of SIX1 (S225K) or SIX1 (S225A). Data shown are means ± SD of quintuplicate measurements that have been repeated three times with similar results (**e**, **g**). Data shown are mean ± SD of three independent experiments (**h**). Statistical significance was assessed by two-tailed Student’s *t* test. **P < 0.01 versus SIX1 WT cells treated with PBS (**e**) or SIX1 KO HepG2 cells transfected with empty vector and treated with PBS (**g**). *P < 0.05, **P < 0.01 (**h**)

ERK1/2 can be activated by many extracellular signaling molecules, including EGF, IGF1, transforming growth factor α (TGFα), TGFβ, vascular endothelial growth factor (VEGF), interleukin-6 (IL-6), tumor necrosis factor α (TNFα), and interferon γ (IFNγ).^30–36^ Since SIX1 is phosphorylated by ERK1/2, we tested whether SIX1 is phosphorylated in response to these growth factors/cytokines. As expected, EGF, IGF1, TGFα, TGFβ, VEGF, IL-6, TNFα, and IFNγ increased ERK1/2 phosphorylation in HepG2 and ZR75-1 cell lines (Fig. 4a and Supplementary Fig. S4a). Importantly, all growth factors/cytokines tested increased both pS225 and SIX1 levels. As a negative control, bovine serum albumin did not activate SIX1. Consistent with the results of pS225 modulation of SIX1 degradation, TGFβ increased pS225 before it increased SIX1 expression (Fig. 4b and Supplementary Fig. S4b). The ERK inhibitor PD98059 abrogated TGFβ-mediated enhancement of pS225 and SIX1 expression (Fig. 4c and Supplementary Fig. S4c), suggesting that ERK is required for TGFβ modulation of pS225 and SIX1 expression.

As previously reported,^37^ *SIX1* knockout (KO) decreased ERK1/2 phosphorylation (Supplementary Fig. S4d). Intriguingly, SIX1 KO completely abolished the ability of TGFβ to enhance the expression of the glycolytic genes, specifically *HK2* and *LDHA* as well as ERK1/2 phosphorylation (Fig. 4d and Supplementary Fig. S4d), suggesting that SIX1 is required for TGFβ-mediated glycolytic gene expression. SIX1 was largely or partly responsible for EGF-, IGF1-, TGFα-, VEGF-, IL-6-, TNFα-, and IFNγ-mediated glycolytic gene expression, because *SIX1* KO greatly attenuated the ability of these growth factors/cytokines to increase glycolytic gene expression (Supplementary Fig. S4d). In agreement with the results of SIX1 modulation of TGFβ- or EGF-mediated glycolytic gene expression, *SIX1* KO abolished or almost abolished the ability of TGFβ or EGF to enhance glucose uptake and production of pyruvate, lactate and ATP (Fig. 4e, Supplementary Fig. S4e). Taken together, these data suggest that SIX1 is responsible for glycolysis mediated by some growth factors and ERK/SIX1 forms a positive feedback loop.

To further determine whether SIX1 phosphorylation is critical for glycolysis mediated by some growth factors, we tested the effects of TGFβ and EGF on glycolytic gene expression, glucose uptake, and production of pyruvate, lactate and ATP in *SIX1* KO HepG2 cells transfected with SIX1 WT or S225A mutant. As in *SIX1* KO cells, TGFβ and EGF had no or little effects on glycolytic gene expression, glucose uptake, and production of pyruvate, lactate and ATP in SIX1 KO HepG2 cells transfected with SIX1 (S225A) (Fig. 4f, g, Supplementary Fig. S4f-h). In contrast, TGFβ and EGF promoted glycolytic gene expression, glucose uptake, and levels of pyruvate, lactate and ATP in SIX1 KO HepG2 cells transfected with WT SIX1. Combined altogether, these results imply that some growth factors converge on SIX1 phosphorylation to regulate glycolysis.

SIX1-overexpressing cancer cells have a preference for glycolysis over oxidative phosphorylation.^17^ Next, we determined whether cancer cell proliferation and production of lactate and ATP regulated by SIX1 phosphorylation are due to a preference for glycolysis over oxidative phosphorylation. Given the intricate function of TGFβ in cancer progression, which is a tumor suppressor in normal tissues and early lesions and promotes invasion and metastasis in advanced tumors,^38^ we examined the effect of the phosphorylation mimic SIX1 (S225K) and the phosphorylation-deficient mutant SIX1 (S225A) on cancer cell growth, lactate production and ATP generation using media containing either galactose or glucose and devoid of TGFβ. It has been established that cells cultured in galactose rely more heavily on oxidative phosphorylation to produce ATP.^39^ Unsurprisingly, cancer cells cultured in galactose-based media showed comparable proliferative rate to those grown in glucose-containing media (Fig. 4h). However, SIX1 (S225K)-overexpressing cells proliferated more rapidly in glucose-containing media compared to galactose. Notably, this phenotype was largely abolished by 2-DG, the best characterized glycolytic inhibitor, whereas the oxidative phosphorylation inhibitor oligomycin had no such effect. In agreement with the results of cell proliferative capacities, glucose-grown cells overexpressing SIX1 (S225K) generated higher levels of lactate and ATP than those galactose-grown cells (Fig. 4h). Again, 2-DG significantly negated this effect, whereas oligomycin did not. Importantly, SIX1 (S225A) lost these effects. These data suggest that cancer cells overexpressing phosphorylated SIX1 have a preference for glycolysis over oxidative phosphorylation, and elevated glycolysis by SIX1 phosphorylation controls increased ATP production that may support proliferation.

### Non-canonical phosphorylation-mimetic SIX1 mutant promotes glycolysis and tumor growth and metastasis

**Fig. 5:**
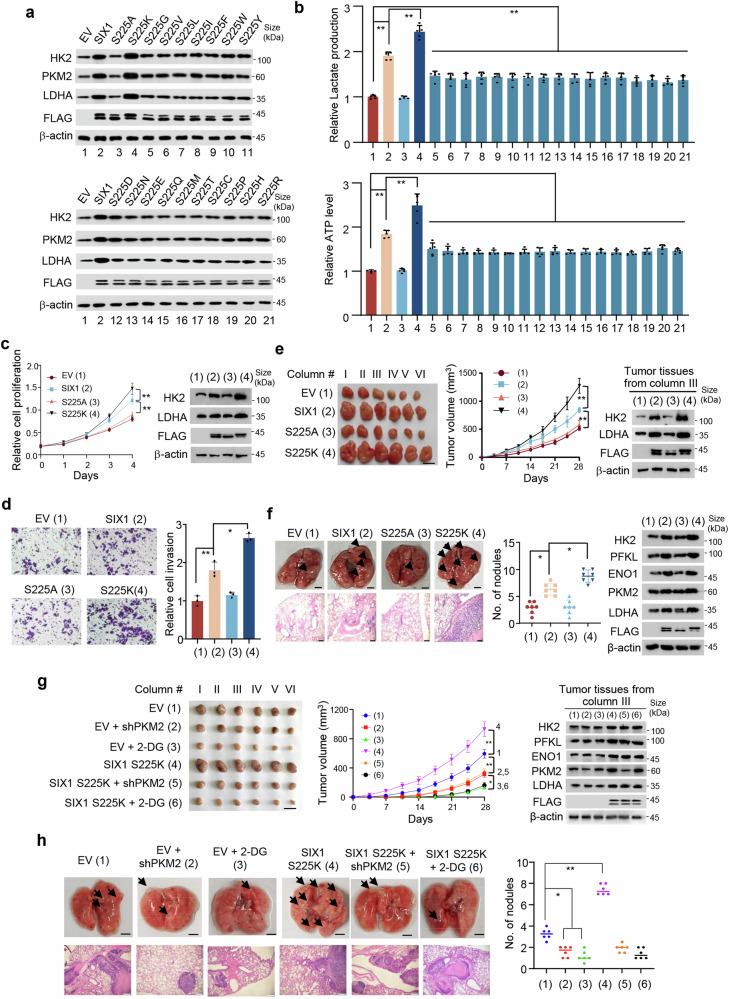
Non-canonical phosphorylation-mimetic SIX1 mutant enhances glycolysis as well as tumor growth and metastasis. **a** Immunoblot analysis of SIX1 KO HepG2 cells transfected with FLAG-SIX1 or its mutants. **b** Analysis of lactate production and ATP level in cells from **a**. Data shown are means ± SD of quintuplicate measurements that have been repeated three times with similar results. Statistical significance was assessed by one-way ANOVA test. *P < 0.05, **P < 0.01. **c** The proliferation curve of SIX1 KO HepG2 cells stably transfected with FLAG-SIX1 or its mutants. Immunoblot shows the expression of HK2, LDHA and FLAG-SIX1 or its mutants. Data shown are means ± SD of three independent experiments. Statistical significance was assessed by one-way ANOVA test. **P < 0.01. **d** Cell invasion assay of SIX1 KO HepG2 cells stably transfected with FLAG-SIX1 or its mutants. The relative cell invasions are shown. Scale bar, 100 μm. Data shown are means ± SD of three independent experiments. Statistical significance was assessed by one-way ANOVA test. *P < 0.05, **P < 0.01. **e** The growth curve of xenograft tumors derived from SIX1 KO HepG2 cells stably transfected with FLAG-SIX1 or its mutants. Representative immunoblot with the indicated antibodies was shown in the representative excised tumors. Scale bar, 10 mm. Statistical significance was assessed by one-way ANOVA test. **P < 0.01 at day 28. **f** Representative lung tissues and hematoxylin and eosin (H&E)-stained sections of the lung tissues at 35 days from nude mice injected by tail vein with SIX1 KO MHCC97-H cells stably transfected with FLAG-SIX1 or its mutants (n = 7). Arrows indicate tumor foci. The number of tumor nodules spread throughout the pulmonary region is shown. Representative immunoblot with the indicated antibodies was shown in the representative excised tumors. Scale bar, 2.5 mm for lung tissues; Scale bar, 50 μm for H&E-stained sections. **g** SIX1 KO MHCC97-H cells stably expressing PKM2 shRNA, FLAG-tagged SIX1 (S225K) or PKM2 shRNA plus FLAG-tagged SIX1 (S225K) were injected into nude mice. 2-DG was used as indicated. Stripped tumors are shown (left). The growth curve was plotted (middle). The expression of the indicated proteins in representative excised tumors from the third column in each group was analyzed by immunoblot (right). Scale bar, 10 mm. *P < 0.05, **P < 0.01 at day 28. **h** Representative lung tissues and H&E-stained sections of the lung tissues at 35 days from nude mice injected by tail vein with SIX1 KO MHCC97-H cells harboring different constructs as in **g**. Arrows denote tumor foci. The number of tumor nodules spread throughout the pulmonary region is indicated. Scale bar, 2.5 mm for lung tissues; Scale bar, 50 μm for H&E-stained sections. Statistical significance was assessed by one-way ANOVA test. *P < 0.05, **P < 0.05

SIX1 is a key regulator of glycolysis as well as tumor growth and metastasis.^17^ Next, we tested the effects of pS225 on these biological functions. Indeed, the phosphorylation-defective SIX1 (S225A) mutant lost its ability to modulate glycolytic gene expression and production of lactate and ATP (Fig. 5a, b, Supplementary Fig. S5a, b). However, the canonical phosphorylation-mimetic SIX1 (S225D/E) mutant failed to mimic effects of SIX1 on glycolysis. Thus, we mutated S to all other amino acid residues. Fascinatingly, the non-canonical phosphorylation-mimetic SIX1 (S225K) mutant, but not the other mutants, could mimic the capability of SIX1 to enhance glycolytic gene expression and production of lactate and ATP (Fig. 5a, b, Supplementary Fig. S5a, b). Moreover, SIX1 (S225K) promoted liver cancer cell proliferation and invasion in vitro and growth and lung metastasis of liver tumor in vivo even more robustly than WT SIX1, while SIX1 (S225A) completely eliminated the capability of SIX1 to facilitate these effects (Fig. 5c–f, Supplementary Fig. S5c–f). As expected, the rate-limiting enzyme PKM2 promotes cancer cell proliferation, and SIX1 (S225A) did not further increase the capacity of PKM2 to modulate cell proliferation (Supplementary Fig. S5e). Compared to WT SIX1-expressing liver tumors and lung metastatic foci, SIX1 (S225K)-expressing liver tumors and lung metastatic foci had increased glycolytic gene expression (Fig. 5e, f, Supplementary Fig. S5f). SIX1 (S225A) had a similar behavior to empty vector in terms of glycolytic gene expression. Moreover, PKM2 KD or the glycolytic inhibitor 2-DG repressed liver tumor growth and metastasis in nude mice, and greatly attenuated the ability of SIX1 (S225K) to promote liver tumor growth and metastasis (Fig. 5g, h). These data suggest that non-canonical phosphorylation-mimetic SIX1 (S225K) mutant can mimic the roles of SIX1 in glycolysis, cell proliferation and invasion, and tumor growth and metastasis.

### Non-canonical phosphorylation-mimetic SIX1 mutant increases its binding to the downstream target gene promoters and transcription of glycolytic genes

To examine how SIX1 phosphorylation affects its function, we tested how WT SIX1, SIX1 (S225A), and SIX1 (S225K) affects the expression of glycolytic genes. SIX1 (S225K) OE in *SIX1* KO HepG2 or ZR75-1 cells caused elevated HK2, PKM2, and LDHA mRNA and protein expression, compared with WT SIX1 (Supplementary Fig. S6a). SIX1 (S225A) OE had no such effect on expression of these glycolytic genes. It is worth noting that the expression levels of WT SIX1, SIX1 (S225A) and SIX1 (S225K) are comparable (Supplementary Fig. S6a). Since WT SIX1 and SIX1 (S225K) regulate glycolytic gene expression at the mRNA level, we examined the effects of WT SIX1, SIX1 (S225A) and SIX1 (S225K) on binding of HK2, PKM2 and LDHA promoters by ChIP experiments. SIX1 (S225K) exhibited a stronger binding affinity for the promoters of HK2, PFKL, ENO1, PKM2, and LDHA than WT SIX1, and SIX1 (S225A) failed to bind to these promoters (Supplementary Fig. S6b). The cellular localization of SIX1 (S225K) and SIX1 (S225A) resembled that of WT SIX1 (Supplementary Fig. S6c, d). SIX1 (S225K) and SIX1 (S225A) also did not alter the ability of SIX1 to interact with the transcriptional co-factors AIB1, HBO1 and EYA4 (Supplementary Fig. S6e,f).^17^ In summary, these results indicate that SIX1 (S225K) enhances the capacity of SIX1 to stimulate glycolytic gene expression by strengthening its binding affinity to the promoters of glycolytic genes.

### Liver-specific knock-in of SIX1 (S225K) in mice accelerates DEN-induced development of hepatocellular carcinoma

**Fig. 6:**
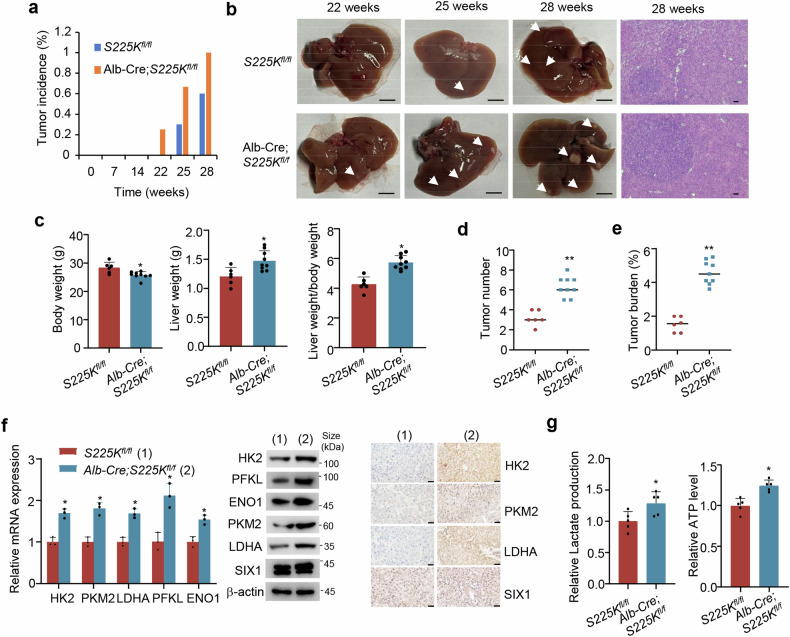
Liver-specific SIX1 (S225K) knock-in promotes DEN-induced development of hepatocellular carcinoma in mice. **a** Tumor incidence of *S225K*^*fl/fl*^ (n = 9) and Alb-Cre;*S225K*^*fl/fl*^ (n = 8) mice at week 22, Tumor incidence of *S225K*^*fl/fl*^ (n = 10) and Alb-Cre;*S225K*^*fl/fl*^ (n = 9) mice at week 25 and Tumor incidence of *S225K*^*fl/fl*^ (n = 10) and Alb-Cre;*S225K*^*fl/fl*^ (n = 9) mice at week 28. Statistical significance was assessed by the chi-square test. *P < 0.05. **b** Representative liver tissues and H&E-stained sections of the liver tissues at week 28 from *S225K*^*fl/fl*^ and *Alb-Cre;S225K*^*fl/fl*^ mice. Scale bar, 5 mm for liver tissues; Scale bar, 100 μm for the H&E-stained sections. **c** Body weight, liver weight and the ratio of liver weight to body weight at week 28 from *S225K*^*fl/fl*^ (n = 10) and *Alb-Cre;S225K*^*fl/fl*^ (n = 9) mice. **d** Quantitation of liver tumor numbers in mouse livers from *S225K*^*fl/fl*^ and *Alb-Cre;S225K*^*fl/fl*^ groups from **c**. **e** Quantitation of total tumor area per mouse liver from *S225K*^*fl/fl*^ and *Alb-Cre;S225K*^*fl/fl*^ groups from **c**. Statistical significance was assessed by two-tailed Student’s *t* test (**c**–**e**). *P < 0.05, **P < 0.01 versus *S225K*^*fl/fl*^. **f** RT-qPCR (left), immunoblot (middle) and immunohistochemical (right) analysis of representative liver tumor tissues from *S225K*^*fl/fl*^ and *Alb-Cre;S225K*^*fl/fl*^ groups. Scale bar, 50 μm. Data shown are means ± SD of three independent experiments. Statistical significance was assessed by two-tailed Student’s *t* test. *P < 0.05 versus *S225K*^*fl/fl*^. **g** Analysis of lactate production and ATP level in representative liver tumor tissues from **f**. Data shown are means ± SD of quintuplicate measurements that have been repeated three times with similar results. Statistical significance was assessed by two-tailed Student’s *t* test. **P < 0.05 versus *S225K*^*fl/fl*^

Diethylnitrosamine (DEN) is often used to induce development of hepatocellular carcinoma in rodents.^40^ To test whether pS225 plays a role in tumorigenesis in vivo, we used DEN to induce hepatocellular carcinoma in mice. We crossed SIX1 (S225K)^*fl/fl*^ mice (S225K^*fl/fl*^) with hepatocyte-specific Albumin-Cre recombinase (Alb-Cre) transgenic mice to obtain liver-specific SIX1 (S225K) knock-in (KI) mice (Alb-Cre;S225K^*fl/fl*^) (Supplementary Fig. S7), and analyzed liver tumor development 22 weeks after DEN treatment, with S225K^*fl/fl*^ mice as controls. 2 of the 8 Alb-Cre;S225K^*fl/fl*^ mice developed liver tumors, whereas none of controls (n = 9) exhibited visible liver tumors (Fig. 6a). At 25 weeks, 6 of the 9 Alb-Cre;S225K^*fl/fl*^ mice showed liver tumor development and 3 of the 10 controls developed liver tumors. At 28 weeks, All Alb-Cre;S225K^*fl/fl*^ mice (n = 9) harbored liver tumors and 6 of the 10 controls developed liver tumors. Liver anatomy and hematoxylin staining of the entire liver tissue sections confirmed the presence of neoplastic lesions (Fig. 6b). In mice that developed liver tumors, compared with the control group, the S225K KI group had decreased body weight, and increased liver mass and a higher ratio of liver mass to body weight (Fig. 6c). In contrast to the control group, the S225K KI group had larger neoplastic lesions, both in terms of the number of tumors and overall tumor burden (Fig. 6d, e). As expected, liver tumor tissues of the Alb-Cre;S225K^*fl/fl*^ group showed higher levels of PFKL, ENO1, HK2, PKM2 and LDHA mRNA and protein expression, lactate and ATP than the S225K^*fl/fl*^ group (Fig. 6f, g). Moreover, RNA sequencing (RNA-seq) using tumor tissues from the Alb-Cre;S225K^*fl/fl*^ and S225K^*fl/fl*^ groups showed that *SIX1 S225K* knock-in regulated expression of many genes, including the glycolytic genes *LDHA*, *ENO1*, and *PKM* (Supplementary Fig. S8). Kyoto Encyclopedia of Genes and Genomes (KEGG) analysis revealed that *SIX1 S225K* knock-in modulated the glycolytic pathway. These data indicate that liver-specific SIX1 (S225K) knock-in accelerates DEN-induced development of hepatocellular carcinoma in mice.

### Clinical relevance of the SIX1 phosphorylation axis in liver cancer

**Fig. 7:**
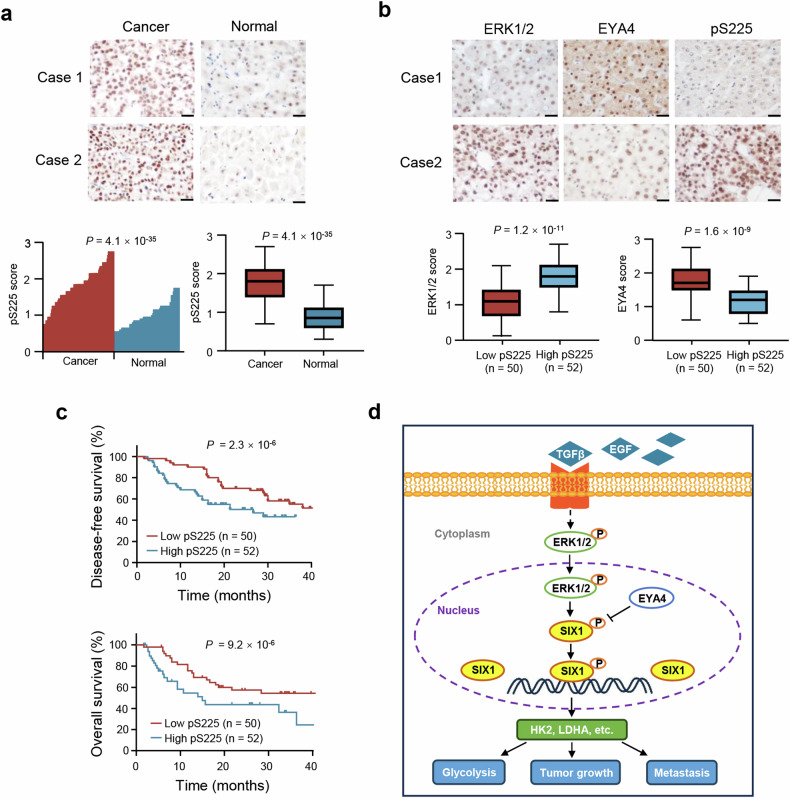
Clinical relevance of the pS225 axis in patients with liver cancer. **a** pS225 expression in 102 cancerous liver tissues and adjacent normal liver tissues was examined by immunohistochemistry (IHC). Relative pS225 expression levels were plotted and compared between normal and cancer tissues (Paired-sample *t*-test). Scale bar, 100 μm. **b** ERK1/2, EYA4 and pS225 expression in 102 patients with liver cancer from **a** were assessed by IHC. The correlation between pS225 and ERK1/2 and EYA4 was examined by Pearson’s correlation test. Case 1 and case 2 refer to two representative samples categorized by low and high expression of the corresponding proteins. Scale bar, 100 μm. **c** The disease-free and overall survival curves related to low and high expression of pS225 were assessed in 102 patients with liver cancer from a using the Kaplan–Meier method. **d** A proposed model underlying the role of SIX1 phosphorylation in growth factors-mediated glycolysis and tumor growth and metastasis. Growth factors (e.g. TGFβ, EGF)-activated ERK1/2 stimulates SIX1 phosphorylation, which is dephosphorylated by EYA4. SIX1 phosphorylation is required for its binding to glycolytic gene promoters, leading to expression of glycolytic genes (e.g. HK2, LDHA) and tumor growth and metastasis

To investigate the clinical significance of the pS225 axis, we performed immunohistochemistry (IHC) of 102 liver cancer tissue samples and matched normal controls. Compared to normal liver tissues, pS225 expression was upregulated in liver cancer tissues (Fig. 7a). The expression of ERK1/2 was positively correlated with pS225 and EYA4 level was negatively associated with pS225 (Fig. 7b). Moreover, low level of pS225 had longer disease-free survival (DFS) and overall survival (OS) in patients with liver cancer (Fig. 7c). Based on ENCORI (Encyclopedia of RNA Interactomes) database (https://rnasysu.com/encori/) in which data of cancers were from TCGA (The Cancer Genome Atlas) project, liver cancer patients exhibiting high SIX1 mRNA expression demonstrated a shorter overall survival rate (Supplementary Fig. S9).

## Discussion

We previously showed that the transcription factor SIX1 is a key regulator of aerobic glycolysis via directly promoting glycolytic gene transcription.^17^ Very recently, SIX1 has been reported to activate the expression of liver X receptors, thus inducing De novo lipogenesis and non-alcoholic fatty liver disease progression.^41^ As an important tumor suppressor, the transcription factor p53 regulates a range of cellular metabolic processes, including the Warburg effect, glutaminolysis, serine/glycine synthesis, fatty acid synthesis, and nucleotide metabolism.^42^ Thus, we cannot exclude the possibility that SIX1 may regulate these metabolic processes, potentially contributing to the regulation of tumor growth and metastasis. In the current study, we identify a phosphor-serine residue (pS225) serving as a molecular switch for the glycolytic and tumor-promoting activities of SIX1 (Fig. 7d). Growth factors/cytokines-activated ERK phosphorylates SIX1 at S225, whereas EYA4 dephosphorylates SIX1 at S225. Except for ERK and EYA4, we cannot exclude the possibility that other protein kinases and phosphatases can also phosphorylate and dephosphorylate SIX1. Importantly, pS225 dictates SIX1 function in glucose metabolism reprogramming as well as tumor growth and metastasis, because the phosphorylation-deficient mutant SIX1 (S225A) abolishes the ability of SIX1 to facilitate glycolytic gene expression, aerobic glycolysis, tumor growth and metastasis. Mechanistically, SIX1 (S225A) loses its ability to bind to the promoter regions of downstream target genes. Aerobic glycolysis is critical for tumor growth and metastasis.^1–3^ It is conceivable that the SIX1 (S225A) mutant loses its ability to regulate aerobic glycolysis, thus losing its ability to modulate tumor growth and metastasis. Many growth factors, including EGF, IGF1 and TGFβ, can activate ERK and enhance glycolytic gene expression, glycolysis, cancer cell proliferation and/or metastasis.^10,30,43^ Intriguingly, SIX1 is required for or largely responsible for the growth factors-mediated enhancement of glycolytic gene expression and glycolysis, suggesting that the ERK/SIX1 axis is critical for growth factors-mediated glycolysis. Activated ERK (ERK phosphorylation) increases SIX1 expression and SIX1 increases ERK phosphorylation as shown in this study and previous studies,^37^ suggesting that the ERK/SIX1 axis forms a positive feedback loop. ERK is widely activated in cancer, rendering it a compelling target for cancer therapy.^44,45^ Currently, a growing number of ERK inhibitors have been developed, with several of them advancing to clinical trials. However, therapeutic resistance to ERK inhibition is a major problem. SIX1 is overexpressed in multiple cancer types, including breast cancer and liver cancer, and its high expression predicts poor clinical outcomes.^13–16^ Therefore, disrupting the ERK/SIX1 interaction may open up a new way for cancer treatment.

EYA proteins, including EYA1-4, are responsible for determining cell fate in various organisms from insects to humans.^46–48^ EYA proteins possess phosphatase activity or function as a co-activator/co-repressor for transcription factors, including SIX1 and estrogen receptor β. In this study, we found that EYA4, but not EYA1-3, dephosphorylates SIX1, indicating the specificity of enzyme-substrate interaction. The C-terminal region of EYA1-4, containing 273 amino acids and being the most conserved sequence among EYA proteins, is designated as the EYA domain, which possesses tyrosine phosphatase activity.^46–48^ However, the N-terminal amino acid homology among EYA proteins is low. The N-terminal domain has demonstrated protein threonine phosphatase activity (either intrinsic or through association with protein phosphatase 2A (PP2A)).^49^ To the best of our knowledge, the serine phosphatase activity of EYA proteins is poorly understood. The observation that EYA4, but not EYA1-3, dephosphorylates SIX1 suggests that the N terminus of EYA4, which have low amino acid homology with other EYA proteins, determines the specificity of enzyme-substrate interaction. We showed that purified EYA4 directly dephosphorylates purified SIX1, indicating that EYA4 may have intrinsic serine phosphatase activity. In nervous system tumors like glioma and malignant peripheral nerve sheath tumor, EYA4 functions as an oncogene. Conversely, in non-nervous system cancers, including liver cancer, esophageal squamous cell carcinoma, and colorectal cancer, EYA4 serves as a tumor suppressor.^44^ EYA4 inhibits hepatocellular carcinoma growth, invasion and metastasis by repressing c-myc binding protein (MYCBP), the nuclear factor-κB (NF-κB)/RAS-related protein 1 (Rap1) signaling pathway, or the c-Jun/vascular endothelial growth factor (VEGF) signaling pathway.^50–52^ It remains unclear whether EYA4 or other EYA proteins regulates glycolysis. Here, we demonstrate that EYA4 inhibits the expression of glycolytic genes, glucose uptake, and the production of lactate, pyruvate and ATP in liver cancer cells and breast cancer cells, suggesting that EYA4 is a repressor of glycolysis.

Growth factors play a pivotal role in tumorigenesis and tumor progression. Growth factors can stimulate glycolytic gene expression, leading to increased glycolysis. For example, TGFβ increases expression of 6-phosphofructo-2-kinase/fructose-2,6-bisphosphatase 3 (PFKFB3), a gene involved in glycolysis, through activation of the p38 MAPK and phosphoinositide 3-kinase (PI3K)/Akt signaling pathways.^53^ According to reports that SIX1 activates TGFβ signaling pathway by stimulating expression of TGFβ and the Type I TGFβ receptor, both of which activate ERK.^54–57^ Iwanaga et al. suggested that SIX1 may modulate ERK signaling through TGFβ signaling and other signaling pathways.^58^ Combined with our observation that ERK increases SIX1 phosphorylation and expression, these findings propose that the SIX1/TGFβ/ERK and SIX1/ERK axes may form positive feedback loops. Since TGFβ activates ERK through multiple steps, TGFβ regulates glycolytic gene expression indirectly via the ERK/SIX1 axis. EGF-dependent ERK activation reportedly enhances expression of the glycolytic genes GLUT1 and LDHA and subsequent glycolysis, although the detailed mechanisms remain to be investigated.^59^ IGF1 increases glycolysis possibly through HIF1α, a key regulator of glycolysis, because IGF1 stimulates HIF1α expression.^10^ IGF1 induction of HIF-1α in cancer cells is blocked by treatment with inhibitors targeting the PI3K and MAPK pathway. VEGF stimulation caused increased HIF1α expression and glycolysis in pancreatic cancer.^60^ In this study, growth factors, such as TGFβ, EGF, IGF1, and VEGF, converge on the transcription factor SIX1 to stimulate glycolytic gene expression and glycolysis through ERK-dependent SIX1 phosphorylation. Thus, we identify a common mechanism underlying growth factors-mediated glycolysis. Because of the importance of growth factor signals in cancer development and progression, targeting growth factors/SIX1 pathway may be a new approach for cancer therapy.

Researchers usually turn to phosphate mimicking when authentic phosphorylated samples cannot be obtained. Aspartate (D) and glutamate (E), both of which are acidic amino acids, are commonly regarded as an effective phosphomimetic substitution for serine (S) or threonine (T).^61^ However, in some cases, mimics cannot replicate the properties of real phosphate groups. For instance, α-synuclein (S129D/E) did not imitate the structure and aggregation characteristics of truly phosphorylated α-synuclein.^62^ Protein kinase B/AKT (T308D/E) did not activate its substrates like authentically phosphorylated AKT.^63^ In our study, SIX1 (S225D/E) also failed to mimic really phosphorylated SIX1 in terms of regulation of glycolytic gene expression, production of lactate and ATP, cancer cell proliferation and invasion, and tumor growth and metastasis. Surprisingly, SIX1 (S225K) can simulate SIX1 to regulate the above-mentioned effects, and even has elevated activities than WT SIX1. Unlike D and E, K is a basic amino acid. However, the mutation of S at 225 of SIX1 to arginine (R) and histidine (H), two other basic amino acids, still cannot simulate the function of phosphorylated SIX1. Compared with WT SIX1, SIX1 (S225K) has enhanced binding capability to the promoters of its target genes, suggesting that SIX1 phosphorylation is required for its binding to target gene promoters. These data imply that both charge of amino acids and conformation, especially conformation, may be involved in SIX1 (S225K) binding to negatively charged DNA molecules. However, S225 is not in the SD domain or the HD domain of SIX1, and is rather in an unstructured region. We cannot exclude the possibility that the region around S225 may interact with other proteins important for DNA binding. The mechanisms how the lysine residue becomes an effective phosphomimetic substitution for serine remain to be investigated. Overall, our results provide a previously unidentified mode for non-classical phosphorylation mimics of a protein.

There are some limitations of our study. Although SIX1 is necessary for or mainly involved in some growth factors-mediated promotion of glycolytic gene expression and glycolysis, we do not know whether SIX1 is also important for cancer cell proliferation, invasion, and metastasis regulated by these growth factors. SIX1 phosphorylation controls tumor growth and metastasis. We also do not know whether SIX1 phosphorylation is critical for growth factor modulation of tumor growth and metastasis. In addition, we identify a non-canonical phosphorylation mimic for the first time. However, we do not know exactly how a basic amino acid, but not acidic amino acids, can mimic phosphorylation, an interesting issue for future investigation.

## Materials and methods

### Plasmids and reagents

Mammalian expression vectors, including untagged and FLAG- and MYC-tagged, were constructed by introducing PCR-amplified segments into pcDNA3 (Invitrogen). GST- or His-tagged prokaryotic expression plasmids were built with pGEX-KG (Amersham Pharmacia Biotech) and pET28a (Novagen). Mutants for the FLAG-, MYC- GST- or His-tagged proteins were generated by recombinant PCR. SIX1 mutants were constructed using the Mut Express II Fast Mutagenesis Kit (Vazyme). Lentiviral shRNA vectors were generated by inserting short hairpin RNA fragments into pSIH-H1-Puro (System Biosciences). The cDNA target sequences of siRNAs for ERK2 and EYA4 or shRNA for PKM2 were listed in Supplementary Table 1.

The pS225 antibody was made by Abmart. The designed peptide of SIX1, Ac-CFSPPQS(p)PDQ-NH2, was used as an antigen to raise polyclonal antibodies in rabbit. Anti-Myc (sc-40HRP), anti-β-actin (sc-47778HRP), anti-GFP (sc-9996HRP), anti-Lamin A/C (sc-376248), and anti-PFKL (sc-292523) were ordered from Santa Cruz Biotechnology; anti-FLAG (A8592), anti-HA (H6908), and anti-FLAG M2 agarose (A2220) was purchased from Sigma-Aldrich; anti-α-Tubulin (11224-1-AP), anti-SIX1 (10709-1-AP), anti-HK2 (22029-1-AP), anti-LDHA (19987-1-AP), anti-FZR1 (16368-1-AP), and anti-EYA4 (24691-1-AP) were purchased from Proteintech; anti-ERK1 (4372), anti-ERK2 (9108), anti-CDK1 (9116), anti-PKM2 (4053S), anti-ERK1/2 (4695), anti-phospho-ERK1/2 (pERK1/2) (4370), and anti-Ubiquitin (14049) were purchased from Cell Signaling Technology; anti-GST (RPN1236) and anti-His (27471001) were obtained from GE Healthcare Life sciences; ChIP grade anti-MYC antibody (ab9132) was from Abcam.

### Cell culture and transfection

Human breast ZR75-1 (CRL-1500) and liver HepG2 (HB-8065) cancer cell lines and embryonic kidney HEK293T cell line (CRL-11268) were purchased from American Type Culture Collection (ATCC). MHCC97-H cells (C6585) were purchased from Beyotime. Cells have previously been tested for mycoplasma contamination, and were routinely grown at 37 °C in a humid environment with 5% CO_2_ in DMEM (Macgene) with 10% FBS (Every Green), 25 mM glucose and 1% penicillin-streptomycin-amphotericin B solution (100×) (Solarbio). Plasmids and siRNAs were transfected using VigoFect (Vigorous) and Lipofectamine RNAiMAX (Invitrogen), respectively. Cells were harvested for further analysis 24 h after plasmid transfection or 48 h after siRNA transfection. To transfect siRNAs together with plasmids, cells were first transfected with siRNAs for 24 h and subsequently transfected with plasmids and harvested 24 h later. For stable transfections, transfected cells were selected in 500 μg/ml G418 (Invitrogen) for approximately 2 months. Lentiviruses were generated by co-transfecting HEK293T cells with recombinant lentiviral vectors and the pPACK Packaging Plasmid Mix (System Biosciences, Johnstown, PA) using Megatran reagent (Origene, Rockville, MD) as per the manufacturer’s instructions. Viral supernatants were collected 48 h post-transfection, and titers were determined. To establish stable cell lines, infected MHCC97-H cells were selected using 1 μg/mL puromycin. Pooled clones or individual clones were subsequently screened by immunoblotting and produced similar results. For analysis of growth factors/cytokines, cells were starved in serum-free DMEM for 24 h, followed by treatment with 50 ng/ml EGF, 100 ng/ml IGF1, 100 ng/ml TGFα, 5 ng/ml TGFβ, 10 ng/ml VEGF, 100 ng/ml IL-6, 20 ng/ml TNFα and 500 U/ml IFNγ for 12 h. For the galactose experiment, cells were grown in glucose-free DMEM supplemented with 10% FBS plus 10 mM galactose. Cells were then exposed to 2.5 mM 2-DG (Sigma-Aldrich) and 0.1 mM oligomycin (Sigma-Aldrich) at indicated times.

### *SIX1* knockout cancer cell lines

CRISPR/Cas9 system was used to establish the *SIX1* KO HepG2 and ZR75-1 cell lines. One guide RNA (sgRNA) sequence targeting SIX1 (CCTGCACAAGAACGAGAGCGTAC) was designed using the web CRISPR Design Tool (http://crispr.mit.edu), and inserted to the lentiCRISPR v2 vector (Addgene #52961). HEK293T cells were transfected with Lipofectamine 3000 (Thermo Fisher Scientific) carrying the sgRNA vector and package plasmids (PAX2 and VSVG). At 48 h post transfection, the supernatants were centrifuged at 1000 × *g* for 10 min and filtered through 0.45 μm membrane. HepG2 and ZR75-1 cells were infected with lentiviruses and 8 μg/ml polybrene (Sigma-Aldrich). Finally, stable clones were selected using 1 μg/ml puromycin for three weeks, and verified by DNA sequencing of genomic fragments and immunoblot. The CRISPR cell lines were clonal.

### Immunoblot

Protein lysates from cancer cells or tumor tissues were prepared on ice with RIPA lysis buffer (Sigma-Aldrich). After SDA-PAGE, proteins were transferred to nitrocellulose membranes. The membranes were blocked in 5% skimmed milk for 1 h, and incubated with primary antibodies at room temperature for 2 h or at 4 °C overnight. After washing, the membranes were further incubated for 1 h at room temperature with HRP-conjugated secondary antibodies, followed with three washes in TBST buffer. Lastly, the membranes were detected using enhanced ECL Chemiluminescent Assay Solution (Vazyme). The bands were detected by ChemiDoc Imaging Systems (Bio-Rad).

### Mass spectrometry (MS)

Approximately 1 ×10^8^ ZR75-1/FLAG-SIX1 cells were used for immunoprecipitation using an anti-FLAG antibody (Sigma-Aldrich) to enrich the FLAG-tagged SIX1 and its binding partners. Briefly, cells were lysed in IP buffer (20 mM Tris at pH 8.0, 0.25 M NaCl, 0.5% NP-40, 5 mM EDTA) and centrifuged to separate the supernatants. The supernatants were then incubated with anti-FLAG agarose beads for 4 h at 4 °C to perform immunoprecipitation. The beads were washed three times with IP buffer, and the FLAG-SIX1 bound complex was eluted using the FLAG peptide. The purified proteins were separated by gradient SDS–PAGE and analyzed with mass spectrometry (MS). For phosphorylation analysis, the purified SIX1 protein was harvested and digested, followed by TiO2 enrichment.^64^ In-solution and in-gel digestion were then performed.^65^ The samples were analyzed by nanoLC-MS/MS (nanoACQUITY UPLC and SYNAPT G2 HD mass spectrometer, Waters). MS data were acquired with Data Dependent Analysis mode, processed with PLGS 2.4 software (Waters), and the resulting peak list was searched against the NCBI database utilizing the MASCOT engine, de novo sequencing was performed using Masslynx Pepseq 4.1 software (Waters). The original results were shown in Dataset 1.

### In vitro kinase assay

The expression and purification of GST fused proteins were performed following the manufacturer’s instructions (Amersham Pharmacia). Briefly, GST-fusion recombinant vectors were transferred into *E.coli* BL21 cells to conduct protein purification. After inducing growth for overnight at 16 °C with 0.5 mM IPTG, the bacteria were harvested, resuspended in lysis buffer, and sonicated. After centrifugation, the supernatants were incubated with glutathione-Sepharose 4B resin (Amersham Pharmacia) in lysis buffer for 4 h at 4 °C. The beads were washed with lysis buffer for 3 times and collected. Recombinant ERK2 protein (0.5 μg) (Invitrogen) was mixed with purified GST alone, GST-SIX1 or its mutant (0.5 μg) in 1× kinase buffer (Cell Signaling Technology) containing γ-^32^P-ATP for 30 minutes at 37 °C. The reaction products were analyzed by SDS-PAGE and autoradiography.

### In vitro phosphatase assay

His fused proteins were expressed and purified following the protocols provided by QIAGEN. Briefly, His-tagged recombinant vectors were transferred into *E.coli* BL21 cells to perform protein purification. After inducing growth for overnight at 20 °C with 0.5 mM IPTG, the bacteria were collected, re-suspended in lysis buffer, sonicated and centrifuged. The supernatants were incubated with His-Sepharose beads (Amersham Pharmacia) for 4 h at 4 °C. The beads were harvested and washed 3 times with lysis buffer. Purified FLAG-SIX1 protein from HepG2 cells with anti-FLAG beads (Sigma-Aldrich) was used as a substrate. Purified His-tagged proteins (1 μg) were incubated with the substrate (1 μg) in phosphatase buffer (50 mM Tris-HCl, pH 7.0, 5 mM MgCl2, 10% glycerol, 3 mg/ml BSA) at 37 °C for 30 min. The reaction mixtures were analyzed with immunoblot using anti-pS225 antibody.

### Reverse transcription-quantitative PCR (RT-qPCR)

Total RNA was isolated using TRIzol reagent according to the manufacturer’s protocol (Tiangen). One microgram of RNA was reverse transcribed to cDNA using Evo M-MLV RT Premmix for qPCR (Accurate Biotechnology). Real-time PCR analysis was conducted using TB Green Premix (Takara Bio) and the CFX96 Real-Time PCR detection system (Bio-Rad). The expression of the target gene was normalized to β-actin and calculated using the 2^−ΔΔCt^ method. The primer sequences used for Real-time PCR are listed in Supplementary Table 2.

### Chromatin immunoprecipitation (ChIP)

The Magna ChIP G Chromatin Immunoprecipitation Kit (Millipore) was used to perform the ChIP assay in accordance with the guidelines provided by the manufacturer. Briefly, cells were cross-linked with 37% formaldehyde, pelleted, and resuspended in lysis buffer, followed by sonication and centrifugation. Normal IgG or anti-MYC antibody was added for overnight at 4 °C, and Protein G magnetic beads were subsequently co-incubated with protein-antibody complex for additional 2 h. The ChIP DNA fragments were eluted, purified, and quantified by qPCR and the primers were listed in Supplementary Table 3.

### Lactate and ATP assays

For lactate production assay, 2 ×10^4^ cells were plated in 96-well plates and incubated in an incubator for more than 10 h. The serum-free culture media were replaced and incubated at room temperature for 1 h. Lactate Assay Kit II was used to measure at 450 nm using a microplate reader and the lactate levels were normalized to the number of cells according to the manufacturer’s protocol (Biovision). For ATP level analysis, 1 ×10^6^ cells were lysed in ATP buffer on ice for 15 min. ATP levels were determined at 570 nm and the values were normalized to cell number according to ATP Colorimetric Assay kit’s protocol (Biovision). To assess the lactate and ATP levels in liver tumor tissues, 10 mg of fresh tissue was subjected to homogenization in the assay buffer (Biovision). Homogenates were cleared by centrifugation at 13,000 rpm, and the soluble fractions were collected for quantitation and normalization of protein concentrations.

### Cell proliferation assay

Approximately 3 ×10^3^ cells were plated in 96-well plates. After 72 h, CCK8 solution (Dojindo Laboratories) diluted 1:10 with media was added and incubated at 37 °C for 1 h in a humidified atmosphere with 5% CO2 according to the manufacturer’s protocol. The OD values were examined at 450 nm using a microplate reader.

### Cell invasion assay

Cells were trypsinized, washed with 1 ×PBS, and resuspended in DMEM without FBS. Forty thousand cells were seeded into the upper chamber covered with matrix (BD Biosciences), and the low chamber was filled with DMEM media with 20% FBS. After 24 h, the chamber was washed twice with PBS, and the invasive cells were fixed with 4% paraformaldehyde and stained with 0.5% crystal violet for 30 min. Cell invasion was observed and captured under a microscopy.

### Co-immunoprecipitation (Co-IP)

For transfection-based Co-IP assays, cells were transfected with the indicated plasmids using VigoFect (Vigorous Biotechnolgy), lysed in 500 μl of lysis buffer (50 mM Tris at pH 8.0, 500 mM NaCl, 0.5% NP-40, 1 mM dithiothreitol and protease inhibitor tablets), and subjected to immunoprecipitation with anti-FLAG M2 agarose beads (Sigma-aldrich) overnight at 4 °C or at room temperature for 2 h. The beads were washed and eluted with lysis buffer and SDS sample buffer, respectively. For detecting endogenous protein-protein interactions, cells were harvested, rinsed in pre-cold PBS, and lysed in lysis buffer. The protein extracts were then immunoprecipitated with control IgG (Santa Cruz Biotechnology) or indicated antibodies.

### Ubiquitination assay

Cells were treated with the proteasome inhibitor MG132 (10 μM) for 4 h. The cells were harvested, lysed in IP buffer (20 mM Tris at pH 8.0, 0.25 M NaCl, 0.5% NP-40, 5 mM EDTA), and centrifuged. The supernatants were immunoprecipitated with indicated antibodies. The immunocomplexes were subjected to immunoblot analysis.

### Immunofluorescence

Cells grown on glass coverslips were fixed with 4% paraformaldehyde. After washing three times with PBS, cells were permeabilized in 0.5% Triton-100 and blocked in 1% goat sera. Cells were initially incubated with anti-MYC at 4 °C overnight, followed by incubation with secondary antibody at room temperature for 1 h. Nuclei were counterstained with DAPI prior to imaging with microscope.

### Nuclear and cytoplasmic protein extraction

Cellular fractionation assay was conducted with Nuclear and Cytoplasmic Protein Extraction Kit (Beyotime). Briefly, 20 µL of cell precipitates were added to 200 µL of PMSF-spiked Cytoplasmic Protein Extraction Reagent A. After centrifugation, the supernatant (cytoplasmic proteins) was collected. Nuclear proteins were obtained by adding Nucleoprotein Extraction Reagent to the cell precipitates.

### Animal experiments

Animal protocols were approved by the Institutional Animal Care Committee of Beijing Institute of Biotechnology (ethical approval number: IACUC-DWZX-2020-628). All mice were housed in a temperature-controlled and humidity-controlled facility with free access to food and water, following a 12-h dark/light cycle. Targeted conditional SIX1 (S225K) knockin (KI) C57BL/6 mice were generated by Cyagen Biosciences Inc. Briefly, the gRNA to mouse *SIX1* gene, the donor vector containing “loxP-KI-3’UTR of mouse *SIX1*-3*SV40 pA-loxP-KI region-p.S225K(AGT to AAG)” cassette, and Cas9 mRNA were co-injected into fertilized mouse eggs to generate targeted conditional knock in offspring. F0 founder animals (*S225K*^*fl/+*^) were identified by PCR followed by sequence analysis. *S225K*^*fl/+*^ mice were crossed to obtain *S225K*^*fl/fl*^ mice. *S225K*^*fl/fl*^ mice were bred with hepatocyte-specific Albumin-Cre recombinase (Alb-Cre) transgenic mice to generate liver-specific SIX1 (S225K) knock-in mice (Alb-Cre;*S225K*^*fl/fl*^). *S225K*^*fl/fl*^ mice and Alb-Cre mice were identified by PCR with expected products of 194 bp and 390 bp, respectively. Therefore, PCR products of both 194 bp and 390 bp were observed in Alb-Cre;*S225K*^*fl/fl*^ mice, and only 194 bp PCR product was observed in *S225K*^*fl/fl*^ mice. The genotyping PCR primers were as follows: Alb-Cre forward primer, 5’- GAAGCAGAAGCTTAGGAAGATGG-3’; Alb-Cre reverse primer, 5’- TTGGCCCCTTACCATAACTG-3’; *S225K*^*fl/fl*^ forward primer, 5’- AGCAGTCCTGAAGTTGAGTGG-3’; *S225K*^*fl/fl*^ reverse primer, 5’-CCTCCCATCTTCTAGC-3’. To induce liver tumorigenesis, mice were injected intraperitoneally at 2 weeks of age with 25 mg/kg DEN (Sigma-Aldrich) and were euthanized at the indicated time.

For xenograft tumor growth and metastasis assays, 6-week-old male nude mice were purchased from Vital River Inc. Briefly, 1 × 10^7^ HepG2 cells or 5 × 10^6^ MHCC97-H cells carrying different constructs in 100 μL PBS were subcutaneously injected into the flank of mice. Mice were intraperitoneally administered with 2-DG (500 mg/kg) every other day. The volume of tumor was monitored using a caliper at indicated times and calculated as follows: Volume = (longest diameter × shortest diameter2)/2. For lung metastasis model, 1 × 10^6^ *SIX1* KO MHCC97-H cells stably transfected with FLAG-SIX1 WT or its mutants were injected into the lateral tail veins of mice. Lung metastasis was assessed after 35 days. Lung tissues were collected and used for hematoxylin and eosin (H&E) staining.

### RNA-sequencing (RNA-seq)

Total RNA was isolated using TRIzol (Tiangen) according to the manufacturer’s instructions. Library preparation and sequencing were carried out using Illumina® NovaSeq 6000 platform (Novogene, Tianjin, China). RNA libraries were prepared using NEBNext® Ultra™ RNA Library Prep Kit for Illumina® following manufacturer’s protocols. Raw data (raw reads) of fastp format were firstly processed through in-house perl scripts. In this step, clean data (clean reads) were obtained by removing reads containing adapter and reads containing ploy-N and low quality reads from raw data. At the same time, Q20, Q30 and GC content of the clean data were calculated. All downstream analyses were based on the clean data with high quality. Reference genome and gene model annotation files were downloaded from genome website directly. Index of the reference genome was built using Hisat2 v2.0.5 and paired-end clean reads were aligned to the reference genome using Hisat2 v2.0.5.

### Clinical samples and immunohistochemistry (IHC)

One hundred and two male liver cancer specimens with 29–79 years of age (mean age: 56 years) were obtained from Chinese People’s Liberation Army (PLA) General Hospital, and informed consent was obtained from the patients and approved by the Institutional Review Board of the PLA General Hospital (ethical approval number: S2016-098-01). The follow-up time for this cohort was 8–78 months (mean: 50.9 months). Immunohistochemistry (IHC) of formalin-fixed paraffin-embedded samples was performed as described previously.^66^ Briefly, the slides were subjected to deparaffinization, rehydration, and treatment with 3% hydrogen peroxide for 20 min. Antigens were retrieved with EDTA (pH = 8.0) by microwave for 3 min, and blocked with 10% goat serum to avoid nonspecific binding. Anti-ERK1/2, anti-EYA4, anti-HK2, anti-PKM2, anti-LDHA and anti-pS225 were incubated at dilutions of 1:200. The ERK1/2, EYA4 or pSIX1 score was calculated by multiplying the staining intensity (negative = 0; weak = 1; intermediate = 2; strong = 3) by percentage of immunoreactive cells (0–100%). Thus, the score is between 0–3. The optimal cutoff values of the IHC scores were determined using receiver operating characteristic (ROC) curve analysis. We defined score ≤0.85 as low pS225.

### Statistical analysis

Statistical calculations were performed using GraphPad Prism 9. Student’s *t* test was used to compare the means between two groups. One-way ANOVA was used for multiple comparisons. The correlation of pS225 expression with ERK1/2 and EYA4 expression were determined using Pearson’s correlation test. Disease-free survival and overall survival curves were generated by the Kaplan‒Meier method and differences between survival curves were determined using the log‒rank test. All statistical tests were two-sided. In all assays, *P* value of less than 0.05 was considered statistically significant.

## Supplementary information

Supplementary Material

Dataset 2

Dataset 1

## Data Availability

Experimental data supporting the conclusions of this study are available within the Article and the Supplementary Information. The RNA-Seq data has been deposited under the Gene Expression Omnibus (www.ncbi.nlm.nih.gov/geo/, accession number GSE254929). All other data supporting the findings of this study are available from the corresponding author upon reasonable request.
