# Supplementary material for: Phosphorylation determines the glucose metabolism reprogramming and tumor-promoting activity of sine oculis homeobox 1
Source: Signal Transduct Target Ther. 2024 Dec 2;9:337. doi: 10.1038/s41392-024-02034-5 (PMC11609306; doi:10.1038/s41392-024-02034-5)
Supplement: Supplementary file 1 — Supplementary Material [file 41392_2024_2034_MOESM1_ESM.docx]

Supplementary Materials for

Phosphorylation determines the glucose metabolism reprogramming and tumor-promoting activity of sine oculis homeobox 1

Yanni Lin^*^, Ling Li^*^, Bin Yuan^*^, Fei Luo^*^, Xiujuan Zhang^*^, Yuanjun Yang, Shaliu Luo, Jing Lin, Tianxing Ye, Youzhi Zhang, Shan Gao and Qinong Ye^#^.

Correspondence to: Qinong Ye (yeqn88@163.com)

**This PDF file includes:**

Figures S1 to S9

Tables S1 to S3


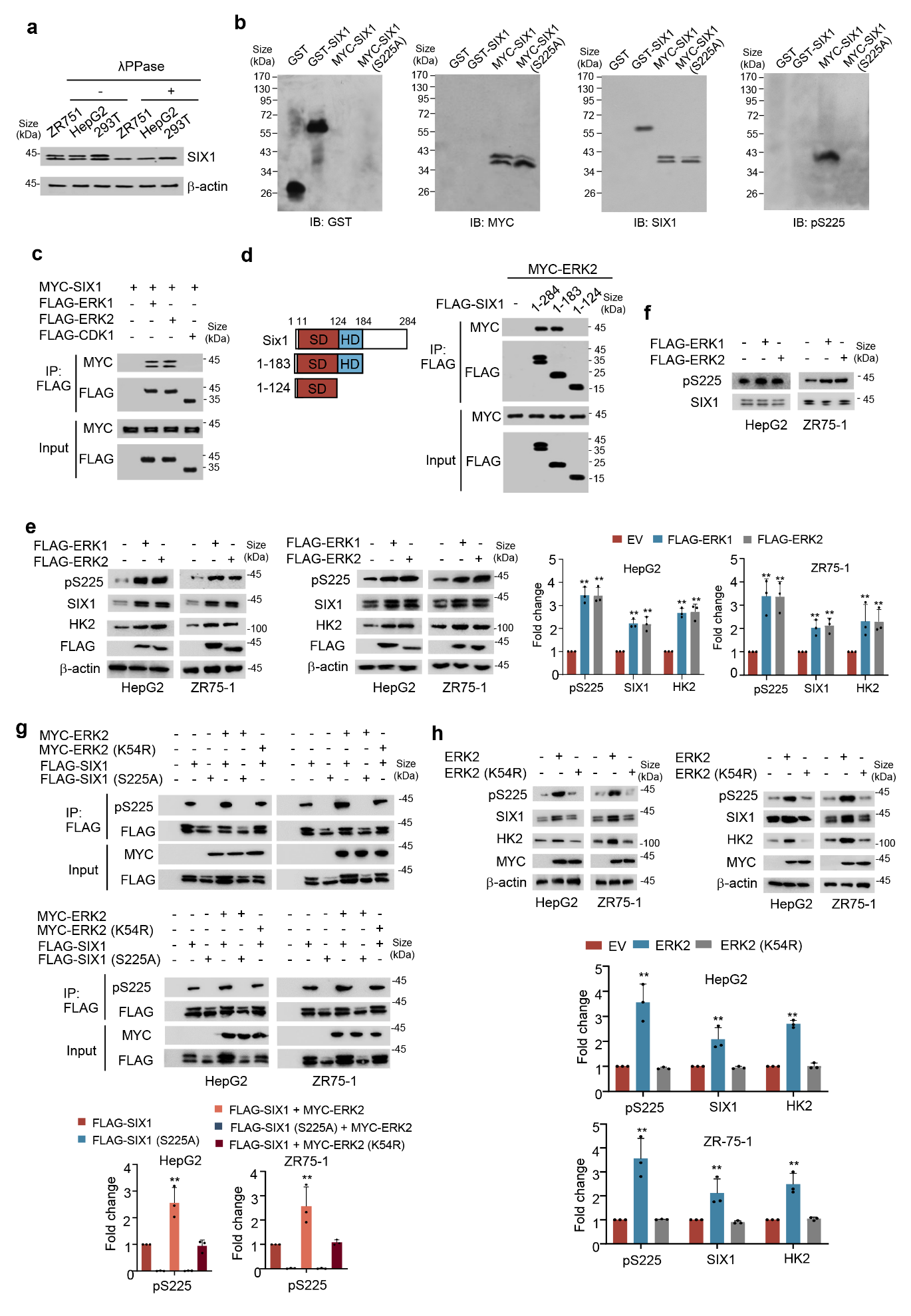


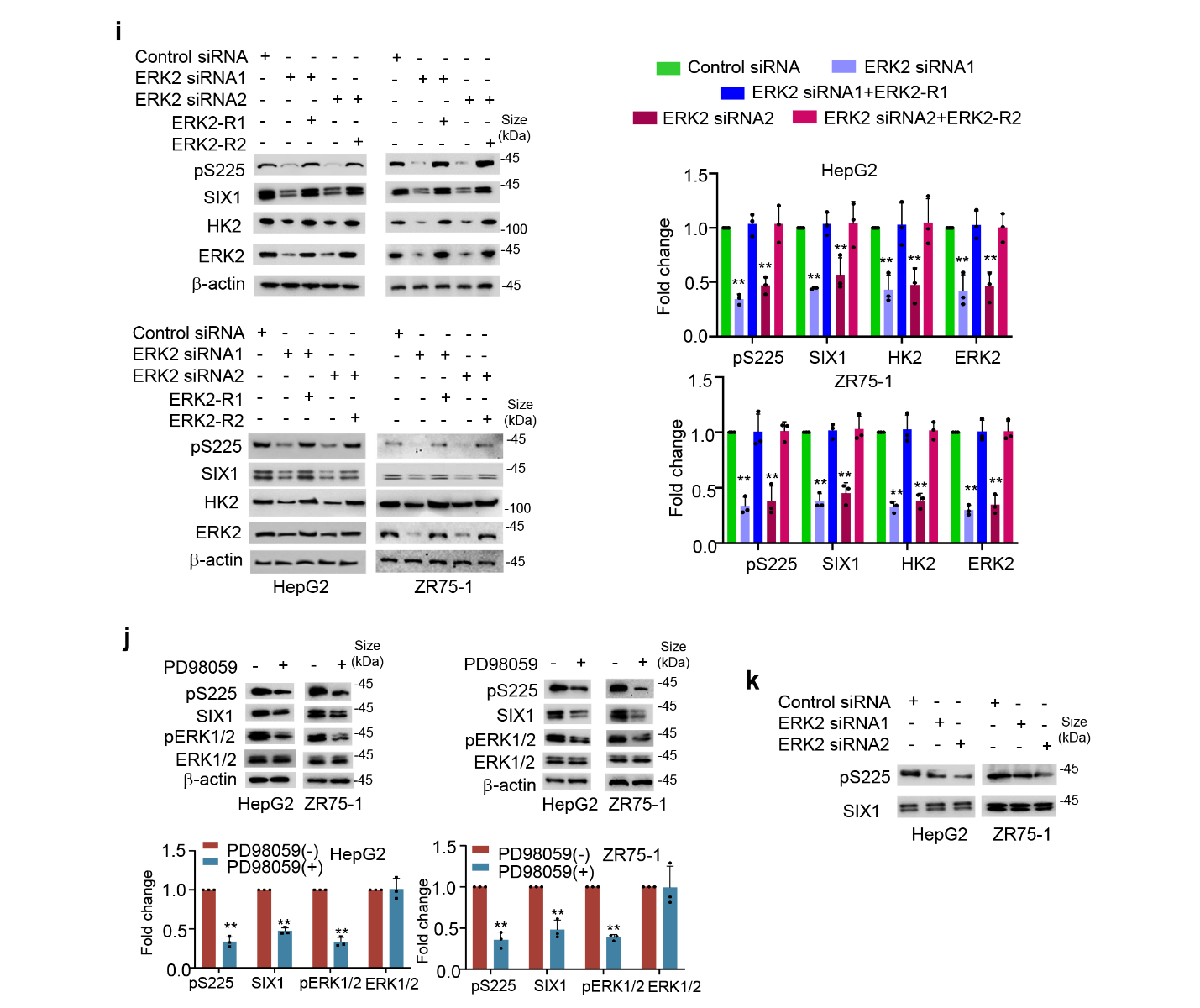


**Fig. S1. SIX1 is phosphorylated at S225 and interacts with ERK.** **a,** Immunoblot analysis of ZR75-1, HepG2 and HEK293T cells treated with or without λ protein phosphatase (λ-PPase). **b,** Immunoblot analysis of purified GST or GST-SIX1 from *E.coli* and purified immunoprecipitates with anti-MYC from ZR75-1 cells transfected with MYC-tagged SIX1 or SIX1 (S225A). The GST-SIX1 is not supposed to be phosphorylated, whereas the purified MYC-SIX1 is supposed to be phosphorylated. **c,** HEK293T cells were transfected with MYC-tagged SIX1 and FLAG-tagged ERK1/2 or CDK1. Cell lysates were immunoprecipitated with anti-FLAG, followed by immunoblot with the indicated antibodies. IP, immunoprecipitation. **d,** HEK293T cells were transfected with MYC-tagged ERK2 and FLAG-tagged SIX1 or its deletion mutants as indicated. Cells were analyzed as in c. Schematic diagrams of SIX1 protein and its mutants are shown on the left. SD, the SIX1 domain. HD, the homeobox domain. **e,** Two other experiments showing immunoblot analysis of HepG2 and ZR75-1 cells transfected with FLAG-tagged ERK1/2. β-actin was used as a loading control. The densitometric quantitation of the target proteins normalized to respective β-actin is shown at right. Values shown are mean ± SD of 3 independent experiments (Fig. S1e and Fig. 1e). **f,** Immunoblot analysis of HepG2 and ZR75-1 cells from Fig. 1e. Total SIX1 protein was used as a loading control. ***P* < 0.01 versus respective empty vector (EV). **g,** Two other experiments showing Co-IP analysis of HepG2 or ZR75-1 cells transfected with MYC-tagged ERK2 or ERK2 (K54R) and FLAG-tagged SIX1 or SIX1 (S225A) as indicated. Cell lysates were immunoprecipitated with anti-FLAG, followed by immunoblot with the indicated antibodies. The densitometric quantitation of relative binding normalized to respective input is shown at bottom. Values shown are mean ± SD of 3 independent experiments (Fig. S1g and Fig. 1f). ***P* < 0.01 versus respective FLAG-SIX1. **h,** Two other experiments showing immunoblot analysis of HepG2 and ZR75-1 cells transfected with MYC-tagged ERK2 or ERK2 (K54R). The densitometric quantitation of the target proteins normalized to respective β-actin is shown at bottom. Values shown are mean ± SD of 3 independent experiments (Fig. S1h and Fig. 1g). ***P* < 0.01 versus respective EV. **i,** Two other experiments showing immunoblot analysis of HepG2 and ZR75-1 cells transfected with control siRNA, ERK2 siRNA1/2 or ERK2 siRNA1/2 plus siRNA-resistant ERK1/2 (ERK2-R1/2). The densitometric quantitation of the target proteins normalized to respective β-actin is shown at right. Values shown are mean ± SD of 3 independent experiments (Fig. S1i and Fig. 1i). ***P* < 0.01 versus respective control siRNA. **j,** Two other experiments showing immunoblot analysis of HepG2 and ZR75-1 cells treated with 20 μm PD98059. The densitometric quantitation of the target proteins normalized to respective β-actin is shown at bottom. Values shown are mean ± SD of 3 independent experiments (Fig. S1j and Fig. 1j). **k,** Immunoblot analysis of HepG2 and ZR75-1 cells from Fig. 1i. Total SIX1 protein was used as a loading control. ***P* < 0.01 versus respective negative control.


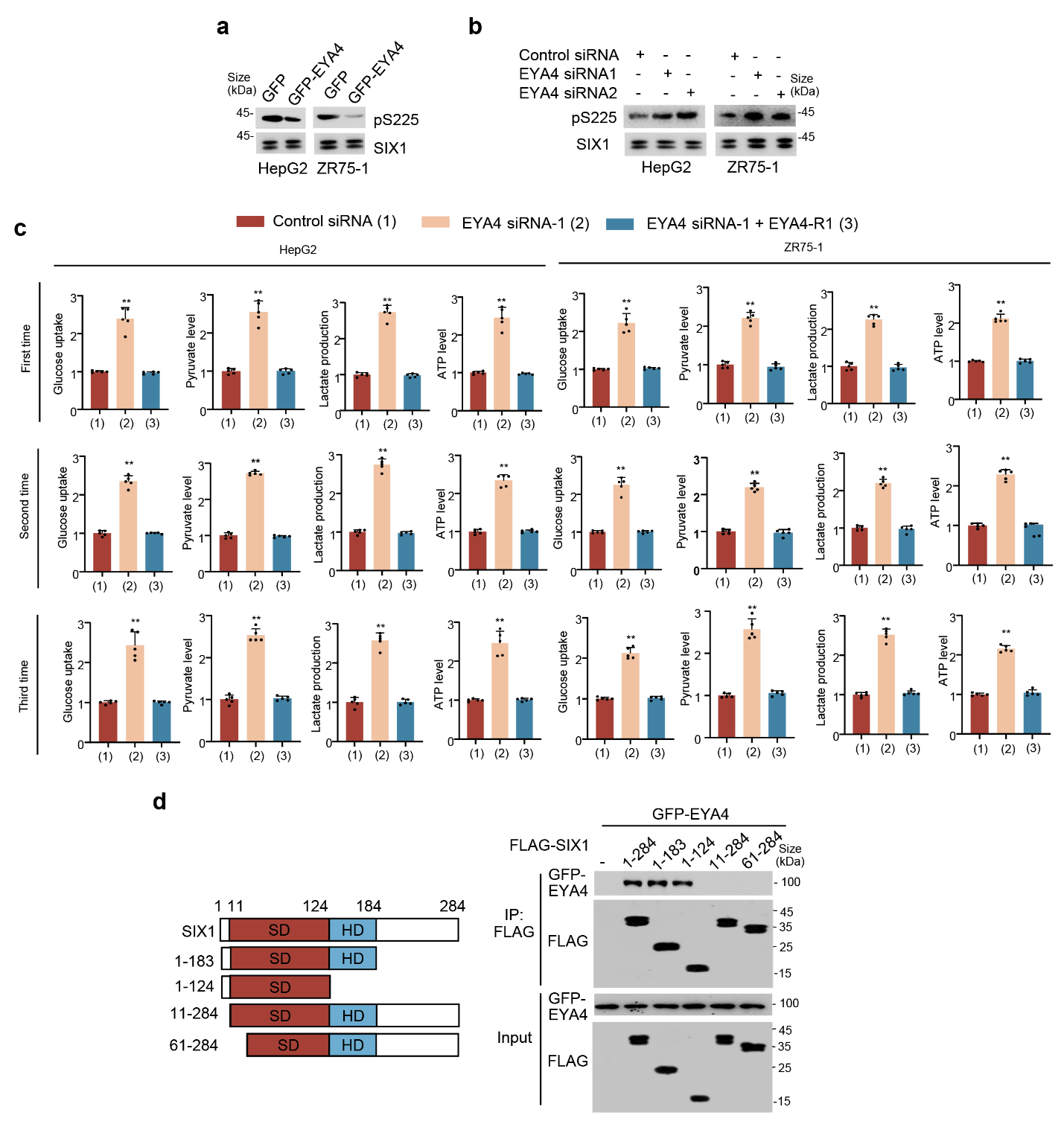


**Fig. S2. EYA4 inhibits glycolysis and interacts with N-terminal SIX1. a,** Immunoblot analysis of HepG2 and ZR75-1 cells from Fig. 2a. Total SIX1 protein was used as a loading control. **b,** Immunoblot analysis of HepG2 and ZR75-1 cells from Fig. 2b. Total SIX1 protein was used as a loading control. **c,** Three independent experiments showing analysis of glucose uptake and production of pyruvate, lactate and ATP in cells transfected with control siRNA, EYA4 siRNA1 or EYA4 siRNA1 plus siRNA-resistant EYA4 (EYA4-R1) as in Fig. 2b. Data shown are means ± SD of quintuplicate measurements that have been repeated three times with similar results. Statistical significance was assessed by two-tailed Student’s *t* test. ***P* < 0.01 versus HepG2 or ZR75-1 cells transfected with siCtrl. **d,** HEK293T cells were transfected with GFP-tagged EYA4 and FLAG-tagged SIX1 or its deletion mutants as indicated. Cell lysates were immunoprecipitated with anti-FLAG, followed by immunoblot with the indicated antibodies. Schematic diagrams of SIX1 protein and its mutants are shown on the left.


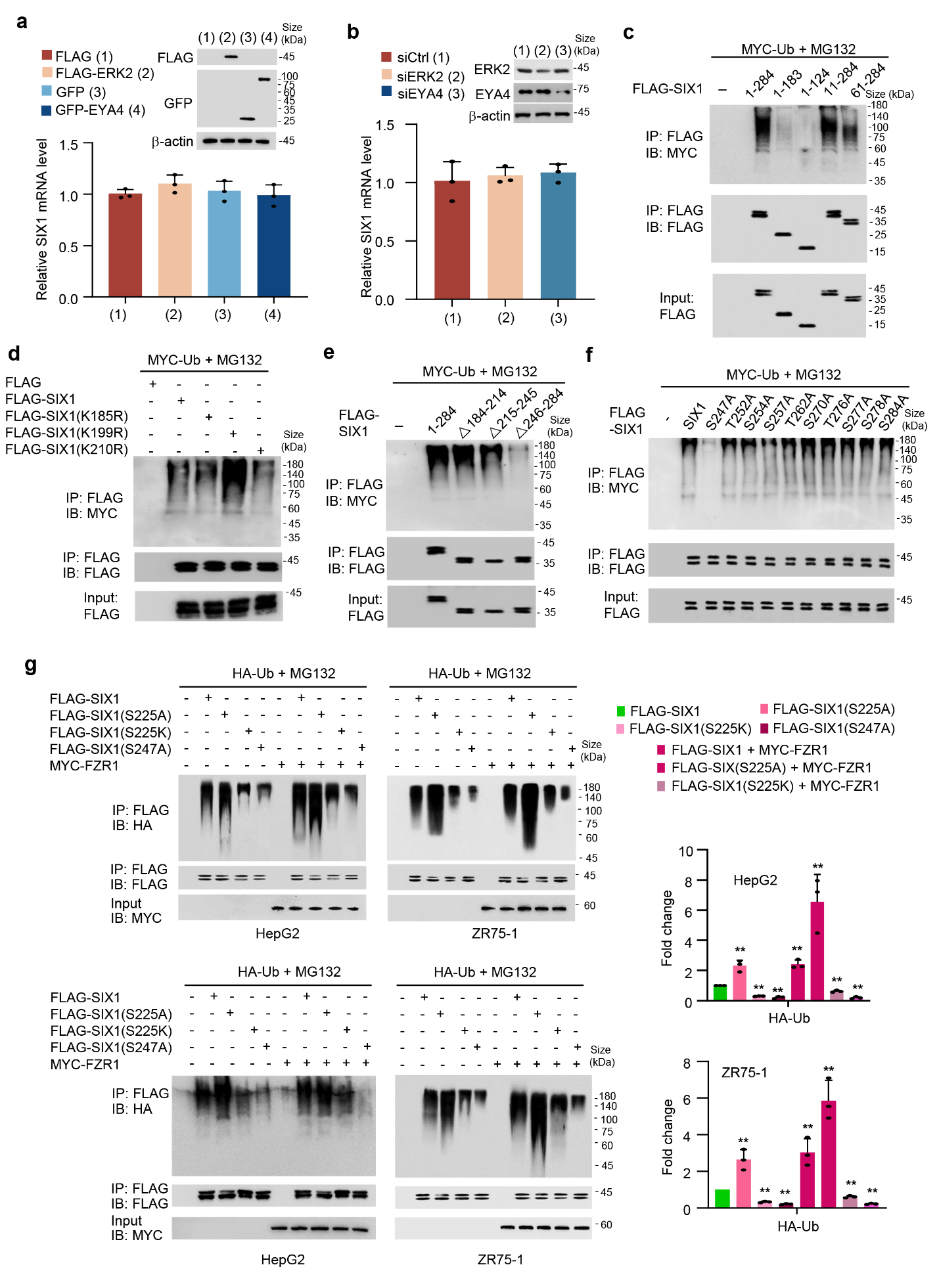


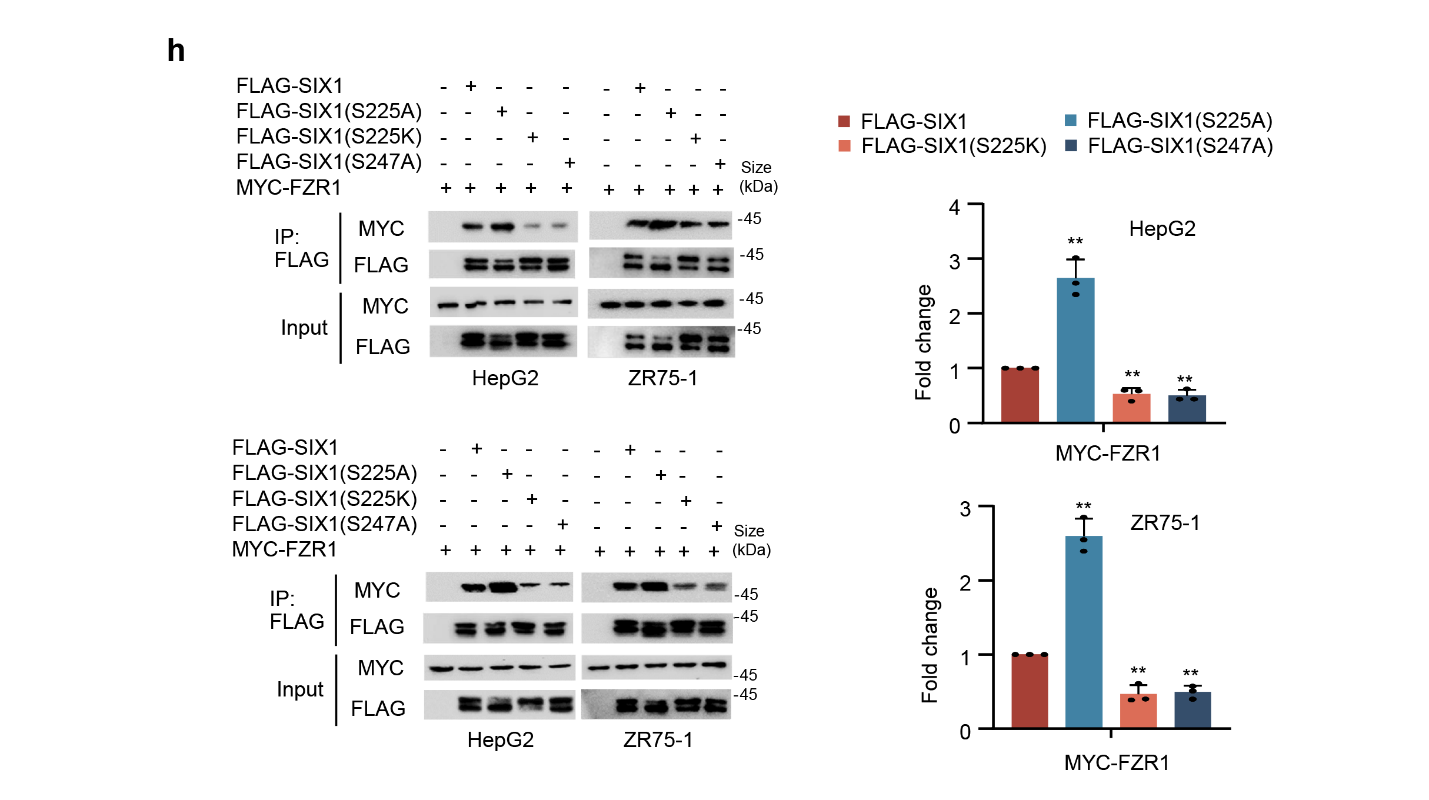


**Fig. S3. SIX1 phosphorylation controls its degradation via the ubiquitin-proteasome pathway. a,** RT-qPCR analysis of HepG2 cells transfected with FLAG-ERK2 or GFP-EYA4 as indicated. Immunoblot shows the expression of FLAG-ERK2, GFP and GFP-EYA4. **b,** RT-qPCR analysis of HepG2 cells transfected with siCtrl, siERK2 or siEYA4. Immunoblot indicates the expression of ERK2 and EYA4. Data shown are means ± SD of three independent experiments. Statistical significance was assessed by two-tailed Student’s *t* test. **c,** Ubiquitination analysis of HepG2 cells transfected with MYC-Ub and FLAG-tagged SIX1 or its deletion mutants and treated with 10 μM MG-132 for 4 h. Ub, ubiquitin. **d,** Ubiquitination analysis of HepG2 cells transfected with MYC-Ub and FLAG-tagged SIX1, SIX1 (K185R), SIX1 (K199R) or SIX1 (K210R) and treated with 10 μM MG-132 for 4 h. **e,** Ubiquitination analysis of HepG2 cells transfected with MYC-Ub and FLAG-tagged SIX1 (1-284), SIX1 (△184-214), SIX1 (△215-245) or SIX1 (△246-284) and treated with 10 μM MG-132 for 4 h. **f,** Ubiquitination analysis of HepG2 cells transfected with MYC-Ub and FLAG-tagged SIX1 or its point mutants and treated with 10 μM MG-132 for 4 h. **g,** Two other experiments showing ubiquitination analysis of HepG2 and ZR75-1 cells transfected with HA-Ub and FLAG-tagged SIX1 or its mutants with or without MYC-tagged FZR1 and treated with 10 μM MG-132 for 4 h. The densitometric quantitation of relative binding normalized to respective IP: FLAG and IB: FLAG is shown at right. Values shown are mean ± SD of 3 independent experiments (fig. S3g and fig. 3e). ***P* < 0.01 versus respective FLAG-SIX1. **h,** Two other experiments showing Co-IP analysis of HepG2 and ZR75-1 cells transfected with MYC-tagged FZR1 and FLAG-tagged SIX1 or its mutants. The densitometric quantitation of relative binding normalized to respective input is shown at right. Values shown are mean ± SD of 3 independent experiments (fig. S3h and fig. 3f). ***P* < 0.01 versus respective FLAG-SIX1.


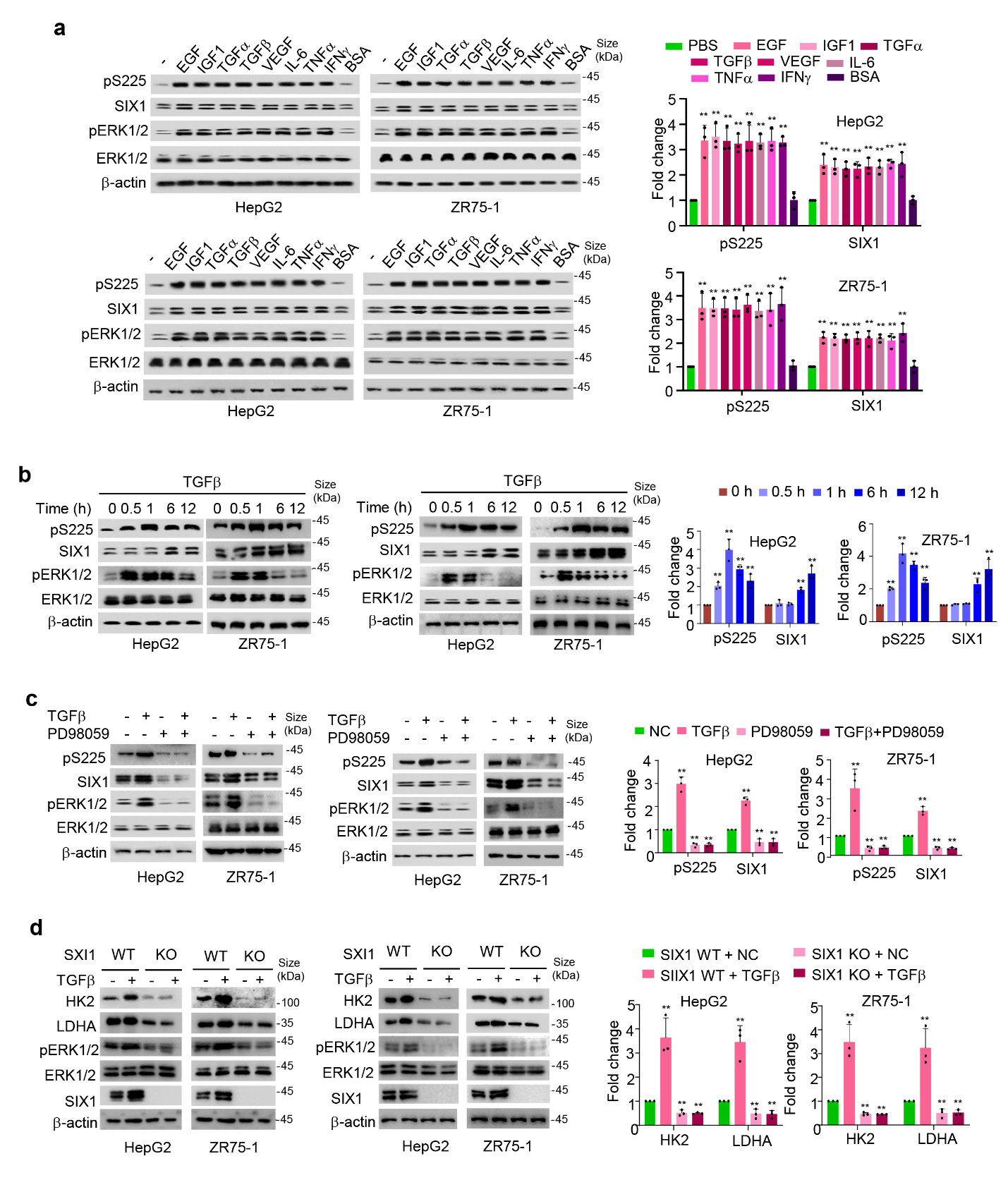

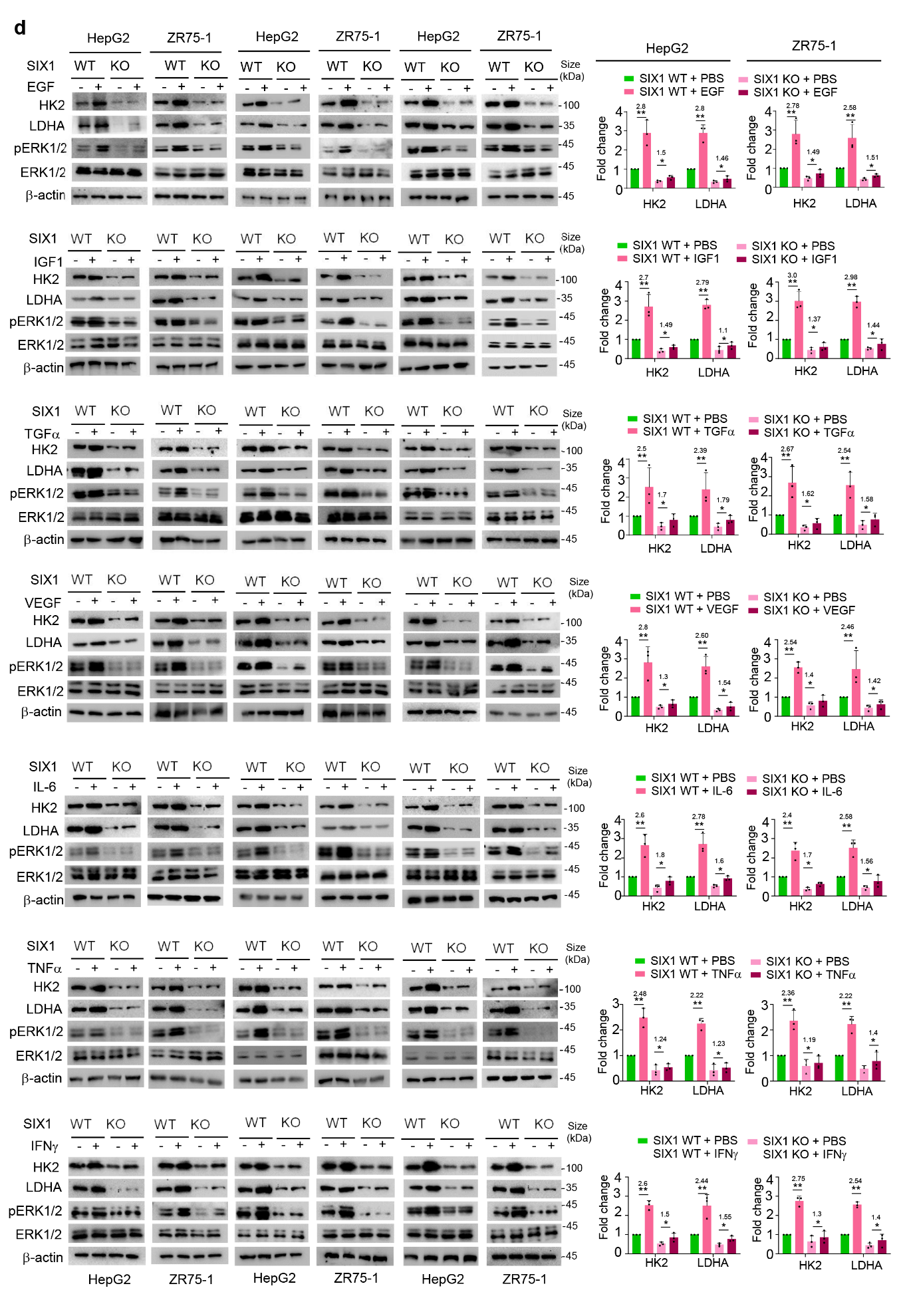

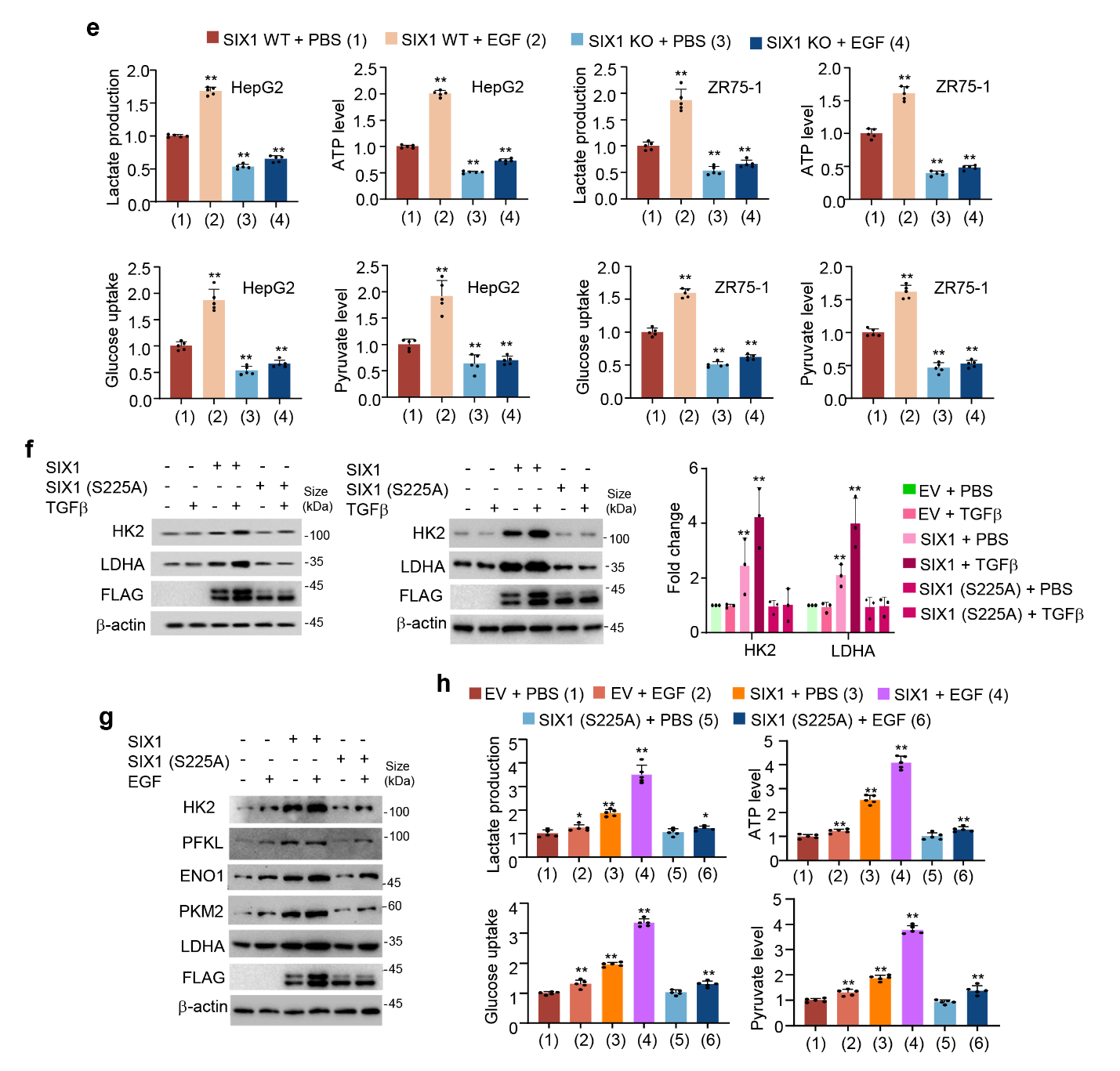


**Fig. S4. SIX1 is phosphorylated in response to growth factors and is responsible for EGF-mediated glycolysis. a,** Two other experiments showing immunoblot analysis of HepG2 and ZR75-1 cells treated with 50 ng/ml EGF, 100 ng/ml IGF1, 100 ng/ml TGFα, 5 ng/ml TGFβ, 10 ng/ml VEGF, 100 ng/ml IL-6, 20 ng/ml TNFα and 500 U/ml IFNγ for 12 h with BSA as a control. BSA, bovine serum albumin. The densitometric quantitation of the target proteins normalized to respective β-actin is shown at right. Values shown are mean ± SD of 3 independent experiments (Fig. S4a and Fig. 4a). ***P* < 0.01 versus respective PBS. **b,** Two other experiments showing immunoblot analysis of HepG2 and ZR75-1 cells treated with 5 ng/ml TGFβ for the indicated times. The densitometric quantitation of the target proteins normalized to respective β-actin is shown at right. Values shown are mean ± SD of 3 independent experiments (Fig. S4b and Fig. 4b). ***P* < 0.01 versus respective o h. **c,** Two other experiments showing immunoblot analysis of HepG2 and ZR75-1 cells pretreated with 20 μm PD98059 and then treated with 5 ng/ml TGFβ for 12 h. The densitometric quantitation of the target proteins normalized to respective β-actin is shown at right. Values shown are mean ± SD of 3 independent experiments (Fig. S4c and Fig. 4c). ***P* < 0.01 versus respective negative control (NC). **d,** Two to three independent experiments showing immunoblot analysis of SIX1 WT or KO HepG2 or ZR75-1 cells treated with 5 ng/ml TGFβ, 50 ng/ml EGF, 100 ng/ml IGF1, 100 ng/ml TGFα, 10 ng/ml VEGF, 100 ng/ml IL-6, 20 ng/ml TNFα or 500 U/ml IFNγ for 12 h. The densitometric quantitation of the target proteins normalized to respective β-actin is shown at right. Values shown are mean ± SD of 3 independent experiments (Figs. S4d and Fig. 4d). **P* < 0.05, ***P* < 0.01 versus respective SIX1 WT plus PBS. **e,** Analysis of glucose uptake and production of pyruvate, lactate and ATP in SIX1 WT or KO HepG2 or ZR75-1 cells treated with 50 ng/ml EGF as in d. Data shown are means ± SD of quintuplicate measurements that have been repeated three times with similar results. ***P* < 0.01 versus SIX1 WT plus PBS. **f,** Two other experiments showing immunoblot analysis of SIX1 KO HepG2 cells transfected with EV, FLAG-SIX1 or FLAG-SIX1 (S225A) and treated with or without 5 ng/ml TGFβ for 12 h. The densitometric quantitation of the target proteins normalized to respective β-actin is shown at right. Values shown are mean ± SD of 3 independent experiments (Figs. S4f and Fig. 4f). ***P* < 0.01 versus EV plus PBS. **g,** Immunoblot analysis of SIX1 KO HepG2 cells transfected with empty vector, FLAG-SIX1 or FLAG-SIX1 (S225A) and treated with or without 50 ng/ml EGF for 12 h. h, Analysis of glucose uptake and production of pyruvate, lactate and ATP in cells from g. Data shown are means ± SD of quintuplicate measurements that have been repeated three times with similar results. **P* < 0.05, ***P* < 0.01 versus EV plus PBS.


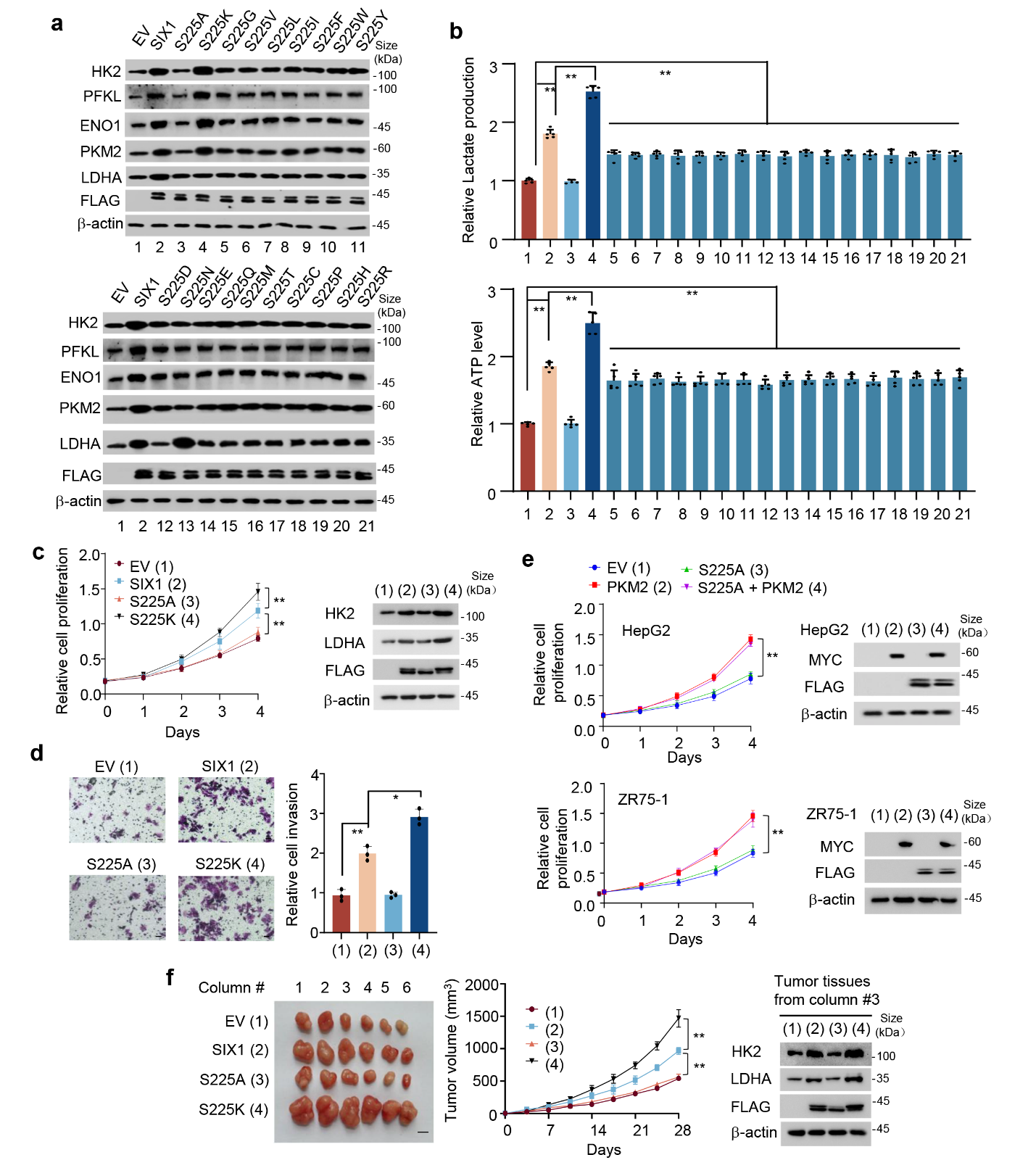


**Fig. S5. Non-canonical phosphorylation-mimetic SIX1 mutant promotes glycolysis and liver tumor growth. a,** Immunoblot analysis of SIX1 KO ZR75-1 cells transfected with FLAG-SIX1 or its mutants. **b,** Analysis of lactate production and ATP level in cells from a. Data shown are means ± SD of quintuplicate measurements that have been repeated three times with similar results. Statistical significance was assessed by one-way ANOVA test. ***P* < 0.01. **c,** The proliferation curve of SIX1 KO ZR75-1 cells stably transfected with FLAG-SIX1 or its mutants. Immunoblot shows the expression of HK2, LDHA and FLAG-SIX1 or its mutants. Data shown are means ± SD of three independent experiments. Statistical significance was assessed by one-way ANOVA test. ***P* < 0.01. **d,** Cell invasion assay of SIX1 KO ZR75-1 cells stably transfected as in c. The relative cell invasions are shown. Scale bar, 100 μm. Data shown are means ± SD of three independent experiments. Statistical significance was assessed by one-way ANOVA test. **P* < 0.05, ***P* < 0.01. **e,** The proliferation curve of SIX1 KO HepG2 or ZR75-1 cells transfected with FLAG-tagged SIX1 (S225A) and MYC-tagged PKM2. Cells were analyzed as in (c). **f,** The growth curve of xenograft tumors derived from SIX1 KO ZR75-1 cells stably transfected as in c. Scale bar,10mm. Representative immunoblot with the indicated antibodies was shown in the representative excised tumors. Statistical significance was assessed by one-way ANOVA test. ***P* < 0.01 at day 28.


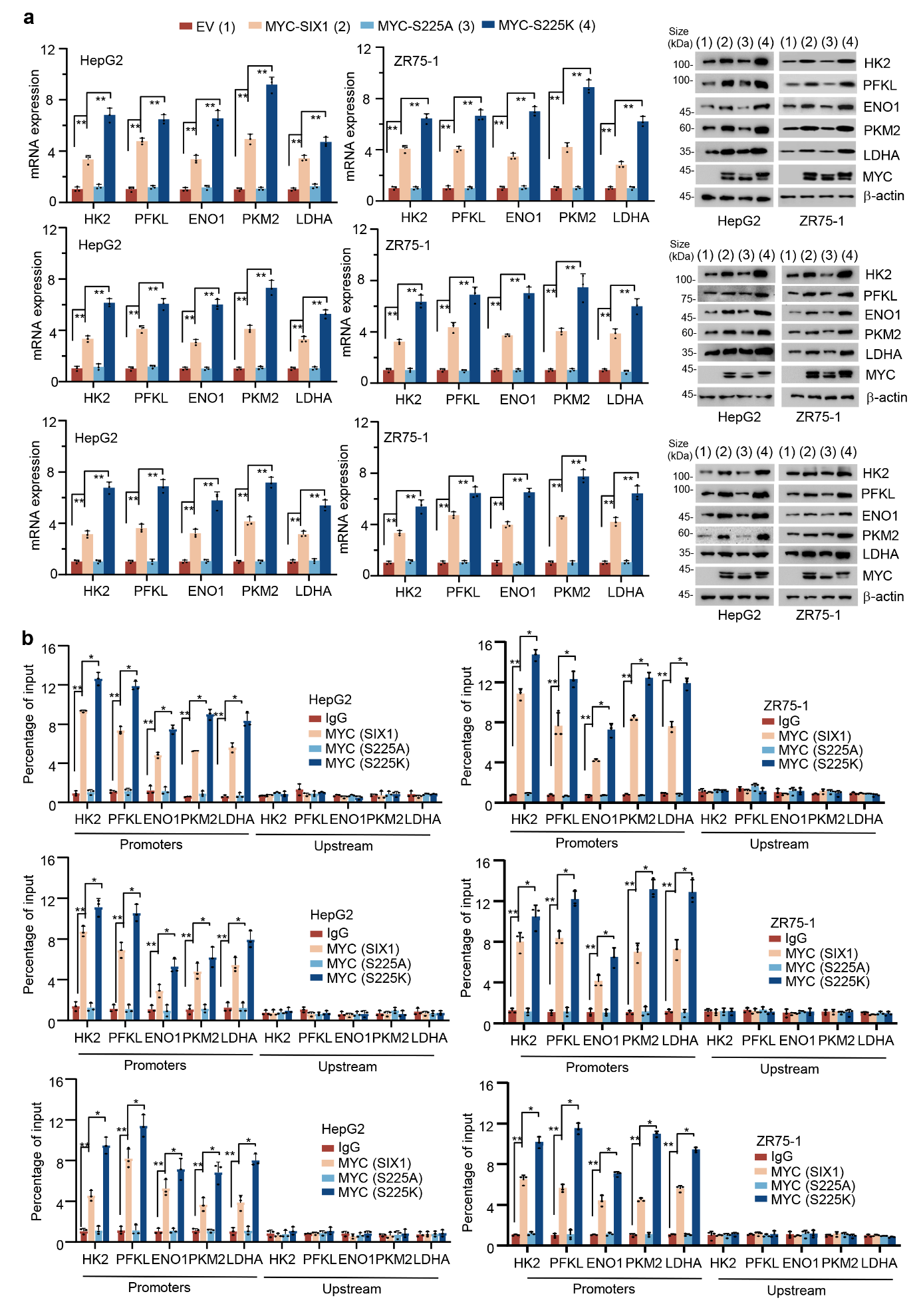


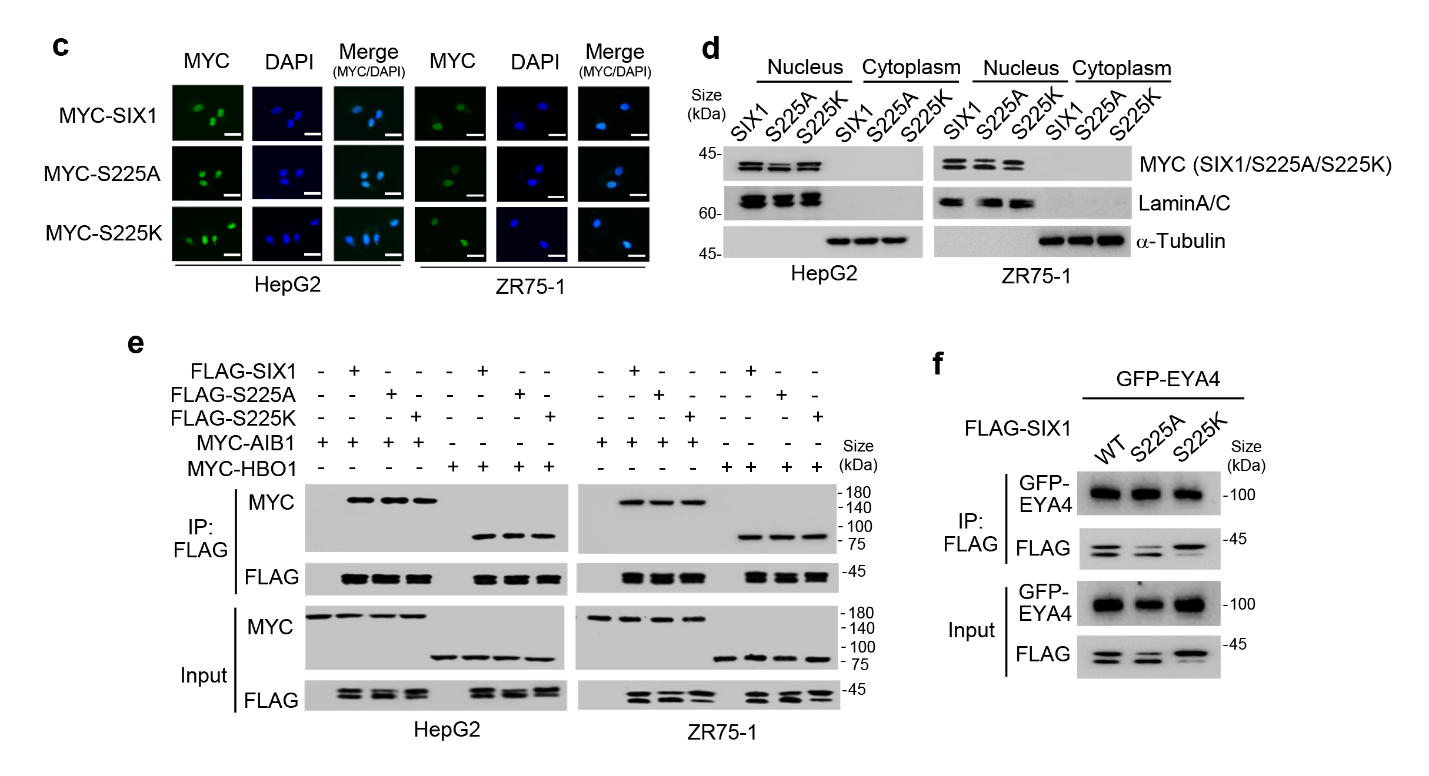


**Fig. S6. Non-canonical phosphorylation-mimetic SIX1 mutant enhances its binding to the downstream target gene promoters and transcription of glycolytic genes. a,** Three-independent experiments showing RT-qPCR and immunoblot analysis of SIX1 KO HepG2 or ZR75-1 cells stably transfected with MYC-tagged SIX1, SIX1 (S225A) or SIX1 (S225K). **b,** Three-independent experiments showing ChIP analysis of the occupancy of WT SIX1, SIX1 (S225A) or SIX1 (S225K) on HK2, PKM2 and LDHA promoters in SIX1 KO HepG2 or ZR75-1 cells stably transfected as in (a). Anti-MYC was used for ChIP. IgG, normal serum. Data shown are means ± SD of triplicate measurements that have been repeated 3 times with similar results (a, b). Statistical significance was assessed by two-tailed Student’s *t* test. **P* < 0.05, ***P* < 0.01. **c,** Immunofluorescence analysis of localization of SIX1, SIX1 (S225A) and SIX1 (S225K) in SIX1 KO HepG2 or ZR75-1 cells stably transfected as in a. Cells were stained with anti-MYC (green). The nuclei were stained with 4’,6’-diamidino-2-phenylindole (DAPI) (blue). Scale bars, 100 μm. **d,** SIX1 KO HepG2 or ZR75-1 cells stably transfected as in a were fractionated into nuclear and cytoplasmic fractions, and analyzed by immunoblot with anti-MYC for MYC-tagged SIX1, SIX1 (S225A) and SIX1 (S225K). Lamin A/C and α-Tubulin were used as the nuclear and cytoplasmic marker, respectively. **e,** Co-IP analysis of SIX1 KO HepG2 or ZR75-1 cells co-transfected with empty vector, FLAG-SIX1, FLAG-SIX1 (S225A) or FLAG-SIX1 (S225K) and MYC-AIB1 or MYC-HBO1 as indicated. **f,** Co-IP analysis of HEK293T cells transfected with FLAG-SIX1, FLAG-SIX1 (S225K) or FLAG-SIX1 (S225A) and GFP-EYA4 as indicated.


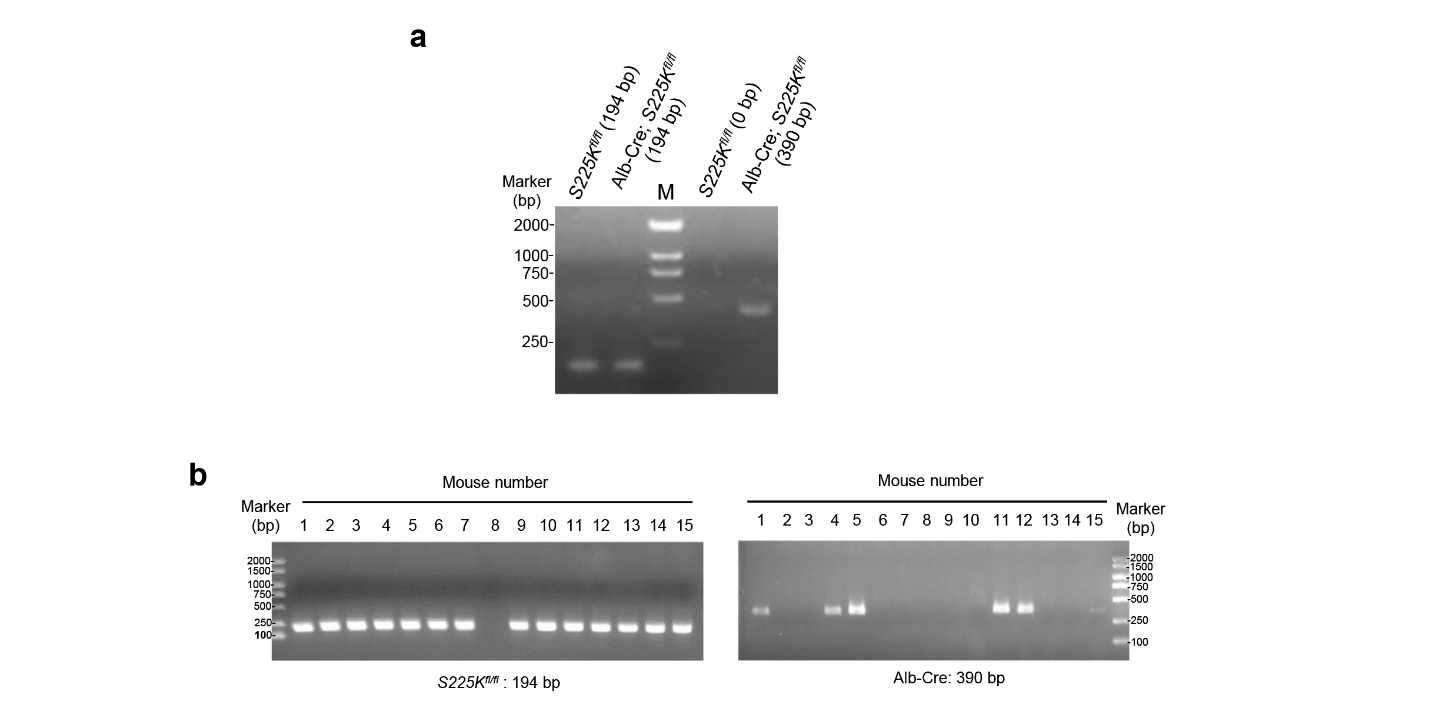


**Fig. S7. The genotype identification of *S225K^fl/fl^* mice and Alb-Cre; *S225K^fl/fl^* mice by PCR.** **a**, PCR products of 194 bp and 390 bp were observed in a representative Alb-Cre; *S225K^fl/fl^* mouse, and only 194 bp PCR product was observed in a representative *S225K^fl/fl^* mouse. M, markers. **b**, More results of mouse genotype identification are shown. The mouse number of 2, 3, 6, 7, 9, 10, 13 and 14 was *S225K^fl/fl^* mice and the mouse number of 1, 4, 5, 11, 12 and 15 was Alb-Cre; *S225K^fl/fl^* mice.


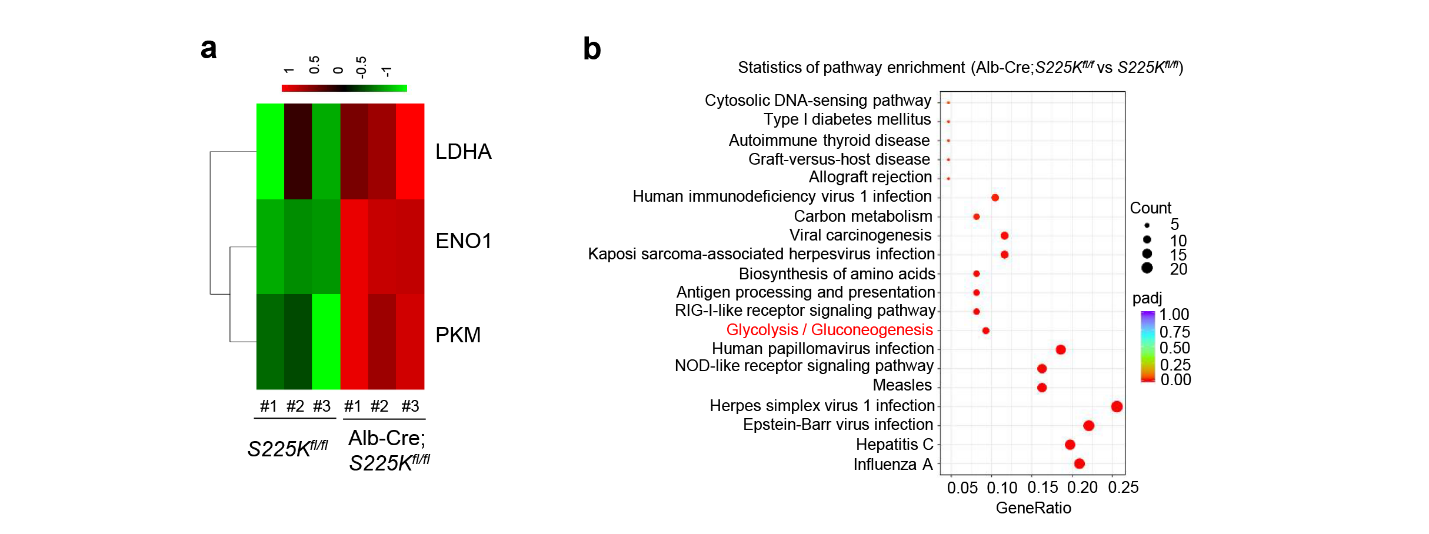


**Fig. S8. The SIX1 (S225K) mutant regulates glycolytic gene expression and glycolytic pathway in mice. a,** Heatmap of glycolytic genes identified by RNA-seq using DEN-induced liver tumor tissues from Alb-Cre;*S225K^fl/f^* mice and *S225K^fl/fl^* mice (n = 3). **b,** KEGG pathway analysis of genes differentially expressed between DEN-induced liver tumors from mice as in (a).


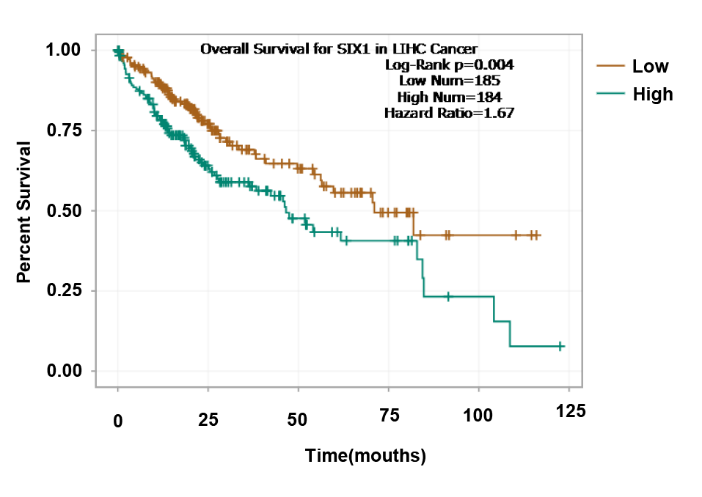


**Fig. S9. The overall survival of SIX1 in patients with liver cancer.** Overall survival curves for SIX1 in patients with LIHC (Liver Hepatocellular Carcinoma) was plotted based on ENCORI (Encyclopedia of RNA Interactomes) database (https://rnasysu.com/encori/) in which data of cancers were from TCGA (The Cancer Genome Atlas) project.

**Supplementary Table 1. The cDNA target sequences of shRNAs or siRNAs**

| **Gene** | **Target sequence (5’→3’)** |
| --- | --- |
| ERK2 siRNA1  ERK2 siRNA2  EYA4 siRNA1  EYA4 siRNA2  PKM2 shRNA | CACUUGUCAAGAAGCGUUA  CAUGGUAGUCACUAACAUA  GAGUGGACUUUCCCAAACU  GGAGCGUAUAUGACAUCGA  GCTGTGGCTCTAGACACTA |

**Supplementary Table 2. Primers used for quantitative real-time PCR**

| **Gene** | **Species** | **Forward (5’→3’)** | **Reverse (5’→3’)** |
| --- | --- | --- | --- |
| HK2  PKM2  LDHA  β-actin  HK2  PKM2  LDHA  β-actin | Human  Human  Human  Human  Mouse  Mouse  Mouse  Mouse | GCCATCCTGCAACACTTAGGGCTTGAG  GCCCGTGAGGCAGAGGCTGC ATGGCAACTCTAAAGGATCA  TCGTGCGTGACATTAAGGAG  CCTGCTACAGGTCCGAGCCATCTT  TCGCATGCAGCACCTGATT  GCAGACAAGGAGCAGTGGAAGGAG  CCACAGCTGAGAGGGAAATC | GTGAGGATGTAGCTTGTAGAGGGTCCC  TGGTGAGGACGATTATGGCCC  GCAACTTGCAGTTCGGGC  ATGCCAGGGTACATGGTGGT  GAGGATGAAGCTTGTACAGTGTCC  CCTCGAATAGCTGCAAGTGGTA  ACACTGAGGAAGACATCCTCATTG  AAGGAAGGCTGGAAAAGAGC |

**Supplementary Table 3. Primers used for ChIP**

| **Gene** | **Forward (5’→3’)** | **Reverse (5’→3’)** |
| --- | --- | --- |
| HK2  PKM2  LDHA  HK2 upstream  PKM2 upstream  LDHA upstream | AGGAAGAGCAAAGACCCTTGGGTG  TACAGGCGTGAGCCACTGCACC  GATCAGCCTGACCAACATGGTGAAA  GTGAAGAAAGTATCATAGCTGACC  GTATTTGTTTAACTGTCCCTCCCC  GAATAGTGCTGCAATAGTCATGGG | GCCACTCACCCTCACAGCCAGTC  AAAAGACATGCCCCCCTAGGGAG  TGAAACGGAGTCGCTCTGTCGCC  GGAAGGTTATGGGTTTAGGTATTG  TCAGTTTCCCCAGACAAATCCTGG  GGCCACATCCAAAGAACACTTGGT |
