# Supplementary material for: Phosphorylation determines the glucose metabolism reprogramming and tumor-promoting activity of sine oculis homeobox 1
Source: Signal Transduct Target Ther. 2024 Dec 2;9:337. doi: 10.1038/s41392-024-02034-5 (PMC11609306; doi:10.1038/s41392-024-02034-5)

Uncut images for Figures

Fig. 1b

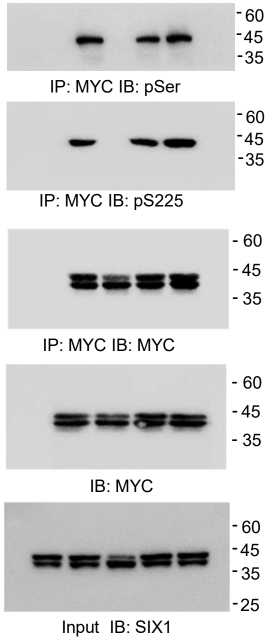

Fig. 1c

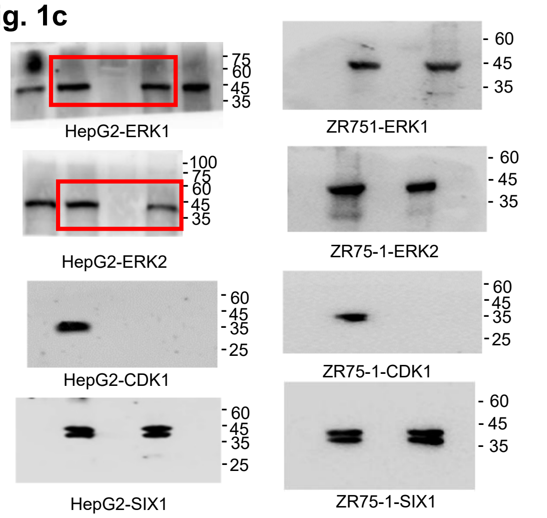

Fig. 1d

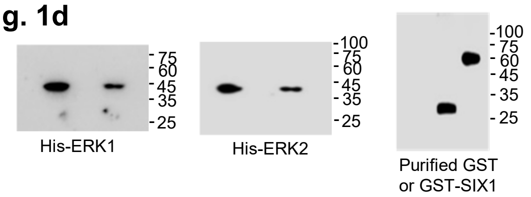

Fig. 1e

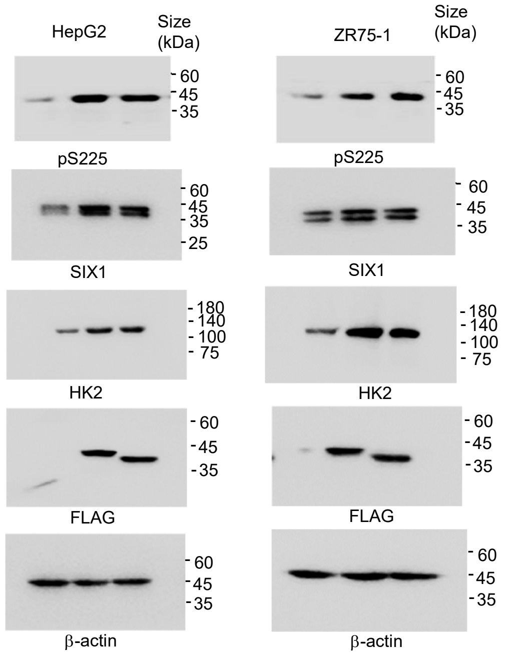

Fig. 1f

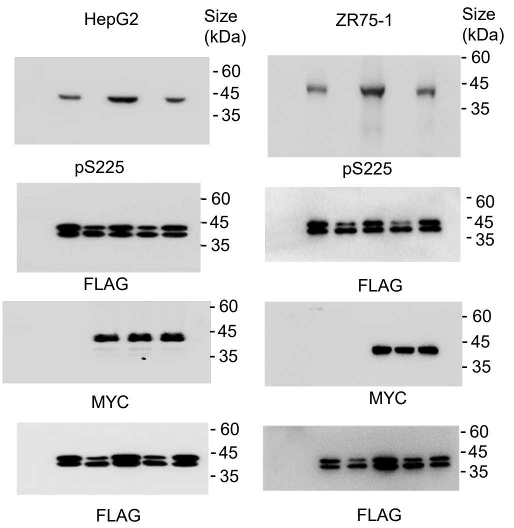

Fig. 1g

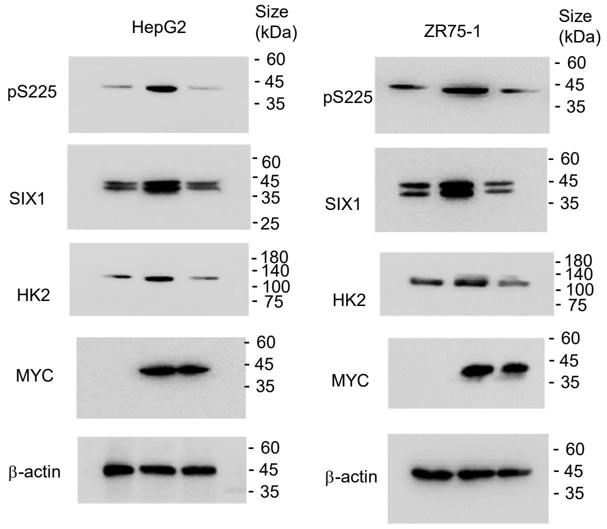

Fig. 1h

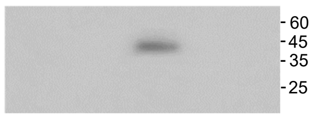

Fig. 1i

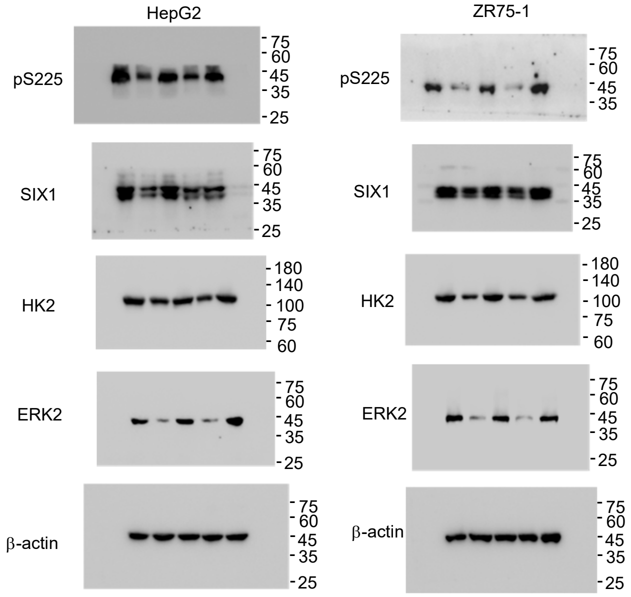

Fig. 1j

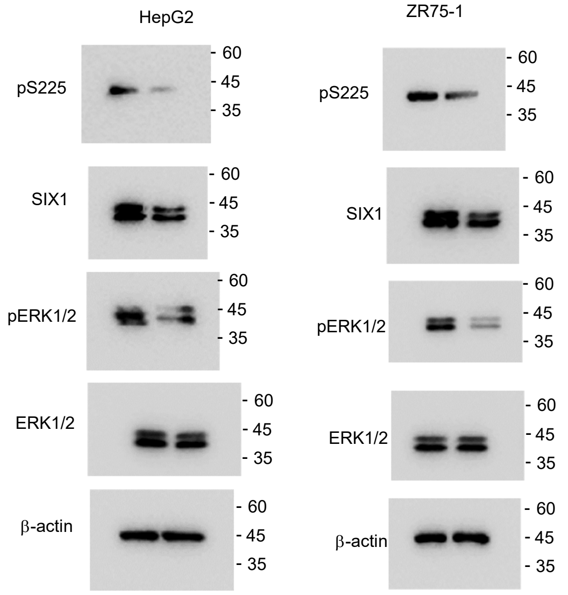

Fig. 2a

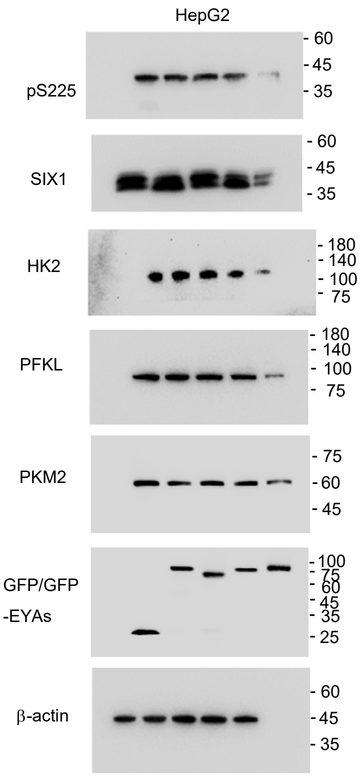

Fig. 2b

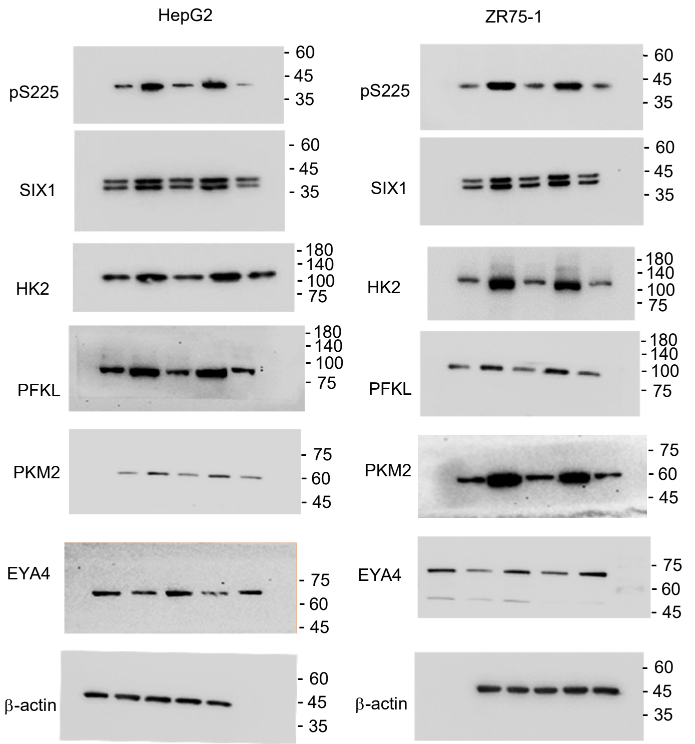

Fig. 2c

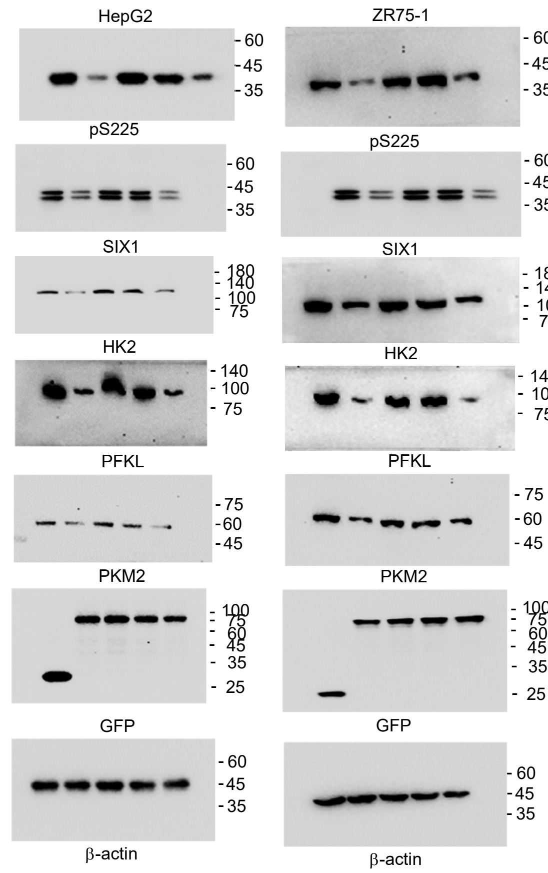

Fig. 3a

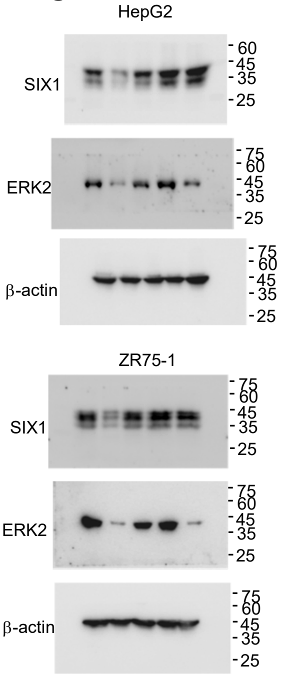

Fig. 3b

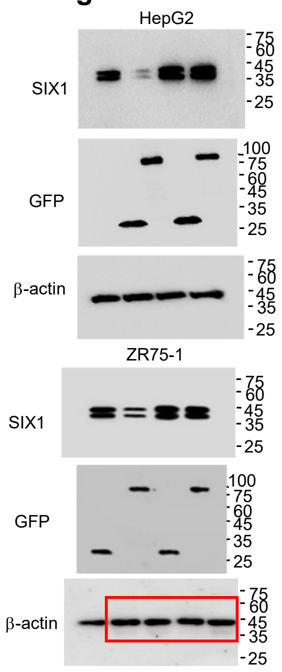

Fig. 3c

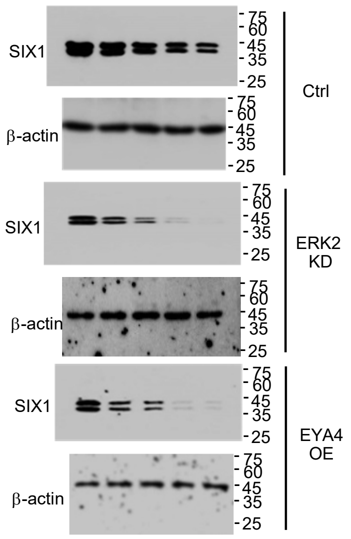

Fig. 2d

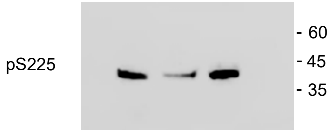

Fig. 3d

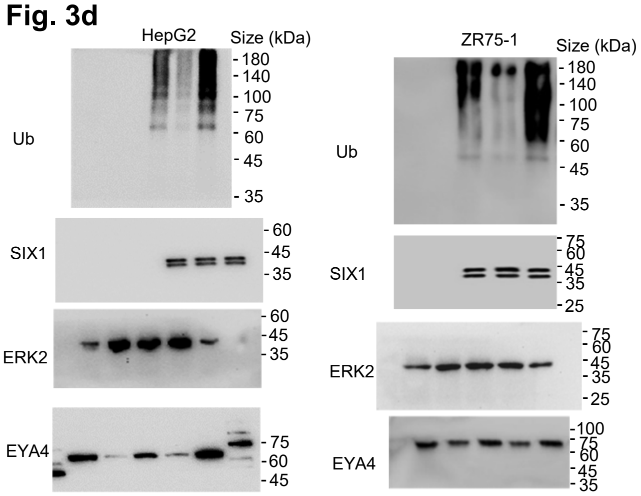

Fig. 3e

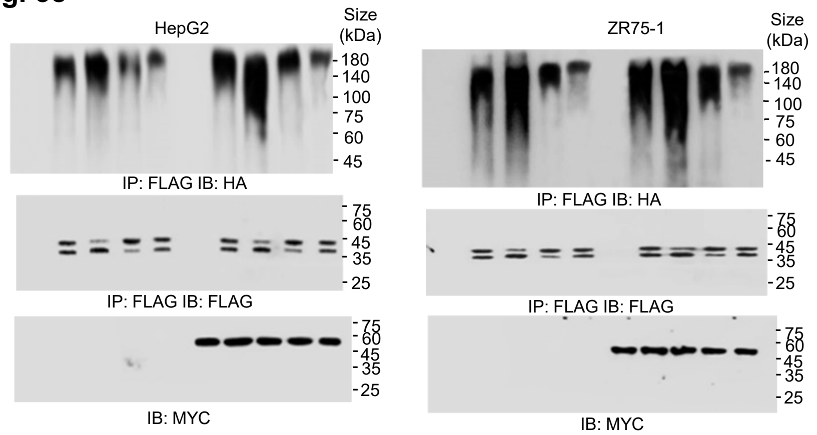

Fig. 3f

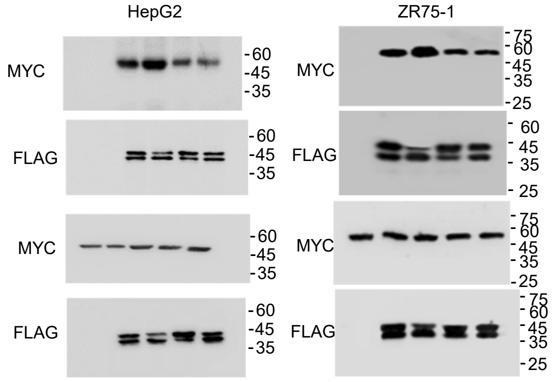

Fig. 3g

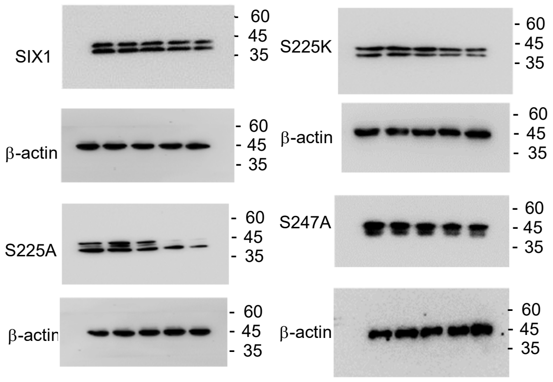

Fig. 3h

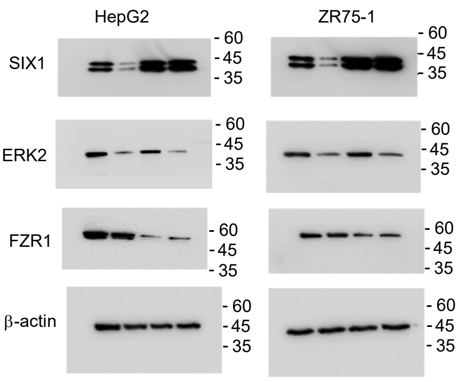

Fig. 3i

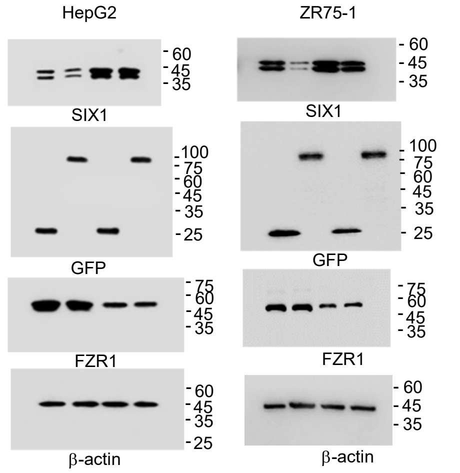

Fig. 4a

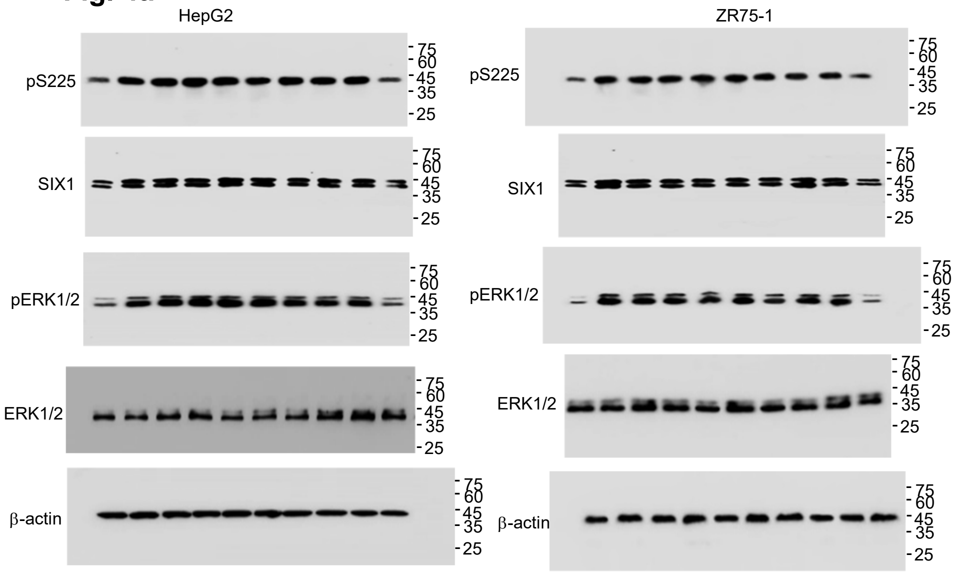

Fig. 4b

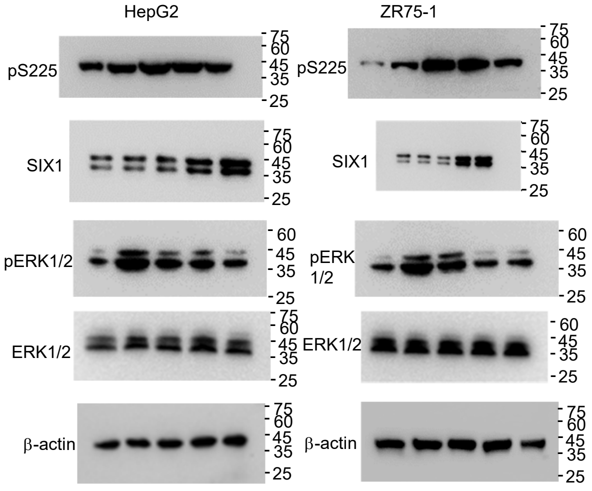

Fig. 4c

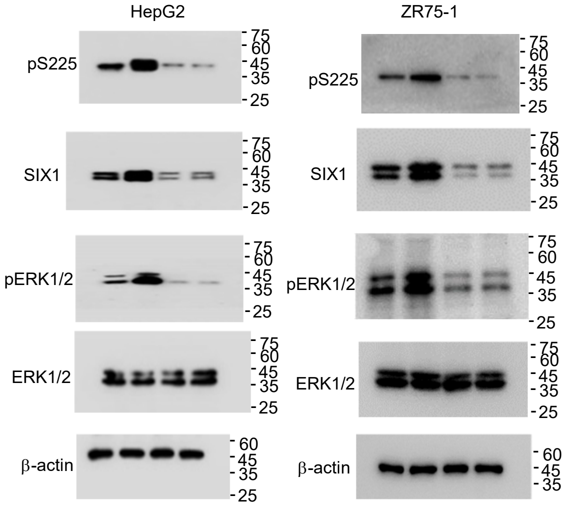

Fig. 4d

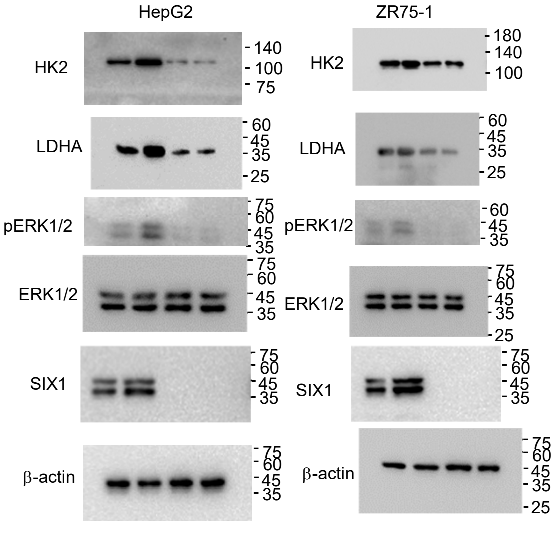

Fig. 4f

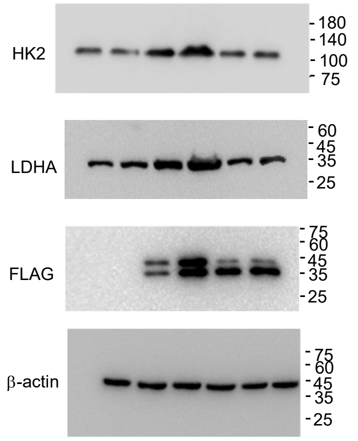

Fig. 4h

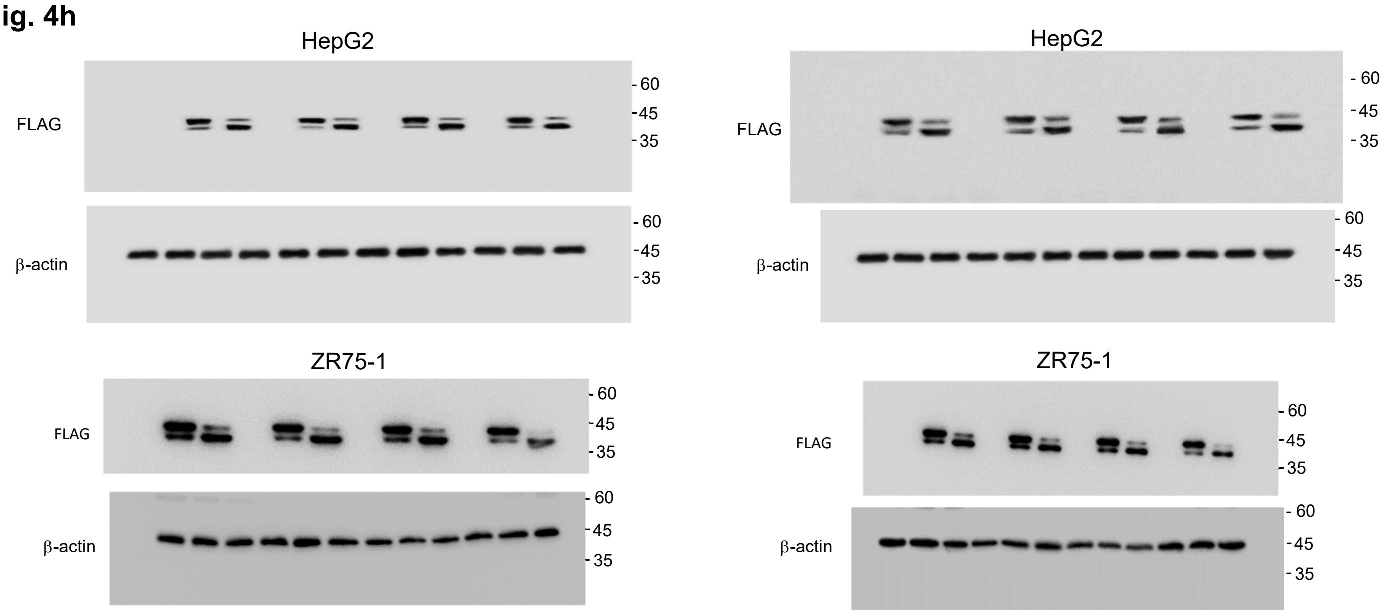

Fig. 5a

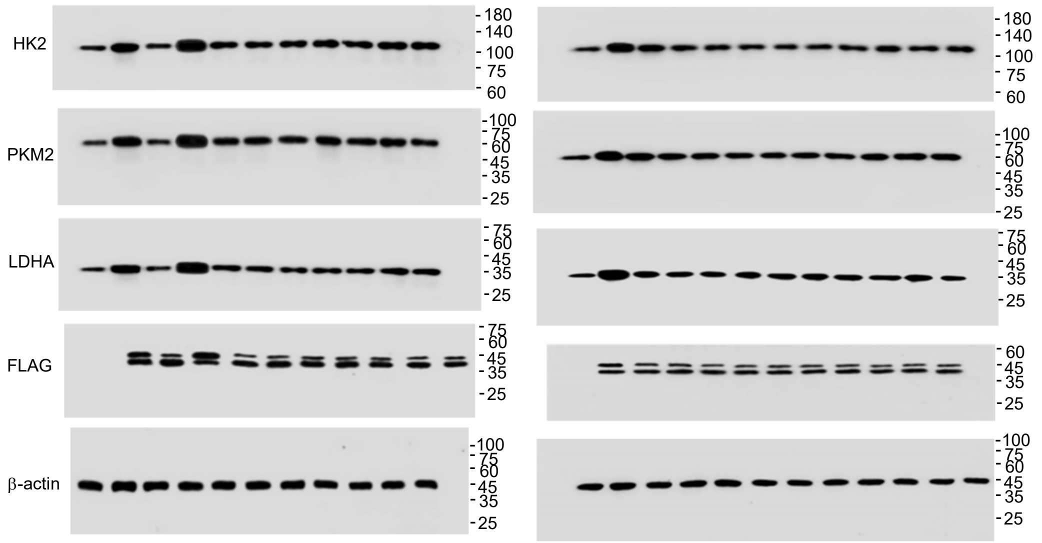

Fig. 5c

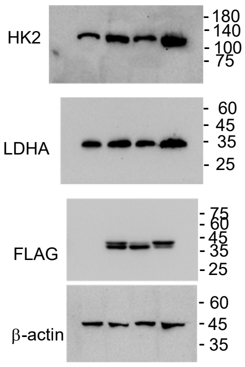

Fig. 5e

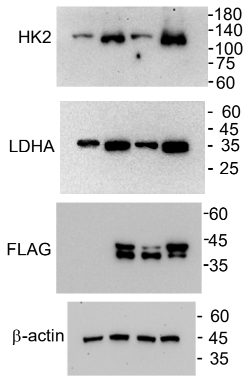

Fig. 5f

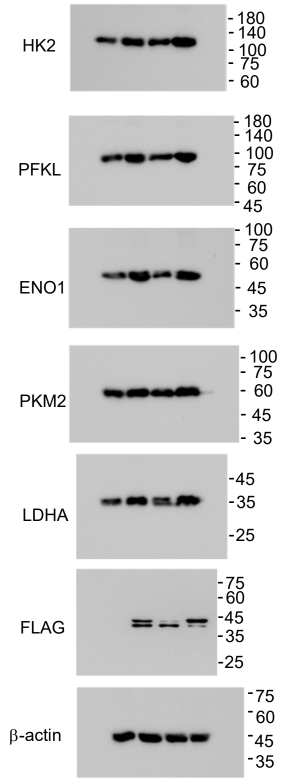

Fig. 5g

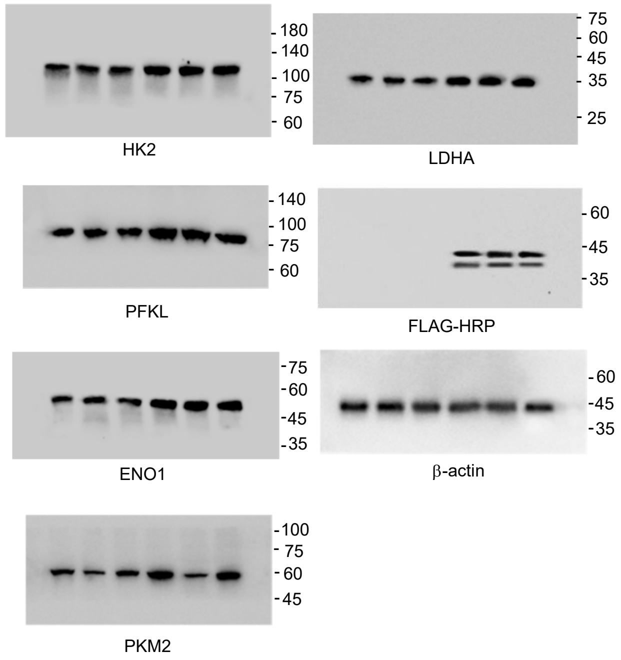

Fig. 6f

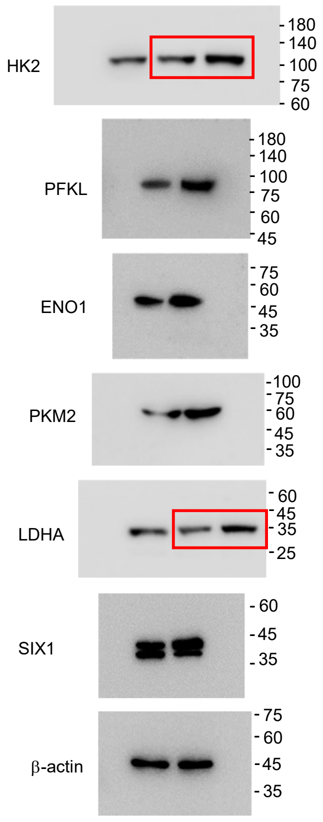

Uncut images for Supplementary Figures

Fig. S1a

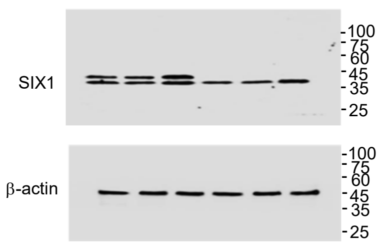

Fig. S1b

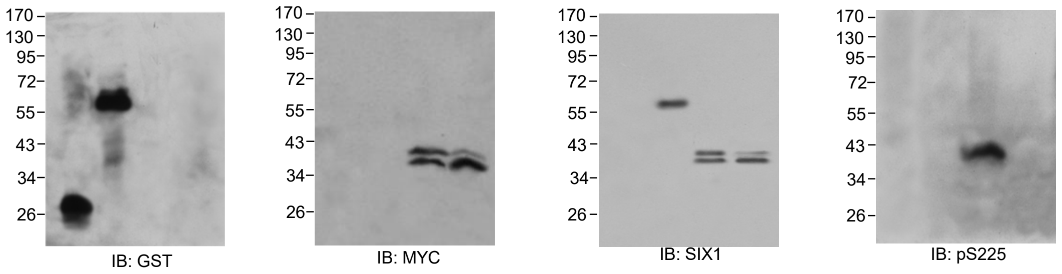

Fig. S1c

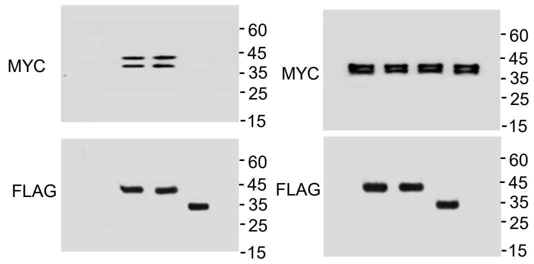

Fig. S1d

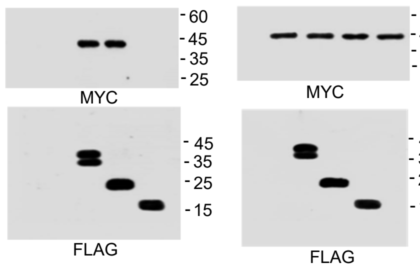

Fig. S1f

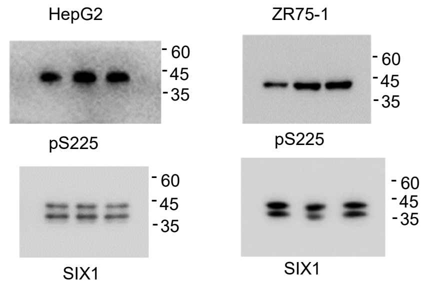

Fig. S1e

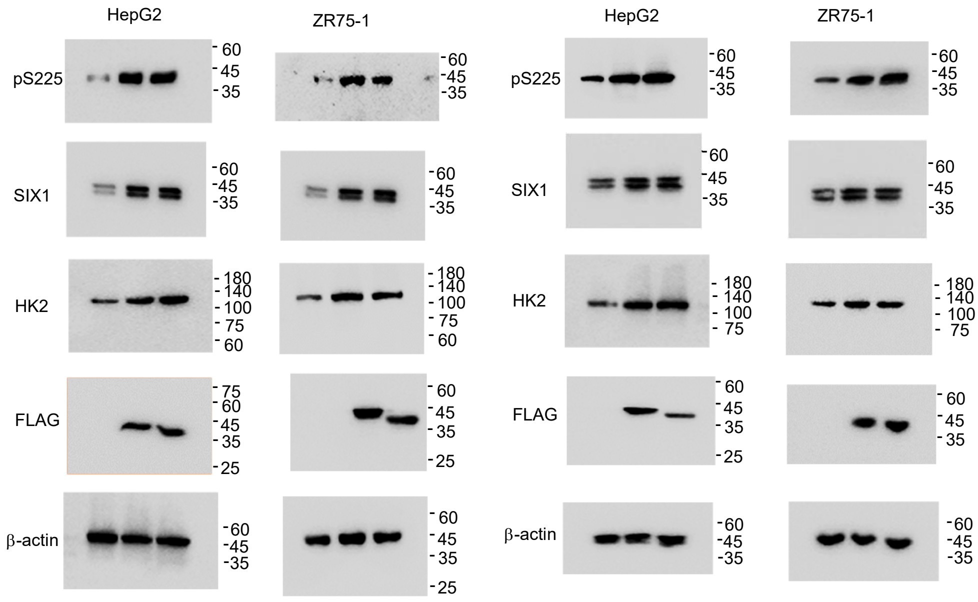

Fig. S1g

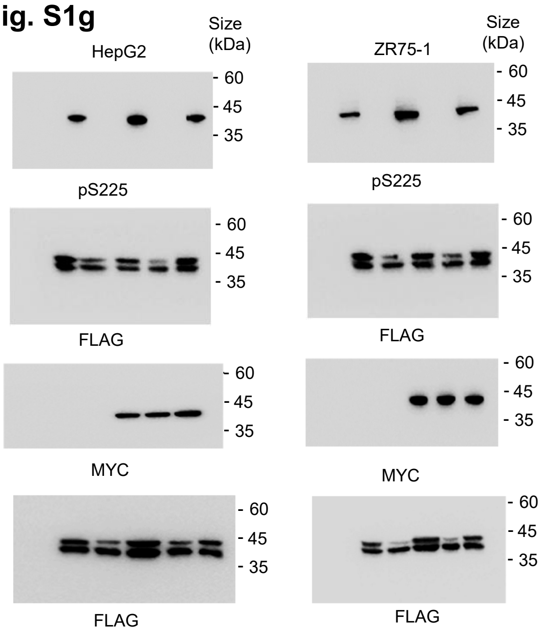

Fig. S1g

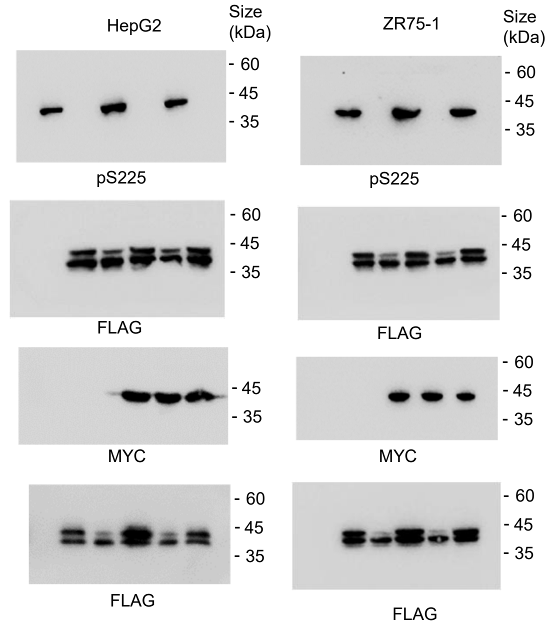

Fig. S1h

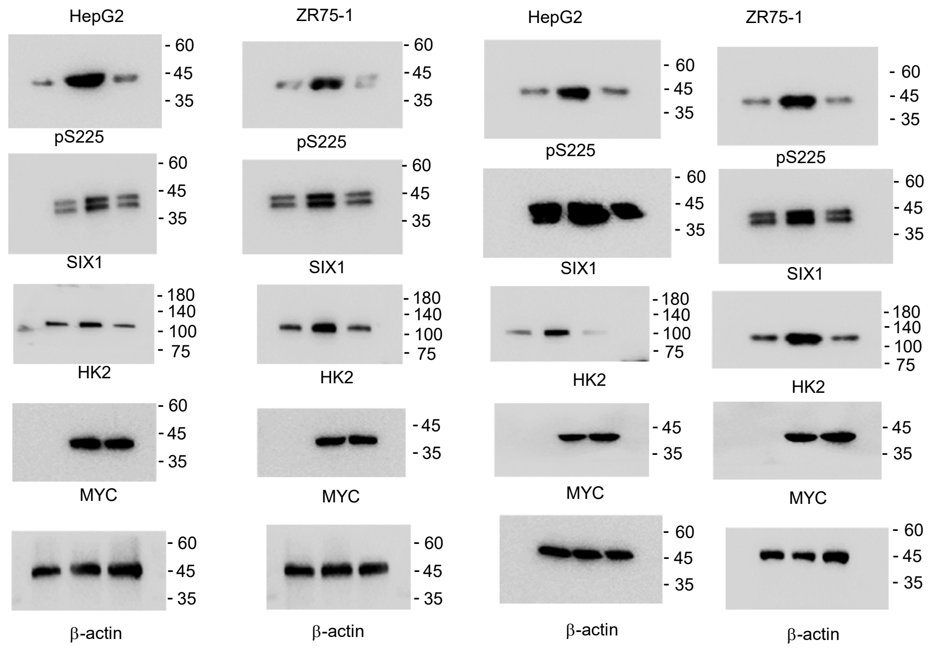

Fig. S1i

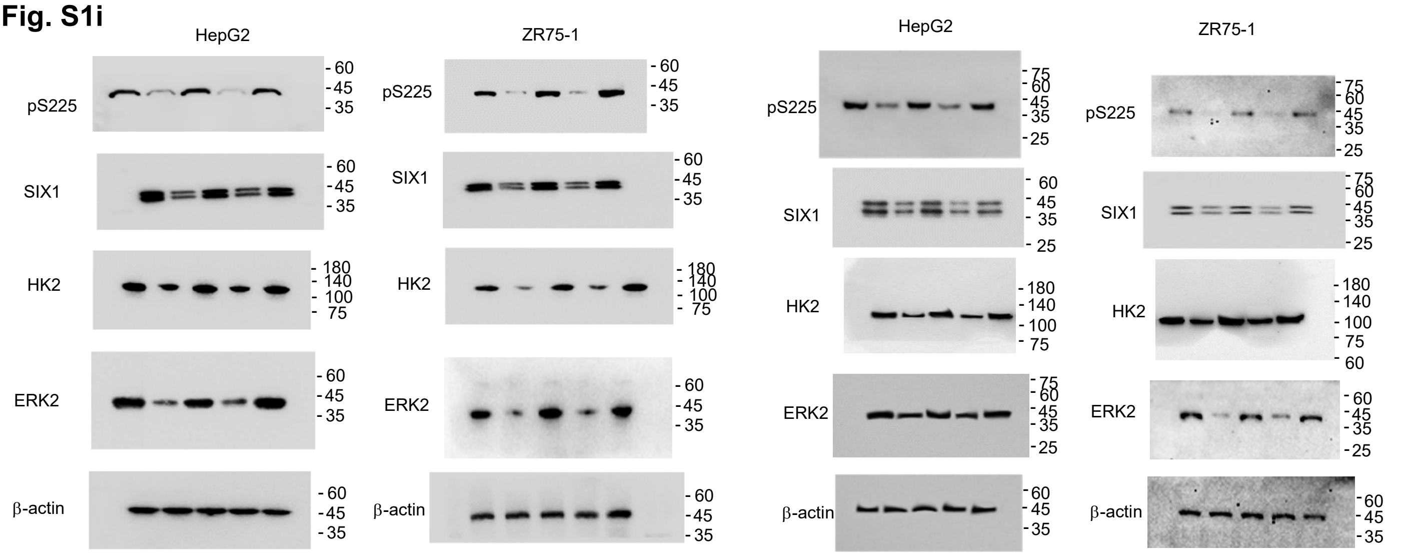

Fig. S1j

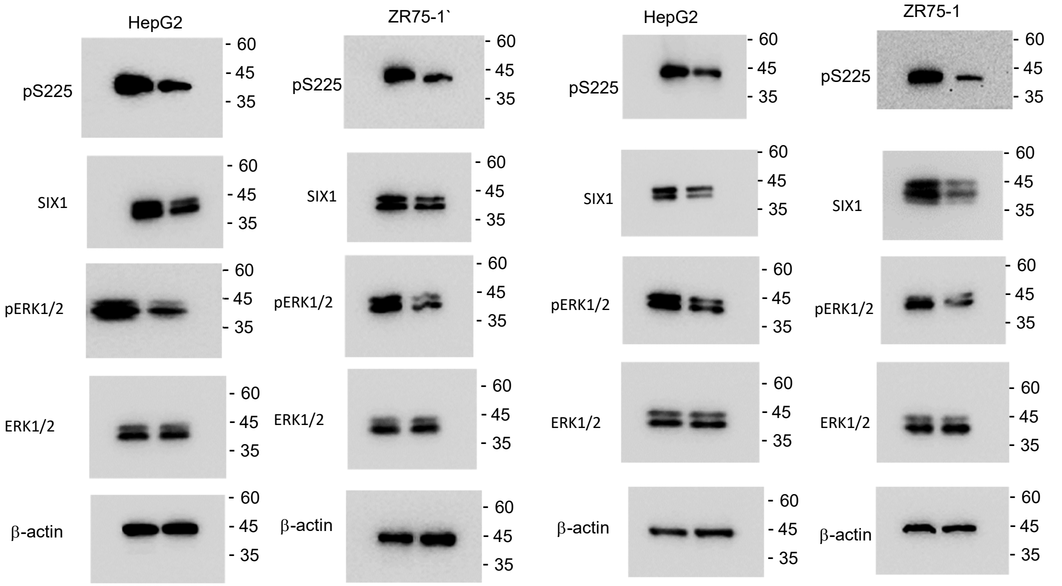

Fig. S1k

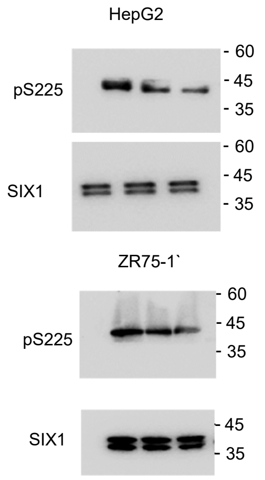

Fig. S2a

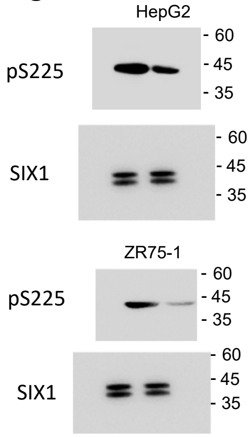

Fig. S2b

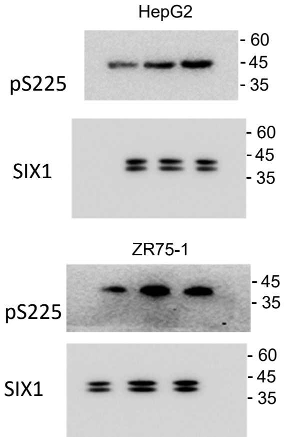

Fig. S2d

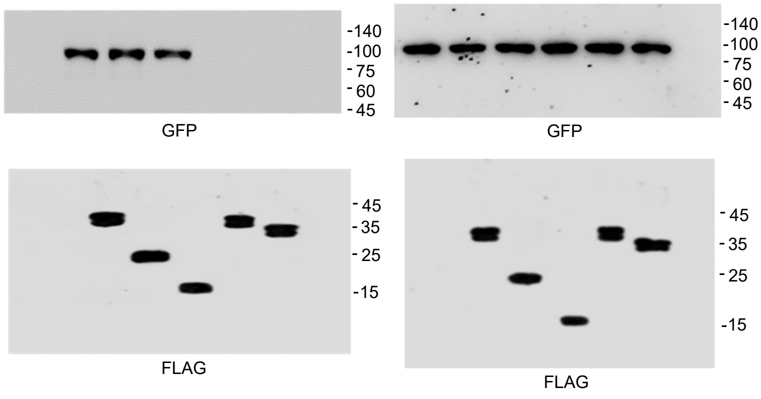

Fig. S3a

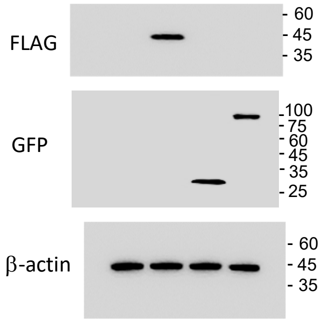

Fig. S3b

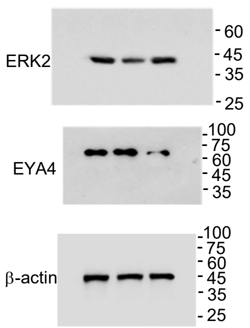

Fig. S3c

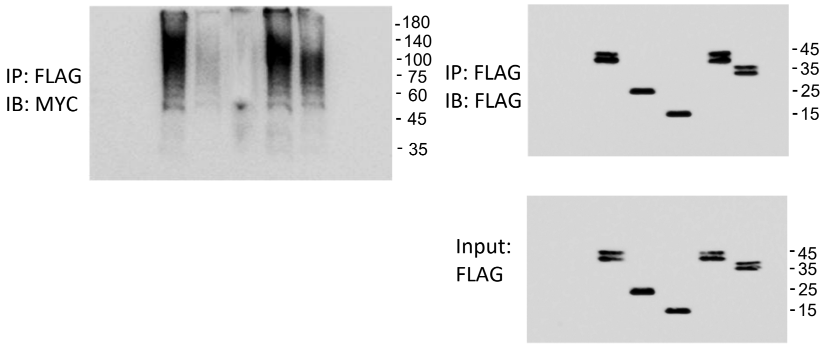

Fig. S3d

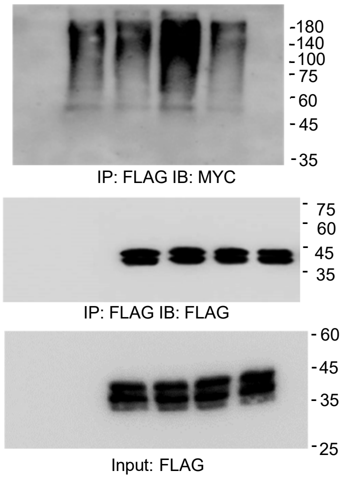

Fig. S3e

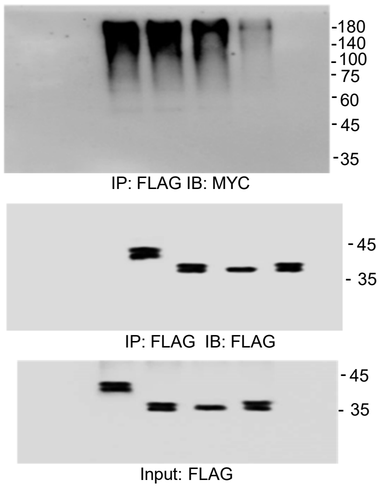

Fig. S3f

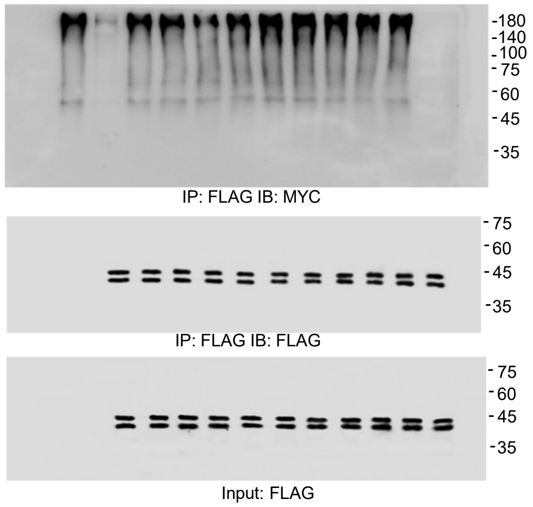

Fig. S3g

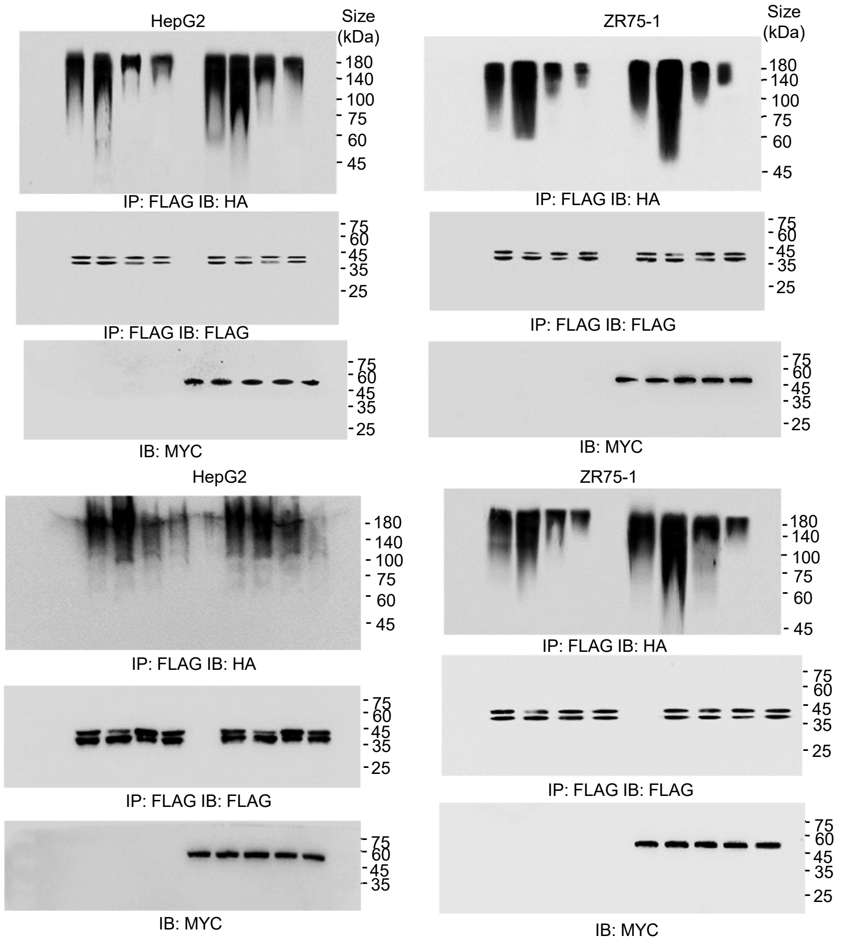

Fig. S3h

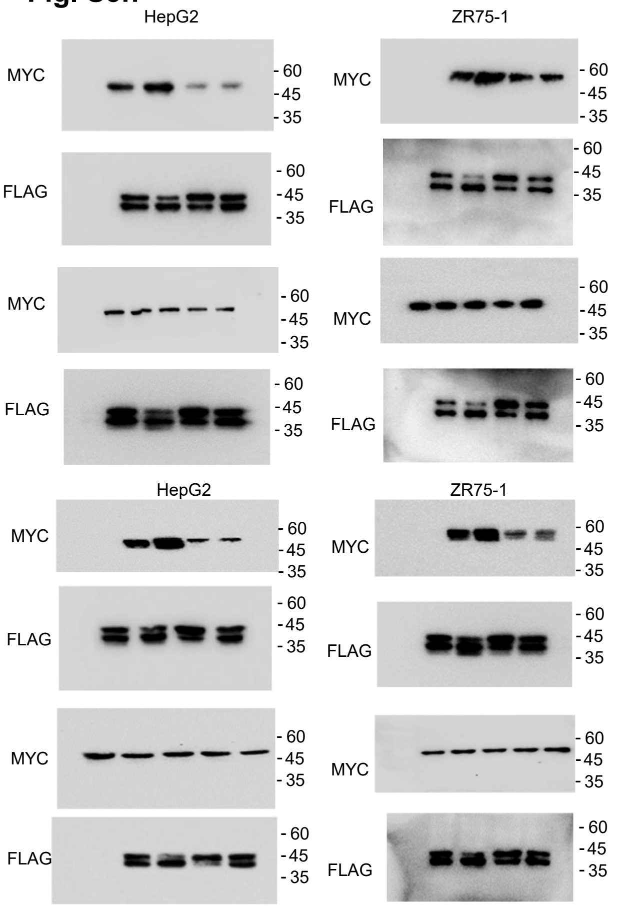

Fig. S4a

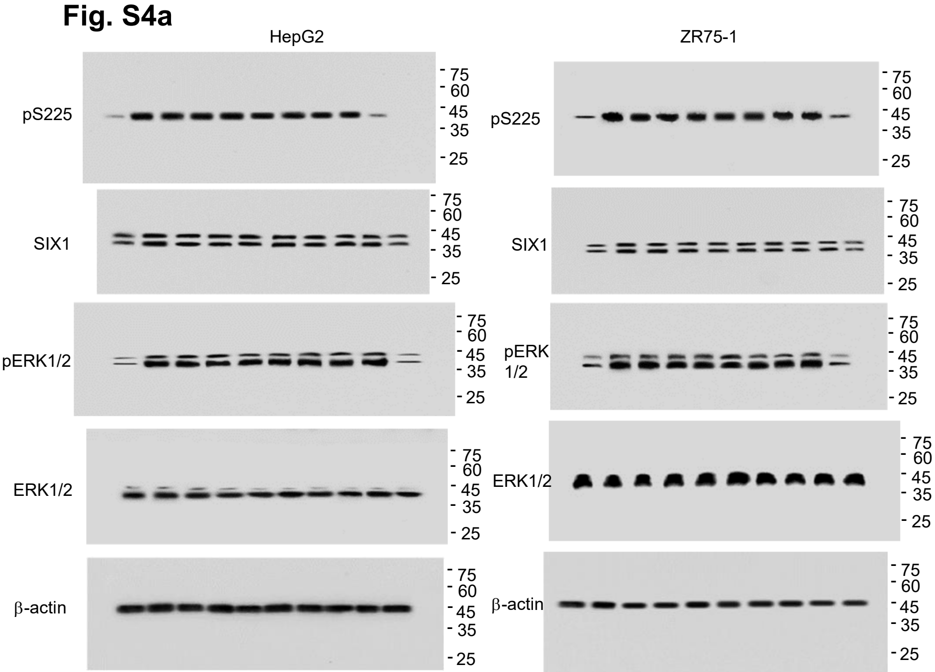

Fig. S4b

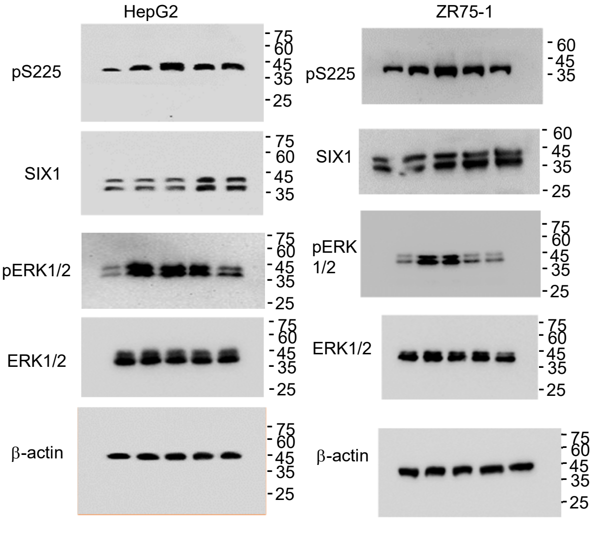

Fig. S4a

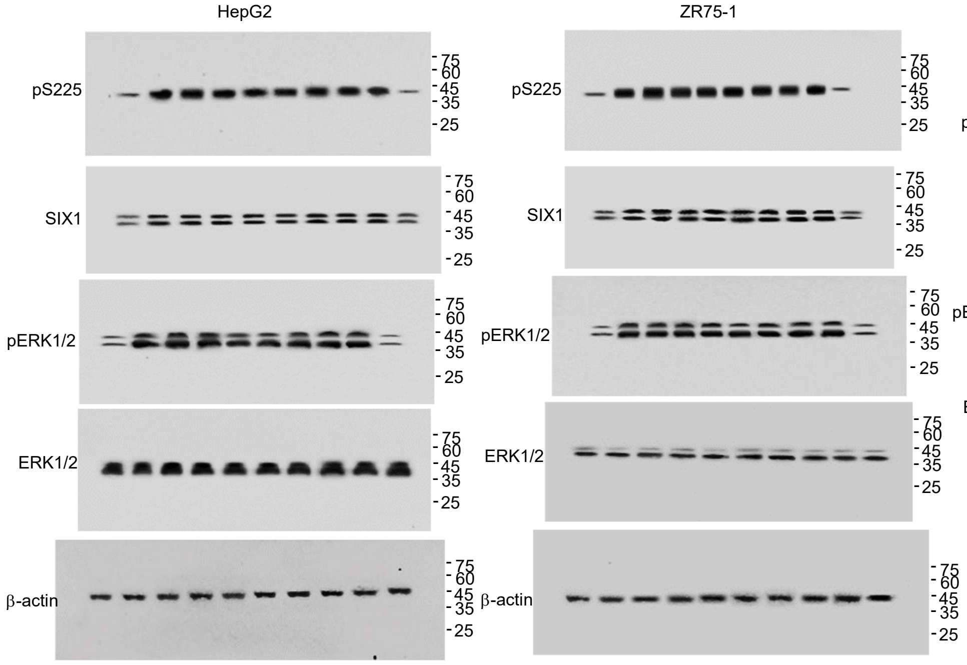

Fig. S4b

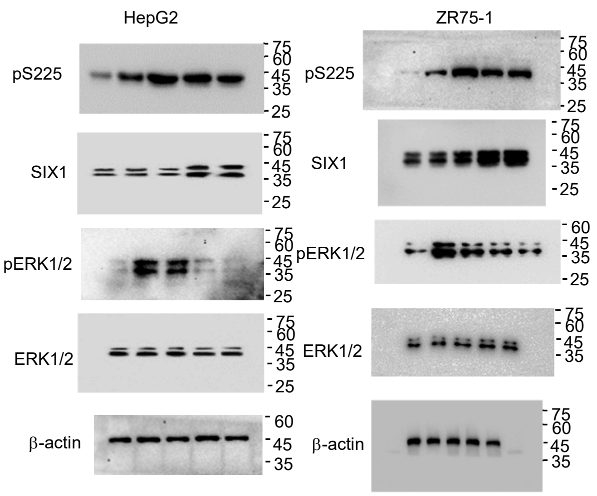

Fig. S4c

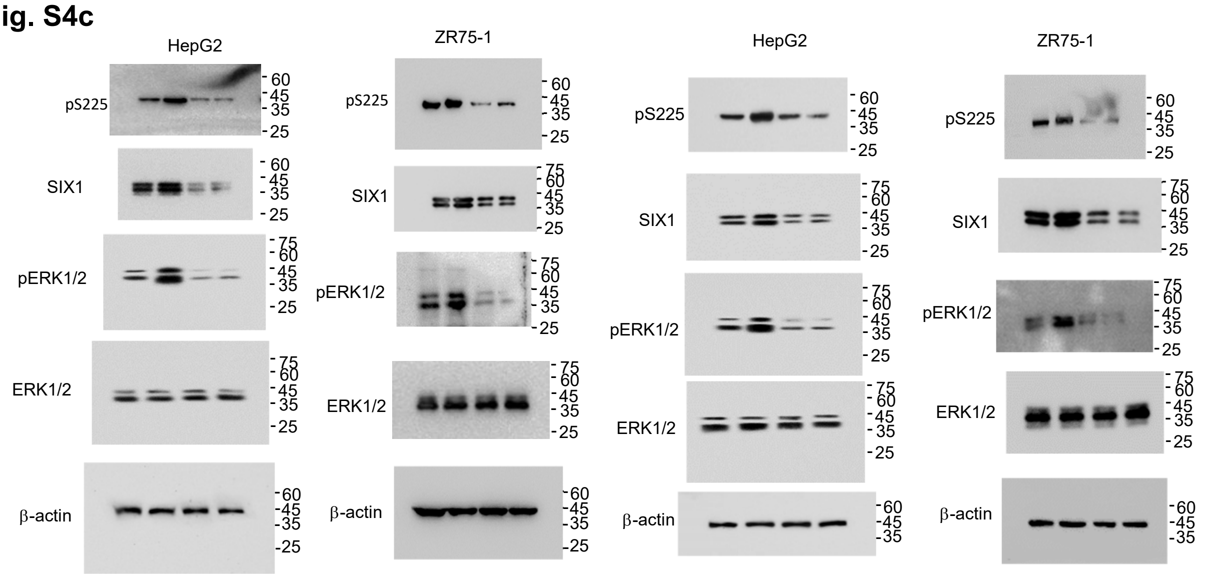

Fig. S4d

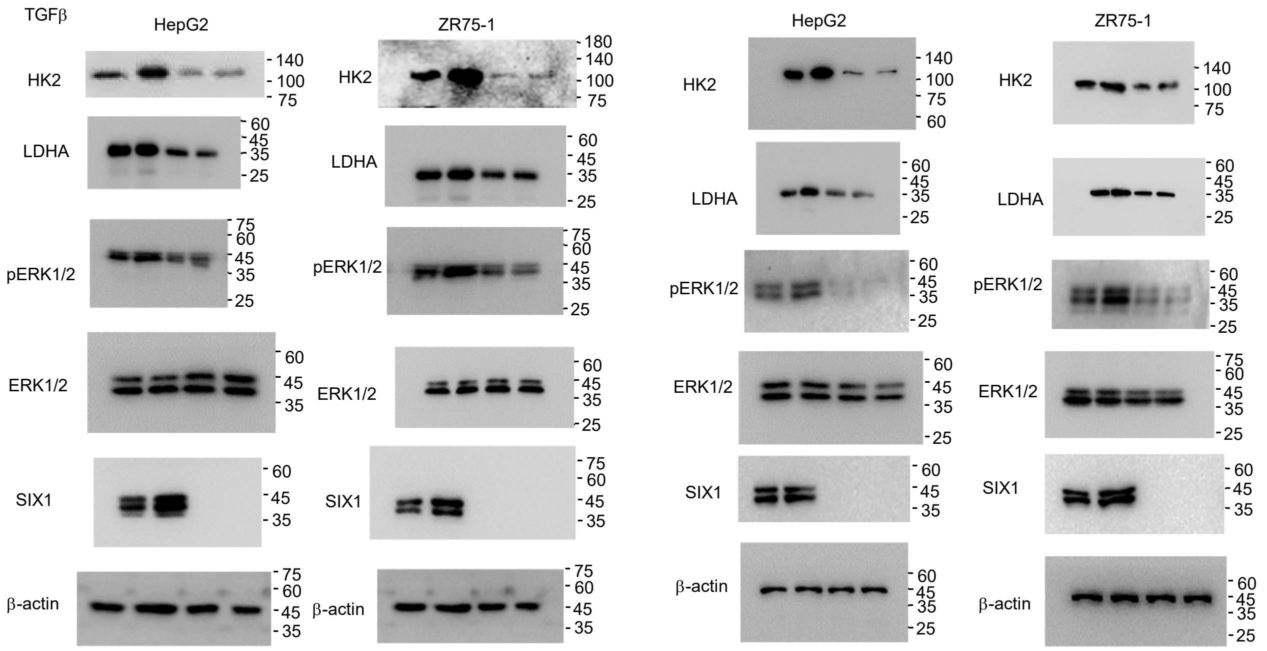

Fig. S4d

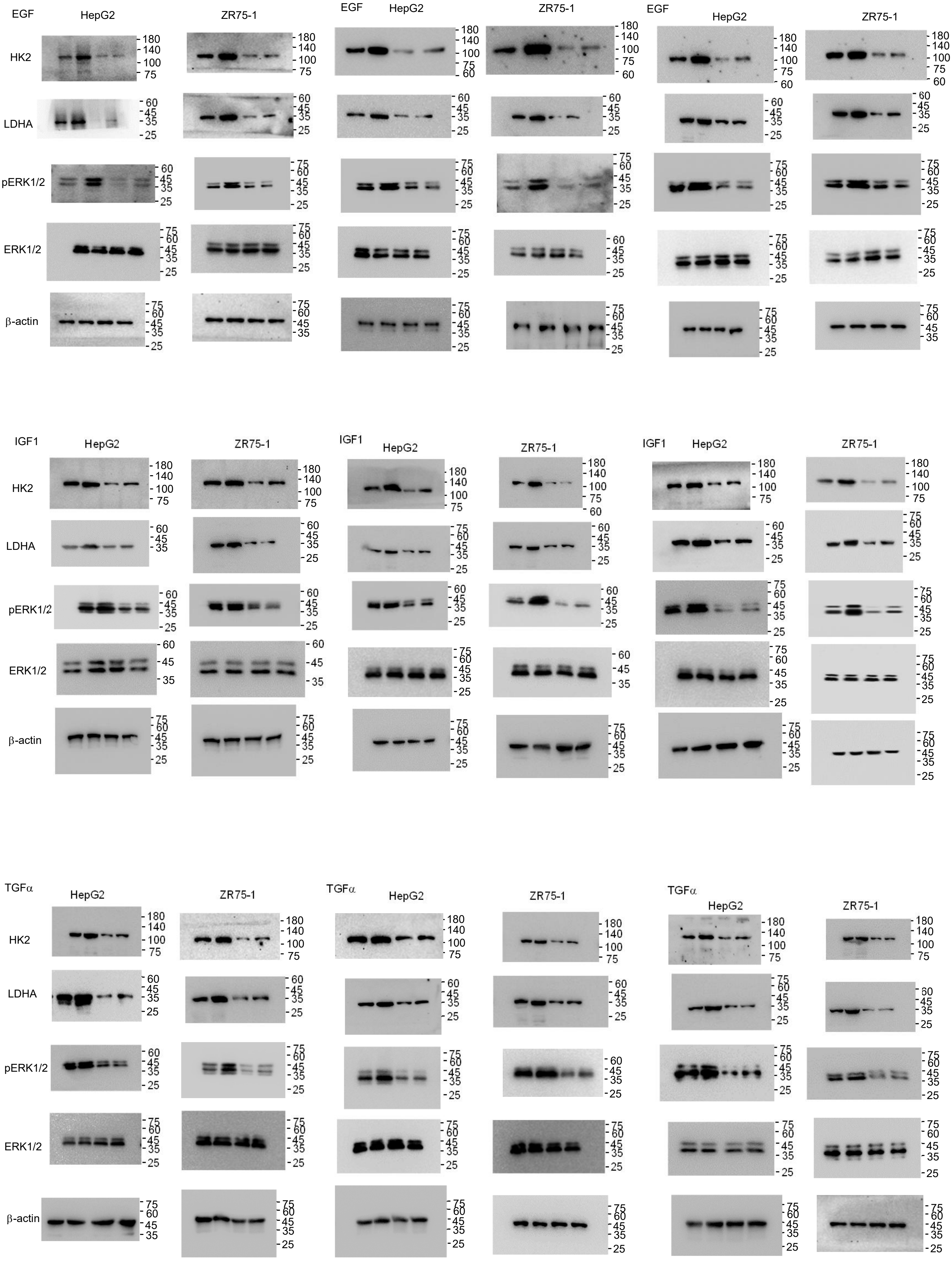
EGF
HepG2
ZR75-1
HK2
LDHA
pERK1/2
ERK1/2
β-actin
180
140
100
75
60
45
35
25
IGF1
TGFα

Fig. S4d

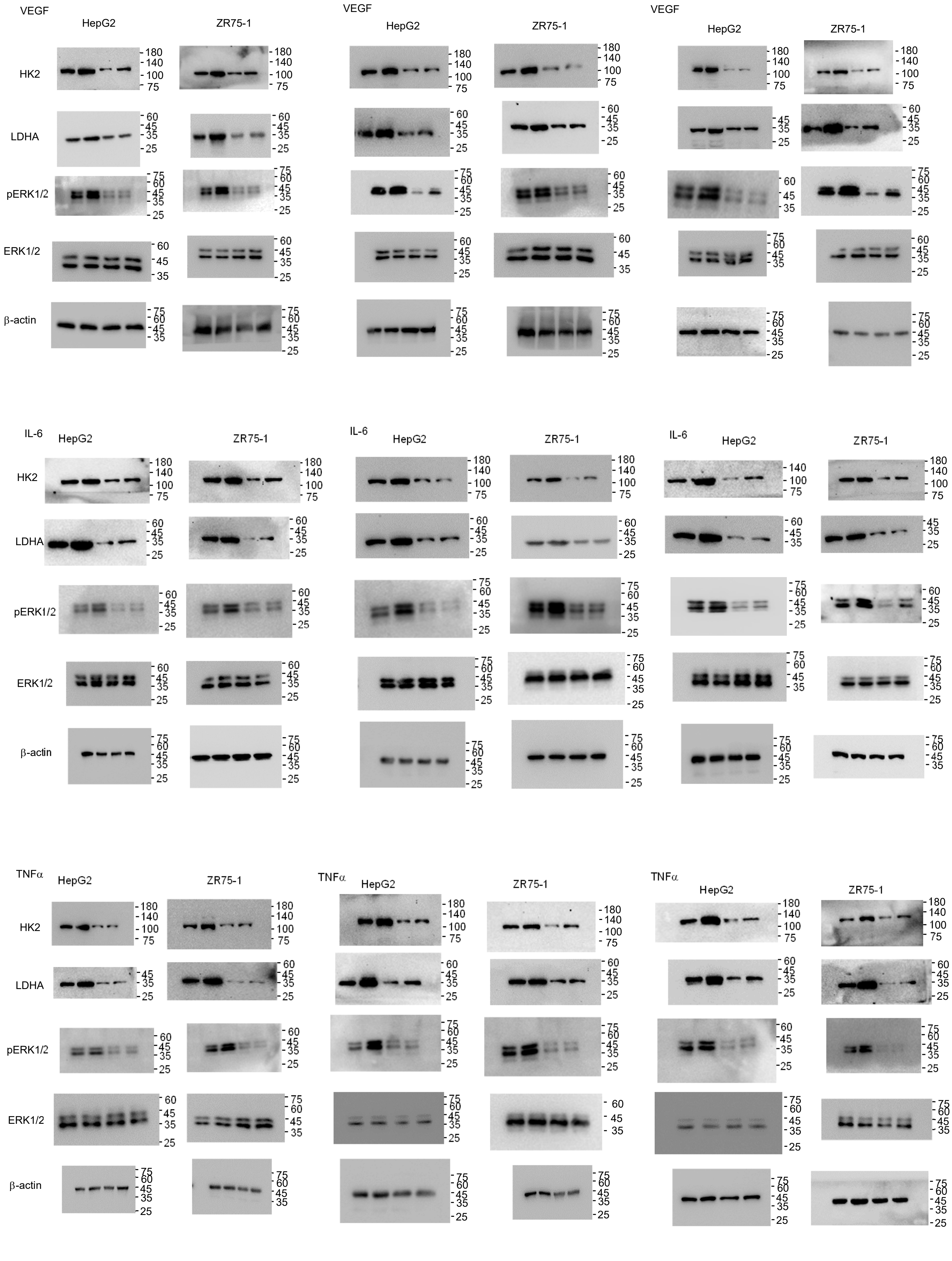

Fig. S4d

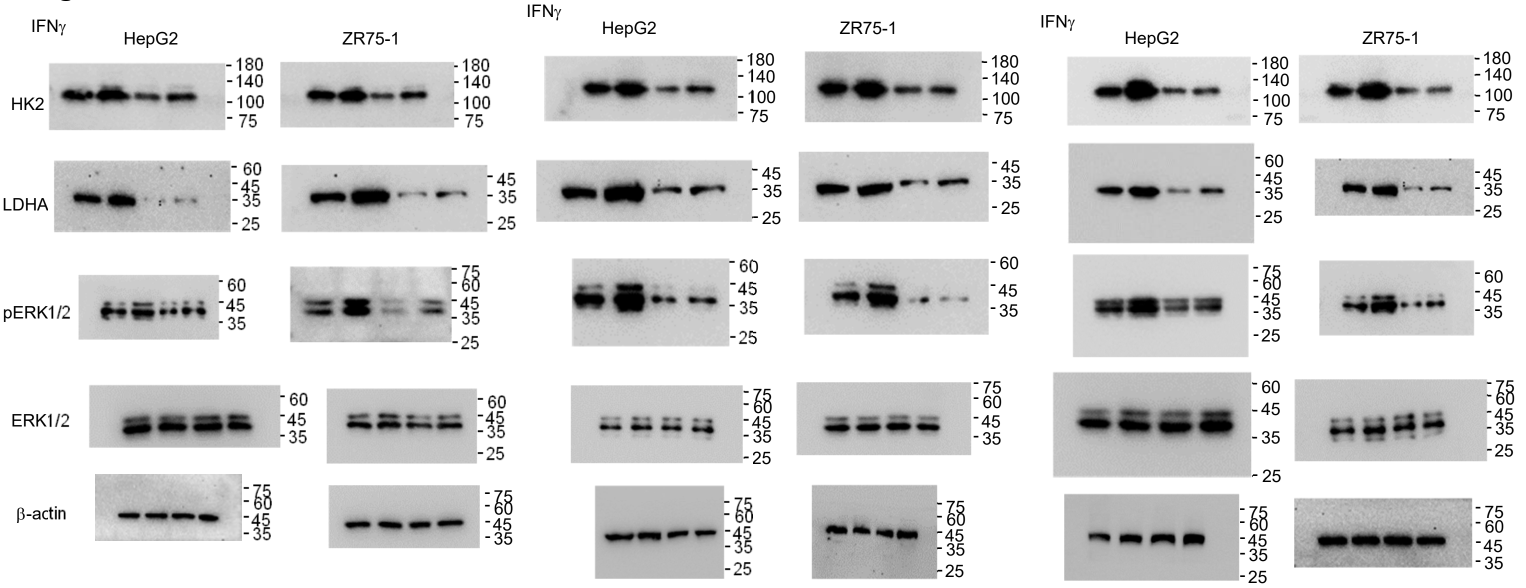

Fig. S4f

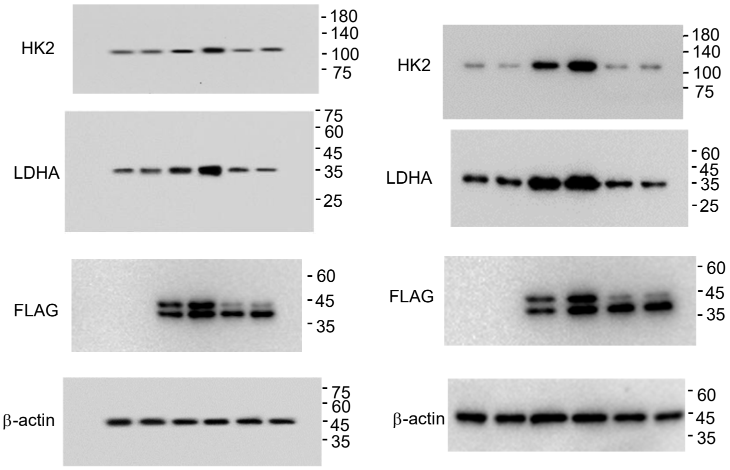

Fig. S4g

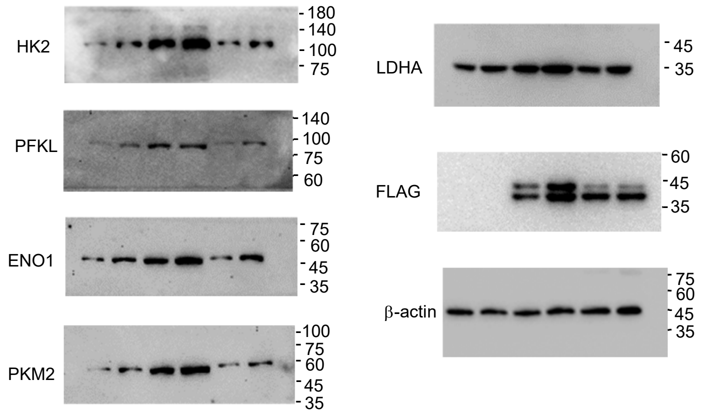

Fig. S5a

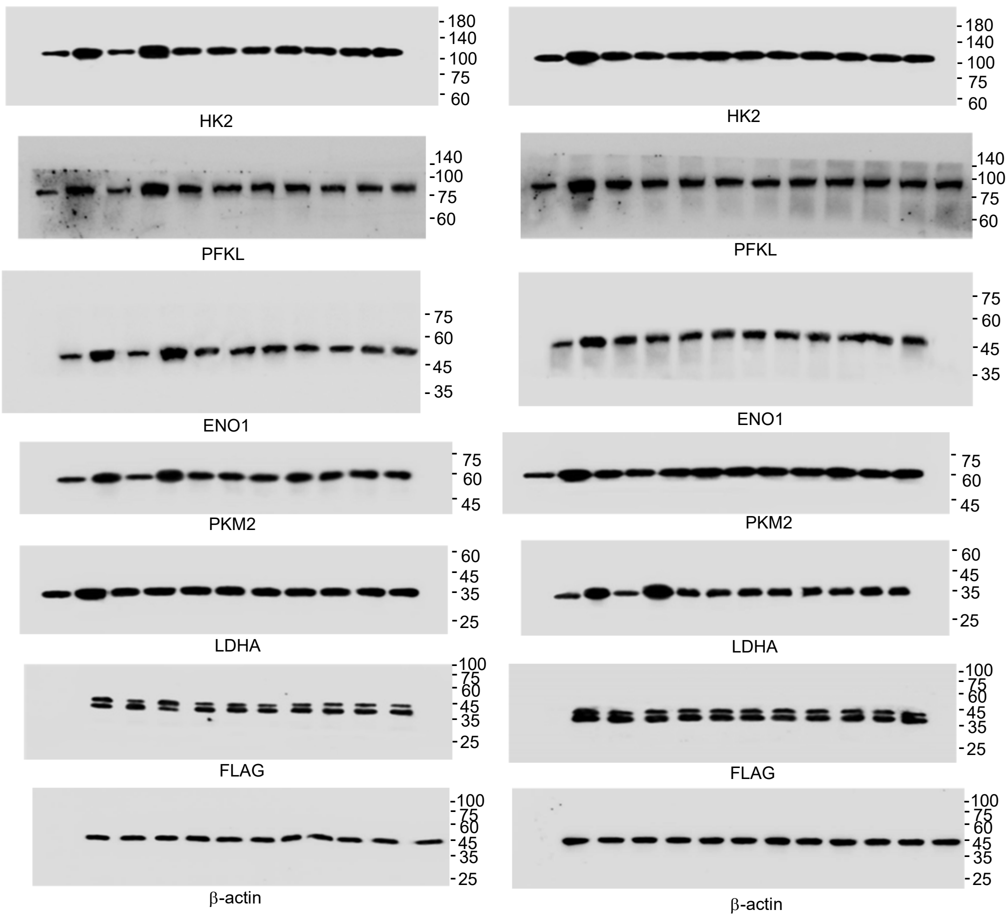

Fig. S5c

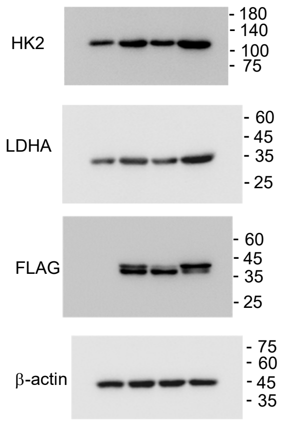

Fig. S5f

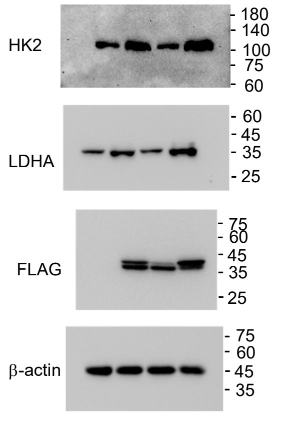

Fig. S5e

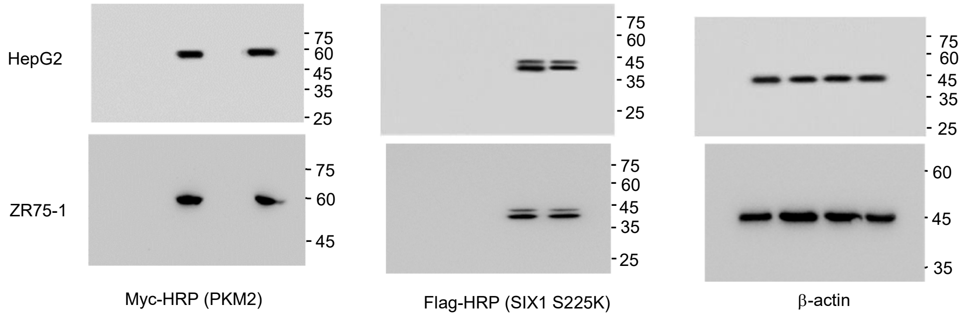

Fig. S6a

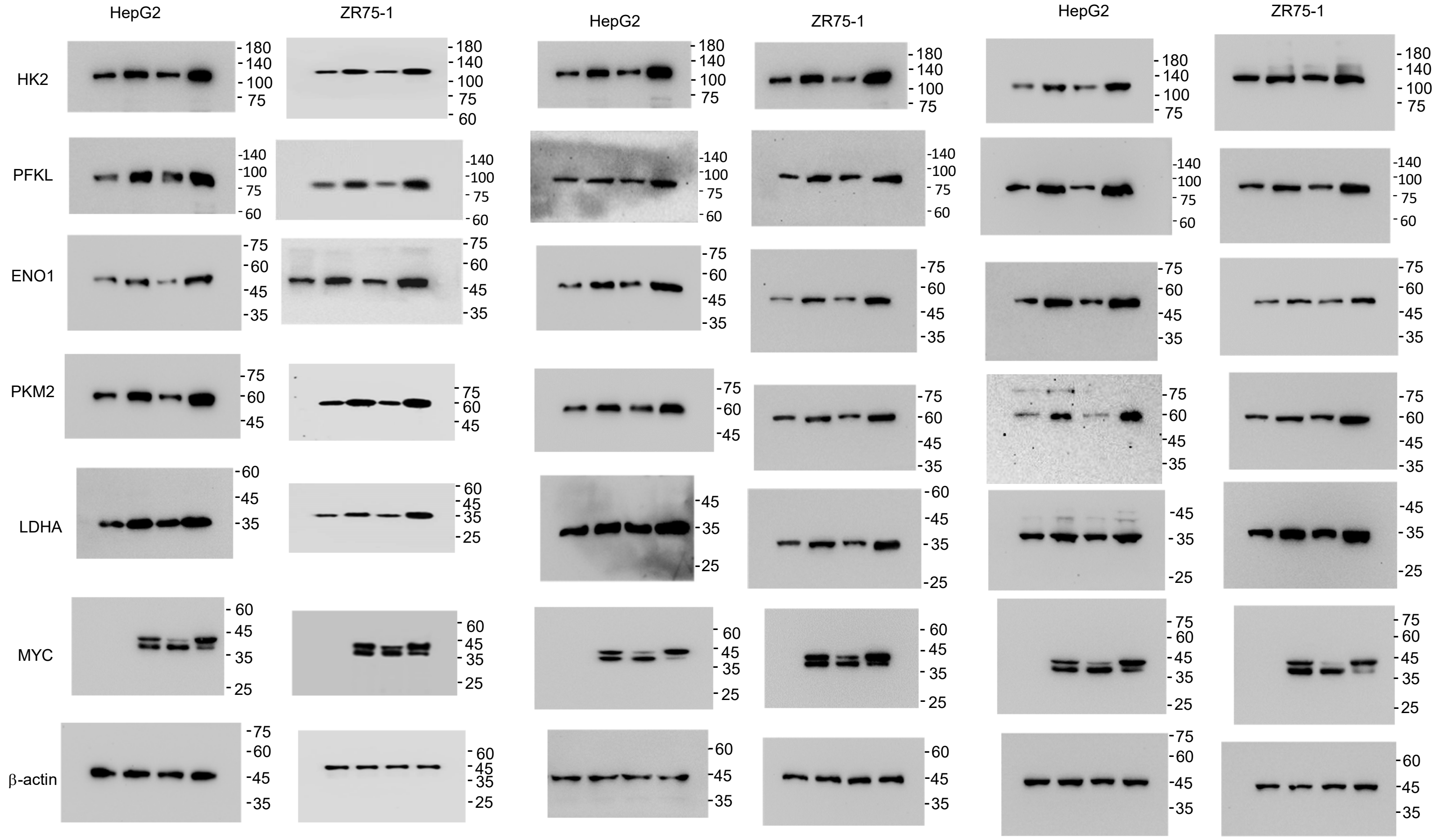

Fig. S6d

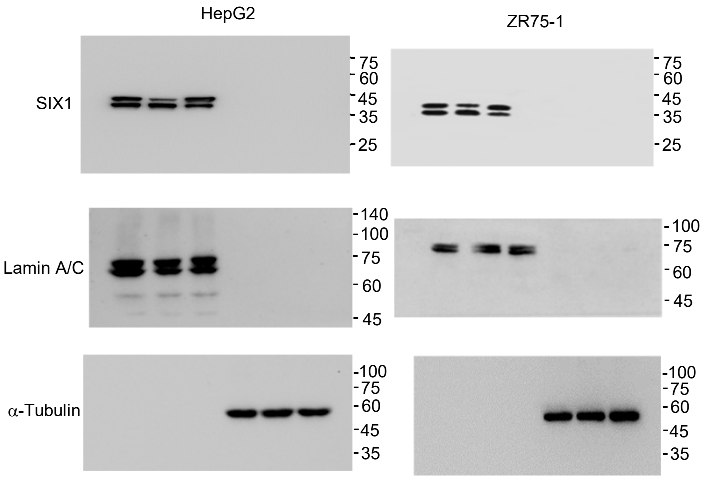

Fig. S6e

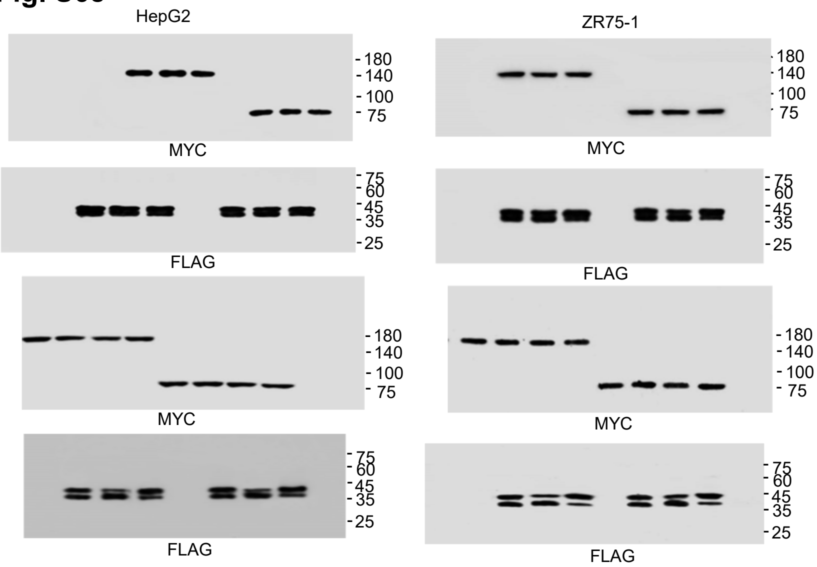

Fig. S6f

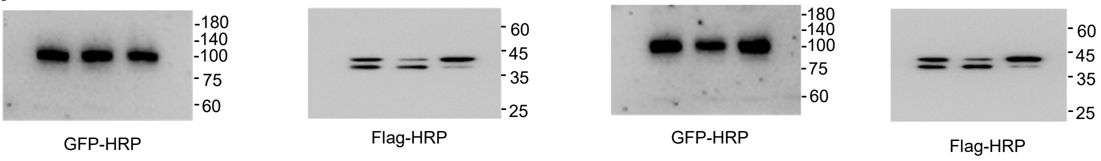

Supplement: Supplementary file 2 — Dataset 2 [file 41392_2024_2034_MOESM2_ESM.pdf]
