# Supplementary material for: Phosphorylation determines the glucose metabolism reprogramming and tumor-promoting activity of sine oculis homeobox 1
Source: Signal Transduct Target Ther. 2024 Dec 2;9:337. doi: 10.1038/s41392-024-02034-5 (PMC11609306; doi:10.1038/s41392-024-02034-5)
Supplement: Supplementary file 3 — Dataset 1 [file 41392_2024_2034_MOESM3_ESM.zip › Mascot Search Results/GPLTSSLVDLGS_Mascot Search Results Peptide View.htm]

Mascot Search Results: Peptide View


# Mascot Search Results

### Peptide View

MS/MS Fragmentation of **GPLTSSLVDLGS**  
Found in **SIX1\_HUMAN** in **SwissProt**, Homeobox protein SIX1 OS=Homo sapiens GN=SIX1 PE=1 SV=1  

Match to Query 2208: 1224.576448 from(613.295500,2+) rtinseconds(2489) index(11311)  
Title: Locus:1.1.1.2239.2 File:"8-7\_20151117\_1.wiff"  
Data file C:\Users\NCBA\Desktop\wiff-mgf\8-7\_20151117\_1.mgf

Click mouse within plot area to zoom in by factor of two about that point  
Or,  
 to 
 Da
     
  
Label all possible matches    
Label matches used for scoring   
Show Y-axis 


```
Monoisotopic mass of neutral peptide Mr(calc): 1224.5639
Fixed modifications: Carbamidomethyl (C) (apply to specified residues or termini only)
Variable modifications: 
S6     : Phospho (ST), with neutral losses 0.0000(shown in table), 97.9769
Ions Score: 52  Expect: 0.0023
Matches : 38/126 fragment ions using 69 most intense peaks   (help)
```

| # | b | b++ | b0 | b0++ | Seq. | y | y++ | y\*++ | y0 | y0++ | # |
| --- | --- | --- | --- | --- | --- | --- | --- | --- | --- | --- | --- |
| **1** | 58.0287 | 29.5180 |  |  | **G** |  |  |  |  |  | **12** |
| **2** | ***155.0815*** | 78.0444 |  |  | **P** | 1168.5497 | 584.7785 |  | 1150.5391 | 575.7732 | **11** |
| **3** | ***268.1656*** | 134.5864 |  |  | **L** | 1071.4969 | 536.2521 |  | 1053.4864 | 527.2468 | **10** |
| **4** | ***369.2132*** | 185.1103 | 351.2027 | 176.1050 | **T** | 958.4129 | 479.7101 |  | 940.4023 | 470.7048 | **9** |
| **5** | ***456.2453*** | 228.6263 | 438.2347 | 219.6210 | **S** | 857.3652 | 429.1862 |  | 839.3546 | 420.1810 | **8** |
| **6** | 623.2436 | 312.1255 | 605.2331 | 303.1202 | **S** | 770.3332 | 385.6702 |  | 752.3226 | 376.6649 | **7** |
| **7** | ***736.3277*** | 368.6675 | 718.3171 | 359.6622 | **L** | 603.3348 | 302.1710 |  | 585.3243 | 293.1658 | **6** |
| **8** | ***835.3961*** | 418.2017 | 817.3855 | 409.1964 | **V** | 490.2508 | 245.6290 |  | 472.2402 | 236.6237 | **5** |
| **9** | ***950.4231*** | 475.7152 | 932.4125 | 466.7099 | **D** | **391.1823** | 196.0948 |  | 373.1718 | 187.0895 | **4** |
| **10** | ***1063.5071*** | 532.2572 | 1045.4966 | 523.2519 | **L** | **276.1554** | 138.5813 |  | 258.1448 | 129.5761 | **3** |
| **11** | ***1120.5286*** | 560.7679 | 1102.5180 | 551.7626 | **G** | **163.0713** | 82.0393 |  | 145.0608 | 73.0340 | **2** |
| **12** |  |  |  |  | **S** | **106.0499** | 53.5286 |  | 88.0393 | 44.5233 | **1** |

NCBI **BLAST** search of GPLTSSLVDLGS  
(Parameters: blastp, nr protein database, expect=20000, no filter, PAM30)  
Other BLAST web gateways

**All matches to this query**

| Score | Mr(calc) | Delta | Sequence | Site Analysis |
| --- | --- | --- | --- | --- |
| 51.5 | 1224.5639 | 0.0125 | GPLTSSLVDLGS | Phospho S6 83.74% |
| 43.8 | 1224.5639 | 0.0125 | GPLTSSLVDLGS | Phospho S5 14.12% |
| 35.6 | 1224.5639 | 0.0125 | GPLTSSLVDLGS | Phospho T4 2.12% |
| 11.8 | 1224.5639 | 0.0125 | GPLTSSLVDLGS | Phospho S12 0.01% |
| 8.2 | 1224.6027 | -0.0262 | SYQSGDVPLIF |  |
| 5.5 | 1224.5614 | 0.0151 | LVNSWLPGML |  |
| 4.8 | 1224.5696 | 0.0068 | VDLDMKGINY |  |
| 4.8 | 1224.5928 | -0.0163 | DLGFRFDVAW |  |
| 4.1 | 1224.5427 | 0.0337 | LLQATQVNAW |  |
| 4.1 | 1224.5540 | 0.0225 | EVRIVASNAW |  |

|  |
| --- |
| **Mascot:** http://www.matrixscience.com/ |
