# Supplementary material for: Phosphorylation determines the glucose metabolism reprogramming and tumor-promoting activity of sine oculis homeobox 1
Source: Signal Transduct Target Ther. 2024 Dec 2;9:337. doi: 10.1038/s41392-024-02034-5 (PMC11609306; doi:10.1038/s41392-024-02034-5)
Supplement: Supplementary file 3 — Dataset 1 [file 41392_2024_2034_MOESM3_ESM.zip › Mascot Search Results/SPPQSPDQNSVL_Mascot Search Results Peptide View.htm]

Mascot Search Results: Peptide View


# Mascot Search Results

### Peptide View

MS/MS Fragmentation of **SPPQSPDQNSVL**  
Found in **SIX1\_HUMAN** in **SwissProt**, Homeobox protein SIX1 OS=Homo sapiens GN=SIX1 PE=1 SV=1  

Match to Query 3105: 1347.570588 from(674.792570,2+) rtinseconds(1408) index(6573)  
Title: Locus:1.1.1.1715.9 File:"8-7\_20151117\_1.wiff"  
Data file C:\Users\NCBA\Desktop\wiff-mgf\8-7\_20151117\_1.mgf

Click mouse within plot area to zoom in by factor of two about that point  
Or,  
 to 
 Da
     
  
Label all possible matches    
Label matches used for scoring   
Show Y-axis 


```
Monoisotopic mass of neutral peptide Mr(calc): 1347.5708
Fixed modifications: Carbamidomethyl (C) (apply to specified residues or termini only)
Variable modifications: 
S5     : Phospho (ST), with neutral losses 0.0000(shown in table), 97.9769
Ions Score: 48  Expect: 0.0054
Matches : 43/182 fragment ions using 51 most intense peaks   (help)
```

| # | b | b++ | b\* | b\*++ | b0 | b0++ | Seq. | y | y++ | y\* | y\*++ | y0 | y0++ | # |
| --- | --- | --- | --- | --- | --- | --- | --- | --- | --- | --- | --- | --- | --- | --- |
| **1** | 88.0393 | 44.5233 |  |  | 70.0287 | 35.5180 | **S** |  |  |  |  |  |  | **12** |
| **2** | 185.0921 | 93.0497 |  |  | 167.0815 | 84.0444 | **P** | 1261.5460 | 631.2767 | 1244.5195 | 622.7634 | 1243.5355 | 622.2714 | **11** |
| **3** | 282.1448 | 141.5761 |  |  | 264.1343 | 132.5708 | **P** | 1164.4933 | 582.7503 | 1147.4667 | 574.2370 | 1146.4827 | 573.7450 | **10** |
| **4** | ***410.2034*** | 205.6053 | 393.1769 | 197.0921 | 392.1928 | 196.6001 | **Q** | 1067.4405 | 534.2239 | 1050.4139 | 525.7106 | 1049.4299 | 525.2186 | **9** |
| **5** | ***577.2018*** | 289.1045 | 560.1752 | 280.5912 | 559.1912 | 280.0992 | **S** | 939.3819 | 470.1946 | 922.3554 | 461.6813 | 921.3714 | 461.1893 | **8** |
| **6** | ***674.2545*** | 337.6309 | 657.2280 | 329.1176 | 656.2440 | 328.6256 | **P** | **772.3836** | 386.6954 | 755.3570 | 378.1821 | 754.3730 | 377.6901 | **7** |
| **7** | ***789.2815*** | 395.1444 | 772.2549 | 386.6311 | 771.2709 | 386.1391 | **D** | 675.3308 | 338.1690 | 658.3042 | 329.6558 | 657.3202 | 329.1638 | **6** |
| **8** | ***917.3401*** | 459.1737 | 900.3135 | 450.6604 | 899.3295 | 450.1684 | **Q** | 560.3039 | 280.6556 | 543.2773 | 272.1423 | 542.2933 | 271.6503 | **5** |
| **9** | ***1031.3830*** | 516.1951 | 1014.3564 | 507.6819 | 1013.3724 | 507.1898 | **N** | 432.2453 | 216.6263 | 415.2187 | 208.1130 | 414.2347 | 207.6210 | **4** |
| **10** | ***1118.4150*** | 559.7111 | 1101.3885 | 551.1979 | 1100.4044 | 550.7059 | **S** | 318.2023 | 159.6048 |  |  | 300.1918 | 150.5995 | **3** |
| **11** | ***1217.4834*** | 609.2453 | 1200.4569 | 600.7321 | 1199.4729 | 600.2401 | **V** | **231.1703** | 116.0888 |  |  |  |  | **2** |
| **12** |  |  |  |  |  |  | **L** | **132.1019** | 66.5546 |  |  |  |  | **1** |

NCBI **BLAST** search of SPPQSPDQNSVL  
(Parameters: blastp, nr protein database, expect=20000, no filter, PAM30)  
Other BLAST web gateways

**All matches to this query**

| Score | Mr(calc) | Delta | Sequence | Site Analysis |
| --- | --- | --- | --- | --- |
| 48.4 | 1347.5708 | -0.0002 | SPPQSPDQNSVL | Phospho S5 82.37% |
| 41.7 | 1347.5708 | -0.0002 | SPPQSPDQNSVL | Phospho S1 17.61% |
| 12.1 | 1347.5384 | 0.0322 | TESLFNGSHYL |  |
| 11.7 | 1347.5716 | -0.0010 | MKRYCPNSVL |  |
| 10.8 | 1347.5708 | -0.0002 | SPPQSPDQNSVL | Phospho S10 0.01% |
| 10.6 | 1347.6071 | -0.0365 | APNTPINQNVSL |  |
| 10.6 | 1347.5716 | -0.0010 | MKRYCPNSVL |  |
| 10.4 | 1347.6033 | -0.0327 | VSNLYVISQML |  |
| 9.2 | 1347.6071 | -0.0365 | APNTPINQNVSL |  |
| 8.6 | 1347.5595 | 0.0110 | DGPDPEKDPSVL |  |

|  |
| --- |
| **Mascot:** http://www.matrixscience.com/ |
