# Supplementary figures and images for: Phosphorylation determines the glucose metabolism reprogramming and tumor-promoting activity of sine oculis homeobox 1
Source: Signal Transduct Target Ther. 2024 Dec 2;9:337. doi: 10.1038/s41392-024-02034-5 (PMC11609306; doi:10.1038/s41392-024-02034-5)

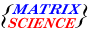

Supplement: Supplementary file 3 — Dataset 1 [file 41392_2024_2034_MOESM3_ESM.zip › Mascot Search Results/GPLTSSLVDLGS_Mascot Search Results Peptide View_files/88x31_logo_white.gif]

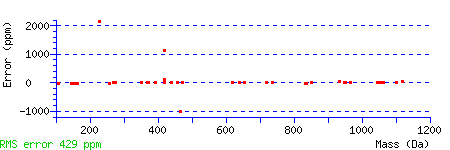

Supplement: Supplementary file 3 — Dataset 1 [file 41392_2024_2034_MOESM3_ESM.zip › Mascot Search Results/GPLTSSLVDLGS_Mascot Search Results Peptide View_files/mass_error(1).pl]

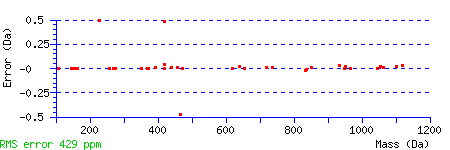

Supplement: Supplementary file 3 — Dataset 1 [file 41392_2024_2034_MOESM3_ESM.zip › Mascot Search Results/GPLTSSLVDLGS_Mascot Search Results Peptide View_files/mass_error.pl]

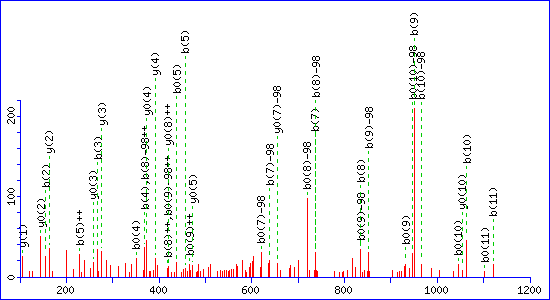

Supplement: Supplementary file 3 — Dataset 1 [file 41392_2024_2034_MOESM3_ESM.zip › Mascot Search Results/GPLTSSLVDLGS_Mascot Search Results Peptide View_files/msms_gif.pl]

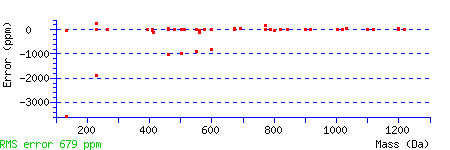

Supplement: Supplementary file 3 — Dataset 1 [file 41392_2024_2034_MOESM3_ESM.zip › Mascot Search Results/SPPQSPDQNSVL_Mascot Search Results Peptide View_files/mass_error(1).pl]

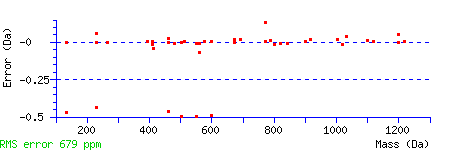

Supplement: Supplementary file 3 — Dataset 1 [file 41392_2024_2034_MOESM3_ESM.zip › Mascot Search Results/SPPQSPDQNSVL_Mascot Search Results Peptide View_files/mass_error.pl]

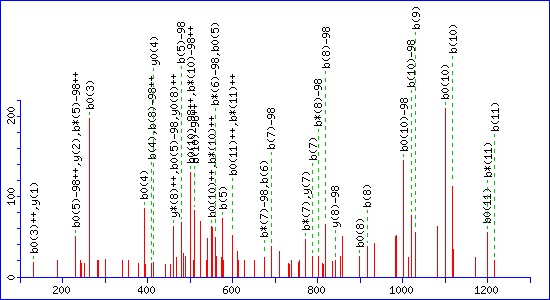

Supplement: Supplementary file 3 — Dataset 1 [file 41392_2024_2034_MOESM3_ESM.zip › Mascot Search Results/SPPQSPDQNSVL_Mascot Search Results Peptide View_files/msms_gif.pl]
